# RAC3 Promotes Proliferation, Migration and Invasion via PYCR1/JAK/STAT Signaling in Bladder Cancer

**DOI:** 10.3389/fmolb.2020.00218

**Published:** 2020-08-31

**Authors:** Chuanyu Cheng, Dongkui Song, Yudong Wu, Bingqian Liu

**Affiliations:** Department of Urology, The First Affiliated Hospital of Zhengzhou University, Zhengzhou, China

**Keywords:** RAC3, BCa, JAK/STAT pathway, PYCR1, oncogene

## Abstract

**Background:**

Bladder cancer (BCa) represents one of the most common malignant cancers with high incidence and mortality rates globally. Dysregulation of gene expression has been shown to play critical roles in cancer progression. RAC3 is up-regulated to play an oncogenic role in several cancers, however, the underlying mechanism of RAC3 in BCa is yet to be elucidated. Therefore, this study aimed to investigate the function and mechanism of RAC3 in BCa.

**Methods:**

Bioinformatics analysis was employed to demonstrate the expression of RAC3 and PYCR1 in BCa tissues, as well as, its correlation with the overall survival rate of BCa patients. RT-qPCR was performed to detect and quantify the mRNA levels of RAC3 and PYCR1 in BCa cells and immortalized human bladder epithelial cells. MTT, colony formation and Transwell assays were employed to determine cell proliferation, migration, and invasion. Western blotting was performed to detect and quantity proteins expressed.

**Results:**

Bioinformatics analysis showed that RAC3 was up-regulated in BCa tissues when compared to normal tissues. Patients with up-regulated RAC3 expression had lower overall survival than patients with down-regulated RAC3 expression. The mRNA level of RAC3 was higher in BCa cells than in immortalized human bladder epithelial cell. RAC3 promoted cell proliferation, migration, and invasion by activating Janus kinases (JAKs) and signal transducers and activators of transcription (STATs) signaling. Notably, RAC3 up-regulated PYCR1, which is positively correlated with RAC3, and thus played an oncogenic role in BCa cells. Moreover, we demonstrated that RAC3 overexpression activated JAK/STAT signaling via PYCR1 axis.

**Conclusion:**

RAC3 promoted cell proliferation, migration, and invasion. This is likely due to its role in activating JAK/STAT signaling, which was mediated by PYCR1. This study provides a novel biomarker and target for diagnostic or therapeutic intervention for BCa.

## Introduction

Bladder Cancer (BCa) represents one of the most common malignant cancers with high incidence and mortality rates globally (Pilleron et al., 2019). According to the global cancer statistics in 2018, approximately 549,393 new cases accounting for 3.0% of the total cancer cases were diagnosed globally; and 199,922 deaths accounting for 3.1% of mortality were recorded globally (Bray et al., 2018; Ferlay et al., 2019). Surgery, radiotherapy, chemotherapy, and immunotherapy are among the major strategies for the treatment of BCa patients. Unfortunately, the 5-year overall survival rate has remained poor in the past decade (Babjuk, 2017; Draeger et al., 2018). In addition, the post-operative quality of life of patients with BCa is unsatisfactory (Mason et al., 2018). Therefore, there is an urgent need for early diagnostic markers, as well as, efficient therapeutic targets for BCa.

Rac family of small GTPase 3 (RAC3), also called ras-related C3 botulinum toxin substrate 3, is a member of the Rho GTPases family, including RAC1, RAC2, and RAC3, which plays a critical role in cancer progression (Pai et al., 2010; Wertheimer et al., 2012; Kazanietz and Caloca, 2017; Maldonado and Dharmawardhane, 2018). RAC3 is shown to be primitively expressed in a wide range of tissues, especially in neuronal tissues, under non-pathological circumstances (Haataja et al., 1997; Corbetta et al., 2009; Vaghi et al., 2014). Recently, research has demonstrated that RAC3 is up-regulated to serve as an oncogene by promoting cell proliferation and cell aggressiveness in several cancers including breast cancer (Chan et al., 2005; Gest et al., 2013), prostate cancer (Engers et al., 2007), brain cancer (Hwang et al., 2005), and lung cancer (Liu et al., 2015; Zhang et al., 2017). According to the bioinformatics analysis results from The Cancer Genome Atlas dataset, RAC3 is highly expressed in BCa tissues when compared with the normal tissues, and as such forecasts a high-risk signature for BCa of clinical relevance (Qiu et al., 2020). In literature, studies concerning the role of RAC3 in BCa have not been reported. Therefore, further investigation on the function and underlying mechanism of RAC3 in BCa is urgently required.

In this study, we demonstrated that RAC3 was up-regulated in BCa tissues and cells, and its overexpression promoted the proliferation, migration and invasion of BCa cells via the activation of JAK/STAT3 signaling. Further investigation showed that PYCR1, which was up-regulated by RAC3, also functioned as an oncogene in RAC3-mediated activation of JAK/STAT3 signaling in BCa. These results demonstrated that RAC3 might be a novel biomarker and target for BCa diagnosis and treatment.

## Materials and Methods

### Cell Culture and Transfection

Bladder Cancer cell lines (J82, T24, UMUC3, BC-5637 and BIU-87) and immortalized human bladder epithelial cells (SV-HUC-1) were purchased from American Type Culture Collection (Manassas, VA, United States). Cells were cultured in Dulbecco’s modified eagle medium (DMEM, Gibco, Carlsbad, CA, United States) containing 10% fetal bovine serum (FBS, Gibco, Carlsbad, CA, United States) and 100 U/ml penicillin-streptomycin, and was maintained under the sterile condition at 37°C with 5% CO_2_.

The full-length of RAC3 and PYCR1 were synthetized and cloned into pcDNA3 vector by GENECHEM (Shanghai, China) for the generation of pcDNA3-RAC3 (RAC3) and pcDNA3-PYCR1 (PYCR1), respectively. In addition, RAC3 and PYCR1 knockdown plasmids, pshR-RAC3 (shR-RAC3) and pcDNA3-PYCR1 (PYCR1), were constructed by GENECHEM (Shanghai, China). The aforementioned plasmids and their control empty vectors, pcDNA3 vector (Vec) or p-silencer 2.1-U6 neo (shR-NC), were transfected into BCa cells using Lipofectamine 2000 (Thermo Fisher Scientific, Carlsbad, CA, United States) based on the manufacturer’s protocols.

### RNA Extraction and Reverse Transcriptase Quantitative-Polymerase Chain Reaction (RT-qPCR)

Total RNAs was isolated using TRIzol Reagent (Thermo Fisher Scientific, Carlsbad, CA, United States), and was reversely transcribed into cDNA using Maxima H Minus First Strand cDNA Synthesis Kit (Thermo Fisher Scientific, Carlsbad, CA, United States). RT-qPCR was performed using DyNAmo Flash SYBR Green qPCR Kit (Thermo Fisher Scientific, Carlsbad, CA, United States) and Bio-Rad^TM^ CFX96 System (Bio-Rad, United States). The primers used for the qPCR were as follows: for RAC3, the forward primer was 5′- TCCCCAC CGTTTTTGACAACT-3′, while the reverse primer was R: 5′- GCACGAACATTCTCGAAGGAG-3′; for PYCR1, the forward primer was F: 5′- TGGCTGCCCACAAGATAATGG-3′, while the reverse primer was R: 5′- CGTGACGGCATCAATCAGGT-3′; for GAPDH, the forward primer was F: 5′- CTGGGCTACACTGAGCACC -3′, while the reverse primer was R: 5′- AAGTGGTCGTTGAGGGCAATG -3′. GAPDH was used as the internal reference, and the data was calculated by 2^–ΔΔ*ct*^ method.

### Rac3 Activity Assay

Cells were grown on 100-mm dishes and the active form of Rac3 was detected using a PBD-GTPγ-Rac3 pull down assay. The cell lysates (about 0.5–1 mg/ml) were incubated with GST-PAK-PBD essentially as described by Benard et al. (1999). GST-PAK-PBD, which conjugated to agarose, was incubated for 1 h to bind Rac3-GTP. Then, the mixture was centrifuged, and washed three times with lysis buffer. The proteins were separated by SDS-PAGE, transferred to nitrocellulose membranes, and probed with a Rac3 antibody. Total cell lysates were also probed separately with anti-Rac3 and anti-GAPDH antibodies to confirm equal loading.

### MTT Assay

3-(4,5-dimethylthiazol-2-yl)-2, 5-diphenyltetrazolium bromide (MTT) was used to detect the cell viability. In brief, 2 × 10^3^ cells were seeded onto a 96-well plate for 18–24 h. Cells were transfected with the constructed plasmids and cultured for 24, 48, and 72 h. At 0, 24, 48, and 72 h after transfection, 100 μL MTT solution (1 mg/mL, Beyotime, China) was added to each well, and was allowed to incubate for another 4 h. Subsequently, the medium was carefully removed and 100 μL dimethyl sulfoxide was added into each well to resolve the MTT formazan crystals. The optical density value was detected at a wavelength of 570 nm using a scan-enabled multi-well spectrophotometer.

### Colony Formation Assay

Colony formation assay was utilized to detect the cell proliferative ability. BCa cells were seeded onto 6-well plates and transfected with constructed plasmids. After culturing for 24 h, cells were digested using 0.05% Trypsin-EDTA (Thermo Fisher Scientific, Carlsbad, CA, United States). BCa cells (300 per well) were seeded onto 12-well plates and then cultured for about 2 weeks. The medium was carefully removed and cells were washed with 1 × PBS, and fixed with 4% paraformaldehyde. Finally, cells were stained with 0.2% crystal violet for 10 min. The clones were observed under an inverted microscope, and the number of clones was quantified using ImageJ software.

### Transwell Assay

Transwell assay was employed to detect cell migration and invasion abilities. In brief, BCa cells were transfected with the constructed plasmids and incubated for 24 h. Cells were digested using 0.05% Trypsin-EDTA and re-suspended in a serum-free DMEM. 100 μL cell suspension (5 × 10^4^ cells for migration and 8 × 10^4^ cells for invasion) was seeded onto the upper chambers (8 μm pore size, BD Biosciences, CA, United States), which was coated without Matrigel (BD Biosciences) for the cell migration assay and with Matrigel for the cell invasion assay. The bottom chambers were filled with 600 μL DMEM containing 10% FBS, and was cultured for 36 h. Thereafter, cells on the upper surface were wiped using cotton swabs, and the migrated cells on the bottom surface were fixed with 4% polyformaldehyde. After washed with 1 × PBS, cells were stained with 0.2% crystal violet for 10 min. The migrated or invaded cells were observed under an inverted microscope, and the number of cells was quantified using ImageJ software.

### Western Blot

Proteins were extracted from BCa cells using Radio-Immunoprecipitation Assay (Thermo Fisher Scientific, Carlsbad, CA, United States). The extracted proteins were quantified using the Pierce^TM^ Rapid Gold BCA Protein Assay Kit (Thermo Fisher Scientific, Carlsbad, CA, United States). The extracted proteins were separated by SDS-PAGE and transferred to polyvinylidene difluoride membranes (Thermo Fisher Scientific, Carlsbad, CA, United States). Subsequently, the membranes were blocked with 5% skim milk and were incubating with primary antibodies overnight at 4°C. After washing with 1 × TBST, the membranes were incubated with horseradish peroxidase-conjugated secondary antibodies (Thermo Fisher Scientific, Carlsbad, CA, United States) at room temperature for 2 h. The bands on the membranes were then visualized using Pierce^TM^ enhanced chemiluminescence (ECL) substrate (Thermo Fisher Scientific, Carlsbad, CA, United States). The gray value of the bands was quantified using ImageJ software, with GAPDH as the internal reference. The primary antibodies used in this study is as follows: RAC3 (1:1000, ab124943, Abcam, Inc., Cambridge, MA, United States), JAK2 (1:2000, ab108596, Abcam, Inc., Cambridge, MA, United States), p-JAK2 (phospho Y1007 + Y1008, 1:1000, ab32101, Abcam, Inc., Cambridge, MA, United States), STAT3 (1:500, Cat # PA5-84386, Thermo Fisher Scientific, Carlsbad, CA, United States), p-STAT3 (1:500, Cat #44-380G, Thermo Fisher Scientific, Carlsbad, CA, United States), c-Myc (1:500, Cat #PA5-85185, Thermo Fisher Scientific, Carlsbad, CA, United States), and GAPDH (1:10000, ab181602, Abcam, Inc., Cambridge, MA, United States).

### Statistical Analysis

Data were expressed as mean ± standard deviation. All experiments were repeated at least three times independently. Data analysis was carried using SPSS 22.0 and GraphPad Prism 8 statistical software packages. Differences between two groups and among multiple groups were analyzed by Student’s independent *t*-test and one-way analysis of variance, respectively. *p* < 0.05 was considered as significant.

## Results

### RAC3 Was Up-Regulated in BCa

**FIGURE 1:**
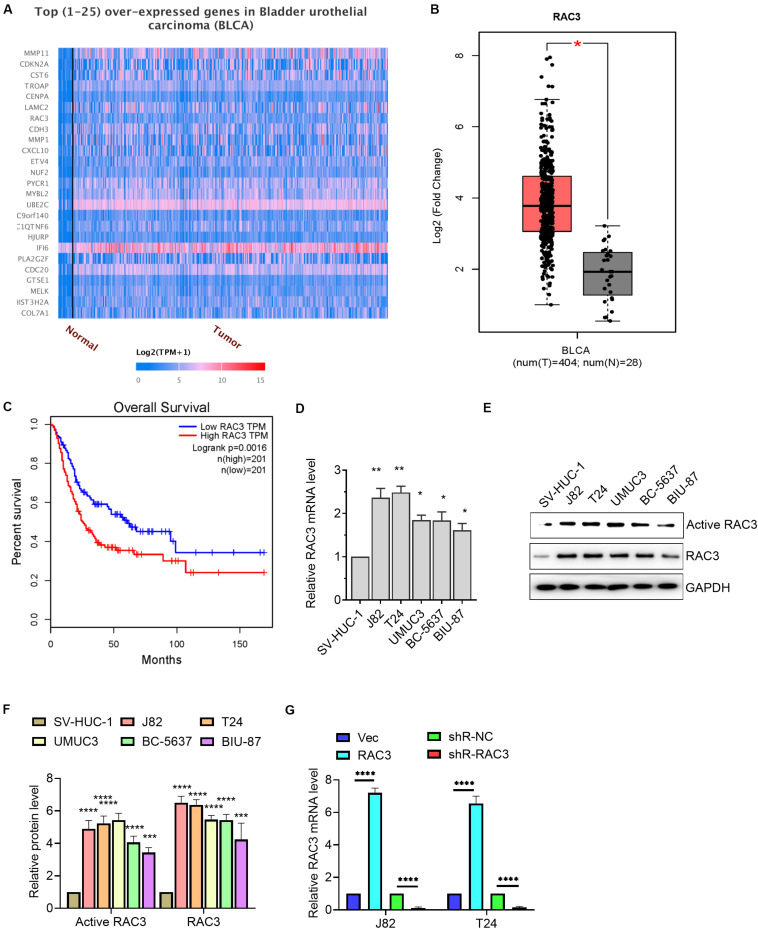
RAC3 was overexpressed in BCa. **(A)** Bioinformatics analysis (UALCAN database, http://ualcan.path.uab.edu/index.html) was employed to analyze the top 25 overexpressed genes in BCa. **(B)** Bioinformatics analysis (GEPIA database, http://gepia.cancer-pku.cn/) was employed to analyze the transcriptional level of RAC3 in BCa tissues (T) and normal bladder tissues (N). **(C)** Bioinformatics analysis from GEPIA database showed the overall survival rate of BCa patients with high or low RAC3 transcriptional level. **(D)** RT-qPCR evaluated the transcriptional level of RAC3 in BCa cell lines (J82, T24, UMUC3, BC-5637, and BIU-87) and immortalized human bladder epithelial cells (SV-HUC-1). **(E,F)** Cell lysates were used to detect Rac3 activity by using a PBD-GTPγ-Rac3 pull down assay. Total cell lysates were analyzed by immunoblotting with Rac3, and GAPDH antibodies. Activated Rac3 levels were quantified by Image J software. **(G)** J82 and T24 cells were transfected with empty pcDNA3 vector (Vec), pcDNA3-RAC3 (RAC3), p-silencer 2.1-U6 neo (shR-NC), and pshR-RAC3 (shR-RAC3) and the mRNA level of RAC3 was detected by RT-PCR. *p < 0.05; **p < 0.01; ***p < 0.001; ****p < 0.0001.

UALCAN database, which contained TCGA level 3 RNA-seq and clinical data from 31 cancer types, was an user friendly web server to identify the top over- and under-expressed (up and down-regulated) genes in individual cancer types (Chandrashekar et al., 2017). GEPIA was a friendly web server for cancer and normal gene expression profiling and interactive analyses, including differential expression analysis, profiling plotting, correlation analysis, patient survival analysis, similar gene detection and dimensionality reduction analysis (Tang et al., 2017). Bioinformatics analysis of data from UALCAN database revealed that RAC3 is among the overexpressed genes in BCa (Figure 1A). We further analyzed the data using GEPIA database and confirmed that RAC3 was up-regulated in the BCa tissues when compared with the normal bladder tissues (Figure 1B). Subsequently, we found that patients with higher RAC3 expression had shorter overall survival than patients with lower RAC3 expression (Figure 1C). Furthermore, we determined the transcriptional level of RAC3 in BCa cell lines (J82, T24, UMUC3, BC-5637, and BIU-87 cells) and immortalized human bladder epithelial cell (SV-HUC-1) using RT-qPCR technique. Results revealed that the relative level of RAC3 mRNA was dramatically increased in five BCa cell lines when compared to the immortalized human bladder epithelial cell (SV-HUC-1) (Figure 1D). Moreover, we detected the levels of RAC3-active form for all the Bca cell lines and found a positive correlation between the RAC3 active form and its mRNA levels (Figures 1E,F). In addition, RAC3 overexpressed or silenced plasmids were transfected into J82 and T24 cells. RT-qPCR results revealed that RAC3 mRNA level was significantly increased following transfection with pcDNA3-RAC3 plasmid, whereas, RAC3 mRNA level was reduced following transfection with pshR-RAC3 plasmid (Figure 1G). We also detected the mRNA and protein levels of RAC1 and RAC2 after silencing of RAC3 by shR-RAC3, and results showed that silencing of RAC3 had a significantly inhibitory effect on RAC3 mRNA and protein expression, with no significant effect on RAC1 and RAC2 mRNA and protein levels (Supplementary Figure S1).

### RAC3 Enhanced the Proliferation, Migration, and Invasion of BCa Cells

**FIGURE 2:**
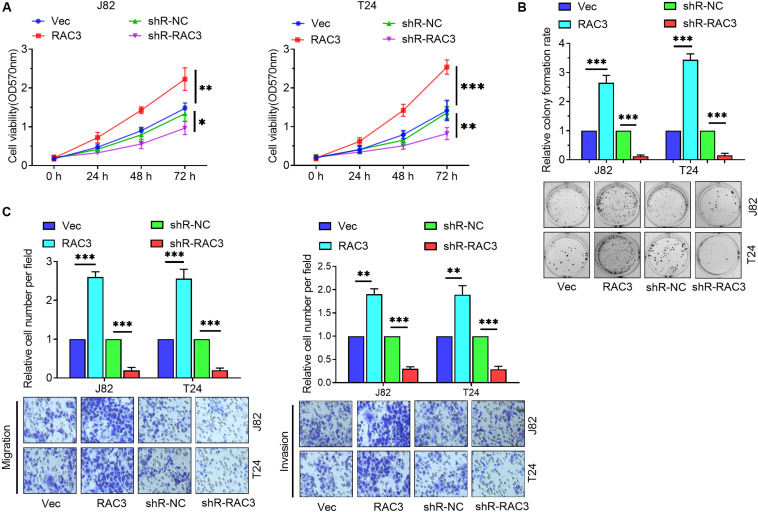
RAC3 promoted the proliferation, migration and invasion of BCa cells. **(A)** MTT assay was employed to detect the cell viabilities of BCa cells. **(B)** Colony formation assay was employed to evaluate the proliferation of BCa cells. **(C)** Transwell assays were employed to measure the migratory and invasive abilities of BCa cells. ^∗^*p* < 0.05; ^∗∗^*p* < 0.01; ^∗∗∗^*p* < 0.001.

To further investigate the role of RAC3 in BCa, gain of function, as well as loss of function assays were performed to evaluate the level of expression of RAC3 on BCa cells. Firstly, the MTT assay revealed that up-regulation of RAC3 increased cell viabilities and down-regulation of RAC3 inhibited (Figure 2A). Colony formation assays revealed that forced expression of RAC3 significantly enhanced colony number, whereas, down-regulation of RAC3 showed an opposite effect in BCa cells (Figure 2B). Furthermore, the role of high expression of RAC3 on cell migration and invasion were also investigated. Transwell assay results indicate that the migratory and invasive ability of the BCa cells increased markedly when RAC3 was overexpressed, whereas, the opposite effect was observed when RAC3 was silenced (Figure 2C). Taken together, these results indicate that overexpression of RAC3 could promote cell proliferation, migration, and invasion in BCa.

### RAC3 Activated JAK/STAT Signaling in Bca Cells

**FIGURE 3:**
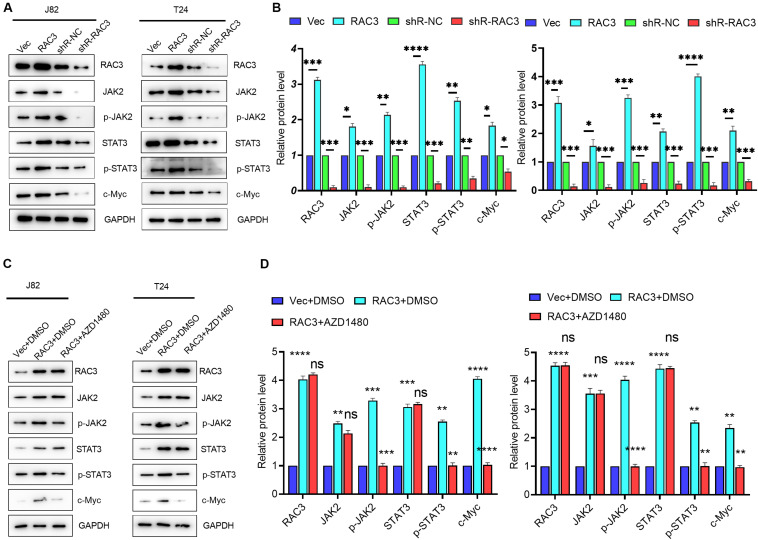
RAC3 promoted JAK/STAT signaling in BCa cells. **(A)** BCa cells were transfected with empty pcDNA3 vector (Vec), pcDNA3-RAC3 (RAC3), shR-NC, and shR-RAC3, western blot was used to detect the protein levels of RAC3, JAK2, p-JAK2, STAT3, p-STAT3 and c-Myc. **(B)** Quantification of the protein expression of RAC3, JAK2, p-JAK2, STAT3, p-STAT3 and c-Myc were detected by WB. The proteins were normalizing to the vector and the shR control separately. **(C)** BCa cells were transfected with empty pcDNA3 vector (Vec) + DMSO, pcDNA3-RAC3 (RAC3) + DMSO, and pcDNA3-RAC3 (RAC3) + AZD1480 (5 μM), and western blot analysis was used to detect and quantify the protein levels of RAC3, JAK2, p-JAK2, STAT3, p-STAT3 and c-Myc. **(D)** Quantification of the protein expression of RAC3, JAK2, p-JAK2, STAT3, p-STAT3, and c-Myc were detected by WB. The proteins were normalizing to the vector + DMSO. All experiments were replicated three times. *p < 0.05; **p < 0.01; ***p < 0.001; ****p < 0.0001; ns - not significant.

It is well known that JAK/STAT signaling, an evolutionarily conserved signaling cascade, plays a pivotal role in the regulation of various human diseases including cancer (Pencik et al., 2016; Groner and von Manstein, 2017). To investigate whether RAC3 affected the JAK/STAT pathway, Western blot analysis was employed. Results revealed that the overexpression of RAC3 markedly enhanced protein levels of JAK2 and phosphorylated JAK2 (Figures 3A,B), a member of protein-tyrosine kinase JAK protein family, which could activate the STATs transcription factor. On the contrary, knockdown of RAC3 markedly reduced the protein levels of JAK2 and phosphorylated JAK2 (Figures 3A,B). Moreover, the expression and phosphorylation of STAT3 were increased and the expression of STAT3-dependent gene, c-Myc, was enhanced by the overexpression of RAC3, however, this result was reversed by silencing RAC3 (Figures 3A,B). To further investigate the molecular mechanism of JAK/STAT pathway regulation by RAC3, AZD1480, a small inhibitor molecule of JAK/STAT signaling, was used to detect the effect on RAC3 over-expressing cells. Results showed that AZD1480 treatment significantly inhibited the phosphorylation of JAK2 and STAT3 induced by the overexpression of RAC3, indicating a repression of JAK/STAT signaling (Figures 3C,D). These results further suggest that JAK/STAT pathway was activated by overexpression of RAC3 in BCa cells.

### RAC3 Up-Regulated the Expression of PYCR1 in BCa Cells

**FIGURE 4:**
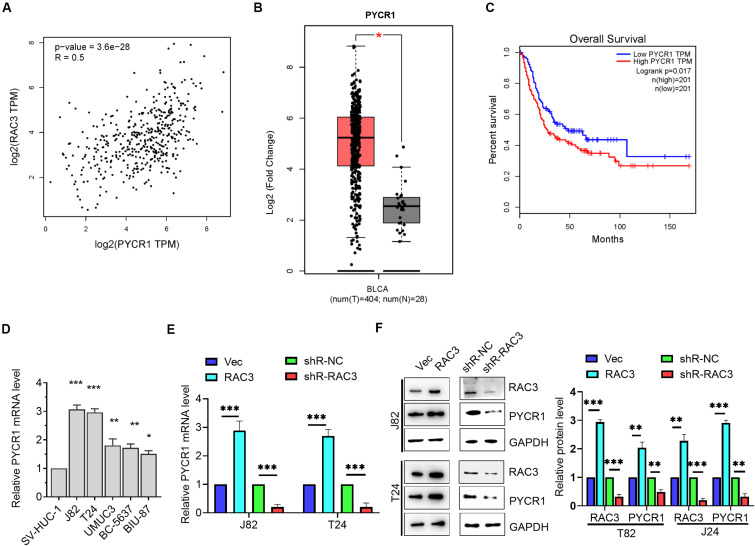
RAC3 promoted the expression of PYCR1 in BCa cells. **(A)** Bioinformatics analysis from GEPIA database showed a positive correlation between RAC3 and PYCR1. **(B)** Bioinformatics analysis from GEPIA database was utilized to analyze the transcriptional level of PYCR1 in BCa tissues (T) and normal bladder tissues (N). **(C)** The overall survival rate of BCa patients with high or low PYCR1 transcriptional level was predicted by bioinformatics analysis from GEPIA database. **(D)** RT-qPCR evaluated the transcriptional level of PYCR1 in BCa cell lines (J82, T24, UMUC3, BC-5637, and BIU-87) and immortalized human bladder epithelial cells (SV-HUC-1). **(E)** J82 and T24 cells were transfected with empty pcDNA3 vector (Vec), pcDNA3-RAC3 (RAC3), p-silencer 2.1-U6 neo (shR-NC), and pshR-RAC3 (shR-RAC3) and the mRNA level of PYCR1 was detected by RT-qPCR. **(F)** Western blot analysis was used to detect and quantify the protein levels of RAC3 and PYCR1. The proteins were normalizing to the vector and the shR control separately. All experiments were replicated three times. *p < 0.05; **p < 0.01; ***p < 0.001.

To further investigate the molecular mechanism of RAC3, we observed a positive correlation between RAC3 and pyrroline-5-carboxylate reductase (PYCR1) using bioinformatics analysis from GEPIA database (Figure 4A). We further observed that PYCR1 was up-regulated in BCa tissues when compared to normal bladder tissues using GEPIA database (Figure 4B). Furthermore, bioinformatics analysis revealed that patients with higher PYCR1 expression had short overall survival rate when compared to patients with lower PYCR1 expression (Figure 4C). The transcriptional level of PYCR1 in five BCa cell lines and immortalized human bladder epithelial cell were detected by RT-qPCR. Results indicate that PYCR1 mRNA was significantly up-regulated in the five BCa cell lines when compared to the immortalized human bladder epithelial cell (SV-HUC-1) (Figure 4D). In addition, we demonstrated that the overexpression of RAC3 significantly increased the mRNA and protein levels of PYCR1, whereas, knockdown of RAC3 yielded an opposite result (Figures 4E,F). These results indicate that the expression of PYCR1 was up-regulated in response to RAC3 in BCa cells.

### PYCR1 Enhanced the Proliferation, Migration, and Invasion of BCa Cells

**FIGURE 5:**
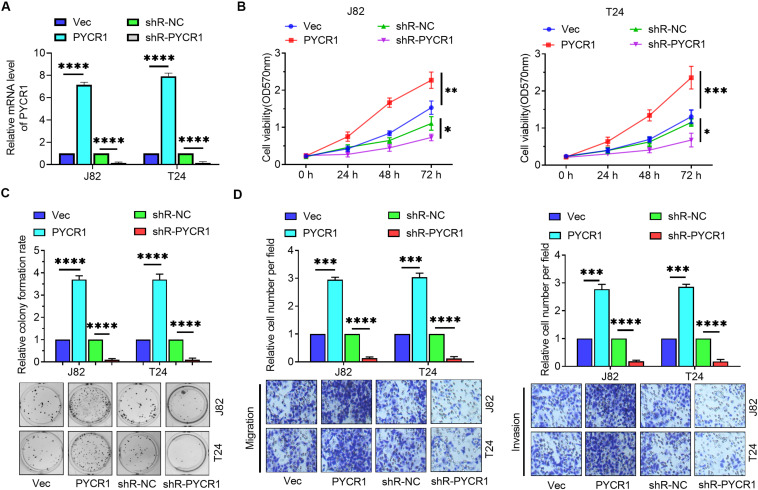
PYCR1 promoted the proliferation, migration and invasion of BCa cells. **(A)** J82 and T24 cells were transfected with empty pcDNA3 vector (Vec), pcDNA3-PYCR1 (PYCR1), p-silencer 2.1-U6 neo (shR-NC), and pshR-PYCR1 (shR-PYCR1) and the mRNA level of PYCR1 was detected by RT-qPCR. **(B)** MTT assay was employed to detect the viability of BCa cells. **(C)** Colony formation assay was employed to evaluate the proliferation of BCa cells. **(D)** Transwell assays were employed to measure the migratory and invasive abilities of BCa cells. ^∗^*p* < 0.05; ^∗∗^*p* < 0.01; ^∗∗∗^*p* < 0.001; ^****^p < 0.0001.

To further investigate the role of PYCR1 in BCa, PYCR1 overexpressed or silenced plasmids were transfected into J82 and T24 cells. RT-qPCR confirmed the effectiveness of these plasmids (Figure 5A). MTT assay, colony formation assay, and Transwell assays were employed to determine the role of PYCR1 in BCa cells. MTT assay results revealed that overexpression of PYCR1 promoted cell viability, whereas, down-regulation of PYCR1 expression yielded an opposite result (Figure 5B). In addition, PYCR1 up-regulation markedly increased the colony number, whereas, an opposite effect was observed by the down-regulation of PYCR1 in BCa cells (Figure 5C). Transwell assay results revealed that the overexpression of PYCR1 significantly increased migration and invasion in BCa cells, whereas, silencing of PYCR1 yielded the opposite result (Figure 5D). These results indicate that PYCR1 functioned as an oncogene in BCa cells.

### RAC3 Acted as an Oncogene by Activating PYCR1/JAK/STAT Signaling

**FIGURE 6:**
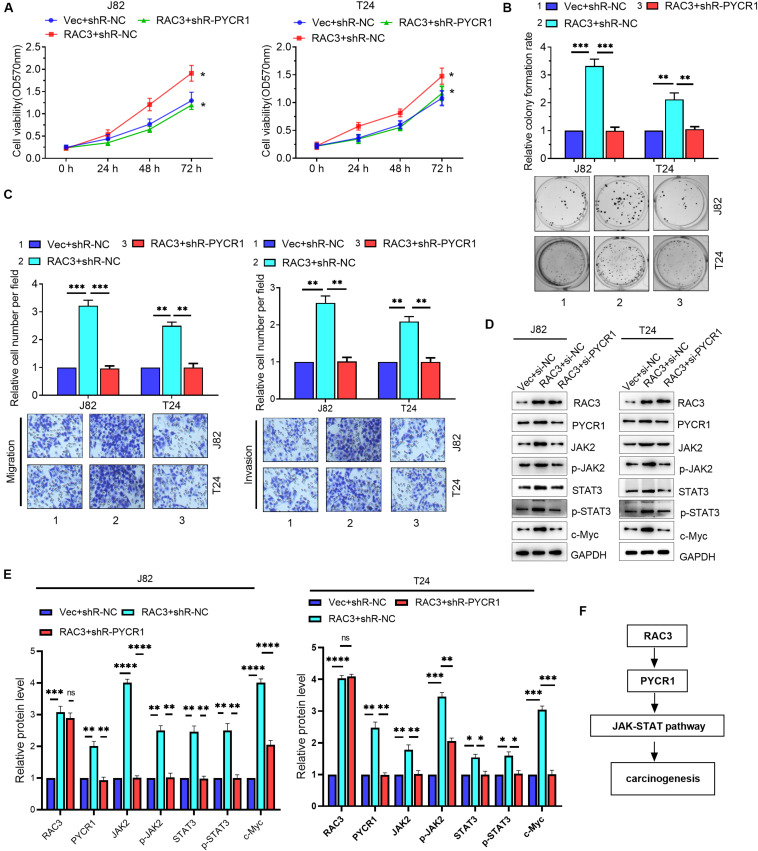
RAC3 promoted the proliferation, migration and invasion of BCa cells by activating PYCR1/JAK/STAT signaling. BCa cells were transfected with empty pcDNA3 vector (Vec) + si-NC, pcDNA3-RAC3 (RAC3) + si-NC, and pcDNA3-RAC3 (RAC3) + si-PYCR1. **(A–C)** MTT assay, colony formation assay, and Transwell assays were employed to detect the viability, proliferation, migration and invasion of BCa cells. **(D)** Western blot analysis was used to detect and quantify the protein levels of RAC3, PYCR1, JAK2, p-JAK2, STAT3, p-STAT3 and Bcl2. **(E)** Quantification of the protein expression of RAC3, PYCR1, JAK2, p-JAK2, STAT3, p-STAT3 and c-Myc were detected by WB. The proteins were normalizing to the Vec + shR control group. All experiments were replicated three times. **(F)** Proposed mechanism of RAC3 activated JAK/STAT3 signaling via the PYCR1 axis. ^∗^*p* < 0.05; ^∗∗^*p* < 0.01; ^∗∗∗^*p* < 0.001; ^****^p < 0.0001; ns - not significant.

Although we have shown that RAC3 could activate the JAK/STAT signaling, as well as, up-regulate the expression of PYCR1, RAC3 activation of JAK/STAT signaling via PYCR1 axis is unknown. We first investigated whether PYCR1 affected the JAK/STAT pathway in BCa cells. Western blot results revealed that the overexpression of PYCR1 markedly enhanced protein levels of JAK2, phosphorylated JAK2, STAT3, phosphorylated STAT3, and c-Myc, a STAT3-dependent gene (Supplementary Figure S2). On the contrary, this result was reversed by silencing PYCR1 (Supplementary Figure S2). We next ascertained whether RAC3 promoted the proliferation, migration, and invasion of BCa cells via PYCR1 axis. We found that the overexpression of RAC3 alone could significantly increase cell viabilities, colony number, as well as migration and invasion. However, co-transfection with PYCR1 knockdown plasmid significantly reversed these aforementioned cellular processes induced by RAC3 overexpression (Figures 6A–C). Furthermore, rescue experiments also demonstrated that silencing of PYCR1 could, in part, negatively affect the protein levels of JAK2, p-JAK2, STAT3, p-STAT3, and c-Myc induced by RAC3 (Figures 6D,E). Taken together, these results propose a novel mechanism that RAC3 acted as an oncogene to activate JAK/STAT signaling through the up-regulation of PYCR1 (Figure 6F).

## Discussion

Bladder Cancer, a malignancy with high recurrence and metastasis rate, is one of the most common cancer of the urinary system, and it prognosis has remained poor (Martinez Rodriguez et al., 2017). Therefore, it is important to investigate the key molecular mechanisms of dysregulated gene expression associated with BCa. In this study, we showed the effect of RAC3 on cell proliferation, migration, and invasion in BCa cells, as well as, the downstream regulatory mechanisms. Bioinformatics analysis revealed that RAC3 was up-regulated in BCa tissues and cells. RAC3 promoted cell proliferation, migration, and invasion by activating JAK/STAT signaling. Previous studies indicated that RAC3 promoted cell proliferation and cell aggressiveness in several cancers (Chan et al., 2005; Hwang et al., 2005; Engers et al., 2007; Gest et al., 2013; Liu et al., 2015; Zhang et al., 2017). Our results agree with this finding since we demonstrated that RAC3 promoted BCa cell proliferation, migration, and invasion.

The JAK-STAT signaling, a simple pathway beginning from the cell membrane to the nucleus, has received increasing attention recently. This pathway has been demonstrated to transduce extracellular signals into transcriptional programs that regulate cell growth and differentiation (Jenkins, 2014; O’Shea et al., 2015). JAK-STAT signaling can be activated by various upstream oncogenes that in turn activate STATs, which consequently regulates downstream expression of gene, such as c-Myc, Bcl2, TIMP-1, Pim-1, among others (O’Shea et al., 2015; Gündüz, 2019). Sustained activation of JAK/STAT signaling has been demonstrated in tumor initiation, progression, and cancer stem cell (CSC) maintenance in several human cancers. For instance, Zhu et al. (2019) reported that long non-coding RNA PART1 enhanced the proliferation, migration, and invasion via activation of JAK-STAT signaling in non-small-cell lung cancer cells. Dolatabadi et al. (2019) demonstrated that the activation of JAK-STAT signaling regulates the CSC properties in myxoid liposarcoma. Herein, we found that JAK/STAT signaling was activated by the overexpression of RAC3 in BCa cells. Nifuroxazide, a small inhibitor molecule of STAT3, was used to detect the effect on RAC3 over-expressing cells. Nifuroxazide, which has been previously reported as a small molecule inhibitor of JAK/STAT signaling (Ye et al., 2017; Luo et al., 2019), significantly inhibited the phosphorylation of JAK2 and STAT3 in RAC3-overexpressed BCa cells, which indicates that the activation of JAK/STAT signaling was partly attenuated.

In this study, we demonstrated that RAC3 could up-regulated PYCR1, which played an oncogenic role in the BCa cells. Most importantly, we demonstrated that RAC3 played an oncogenic role to activate JAK/STAT signaling via up-regulation of PYCR1 in the BCa cells. Furthermore, recent studies have also reported that PYCR1 is up-regulated in human cancers, and thus promote the progression of cancers including non-small-cell lung cancer (Wang et al., 2019), prostate cancer (Zeng et al., 2017), and hepatocellular cancer (Zhuang et al., 2019), among others. Moreover, Yan et al. (2019) and Gao et al. (2020) demonstrated that the knockdown of PYCR1 inhibits the activation of JAK/STAT signaling in lung adenocarcinoma and colorectal cancers, respectively. Additionally, another reports demonstrated that PYCR1 interference inhibited cell proliferation and enhanced cell apoptosis in hepatocellular carcinoma by repressing JNK/IRS1 pathway (Zhuang et al., 2019). In accord with the study of Yan et al. (2019) and Gao et al. (2020), our results also showed that PYCR1 could activate JAK/STAT signaling in the BCa cells. Therefore, we are highly convinced that the overexpression of RAC3 activated JAK/STAT signaling through PYCR1 axis.

In conclusion, the most important findings of this study is that RAC3 was markedly up-regulated in BCa tissues and cell lines, and that overexpression of RAC3 enhances the proliferation, migration, and invasion of BCa cells. RAC3 overexpression promoted cell proliferation, migration, and invasion, which was likely due to its role in activating JAK/STAT signaling mediated by PYCR1 in BCa.

## Data Availability Statement

All datasets presented in this study are included in the article/Supplementary Material.

## Author Contributions

CC and DS conceived, designed, and performed the study, analyzed the data, and contributed to the writing of the manuscript. YW and BL assisted with the data collection. All authors reviewed the manuscript.

## Conflict of Interest

The authors declare that the research was conducted in the absence of any commercial or financial relationships that could be construed as a potential conflict of interest.

## Supplementary Material

FIGURE S1 The mRNA and protein levels of RAC1 and RAC2 after silencing of RAC3 by shR-RAC3. **(A)** RT-qPCR detected the mRNA levels of RAC1 and RAC2 after silencing of RAC3 in BCa cells. **(B)** Western Blot detected the protein levels of RAC1, RAC2 and RAC3 after silencing of RAC3 in BCa cells. The proteins were normalizing to the vector and the shR control separately. All experiments were replicated three times. ^∗∗∗^*p* < 0.001; ns, not significant.

FIGURE S2 PYCR1 activated JAK/STAT signaling in BCa cells. **(A)** BCa cells were transfected with empty pcDNA3 vector (Vec), pcDNA3-PYCR1 (PYCR1), shR-NC, and shR-PYCR1, western blot was used to detect the protein levels of PYCR1, JAK2, p-JAK2, STAT3, p-STAT3 and c-Myc. **(B,C)** Quantification of the protein expression of PYCR1, JAK2, p-JAK2, STAT3, p-STAT3 and c-Myc were detected by WB. The proteins were normalizing to the vector and the shR control separately. ^∗∗^*p* < 0.01; ^∗∗∗^*p* < 0.001; ^****^*p* < 0.0001.

DATA SHEET S1 A full scan of the entire original gel(s).
