# Supplementary material for: RAC3 Promotes Proliferation, Migration and Invasion via PYCR1/JAK/STAT Signaling in Bladder Cancer
Source: Front Mol Biosci. 2020 Aug 31;7:218. doi: 10.3389/fmolb.2020.00218 (PMC7488983; doi:10.3389/fmolb.2020.00218)
Supplement: FIGURE S1 — The mRNA and protein levels of RAC1 and RAC2 after silencing of RAC3 by shR-RAC3. (A) RT-qPCR detected the mRNA levels of RAC1 and RAC2 after silencing of RAC3 in BCa cells. (B) Western Blot detected the protein levels of RAC1, RAC2 and RAC3 after silencing of RAC3 in BCa cells. The proteins were normalizing to the vector and the shR control separately. All experiments were replicated three times. ∗∗∗p < 0.001; ns, not significant. [file Image_1.pdf]

Supplementary Figures

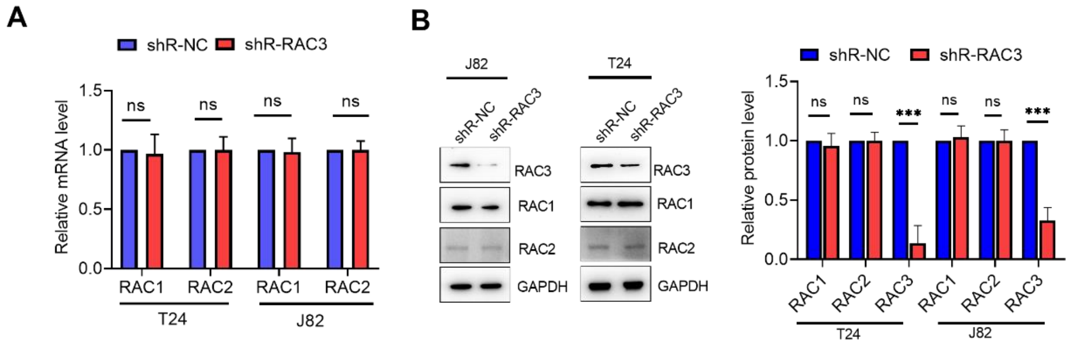

Figure S1. The mRNA and protein levels of RAC1 and RAC2 after silencing of RAC3 by shR-RAC3. (A) RT-qPCR detected the mRNA levels of RAC1 and RAC2 after silencing of RAC3 in BCa cells. (B) Western Blot detected the protein levels of RAC1, RAC2 and RAC3 after silencing of RAC3 in BCa cells. The proteins were normalizing to the vector and the shR control separately. All experiments were replicated three times. ***$p < 0.001$; ns, not significant.

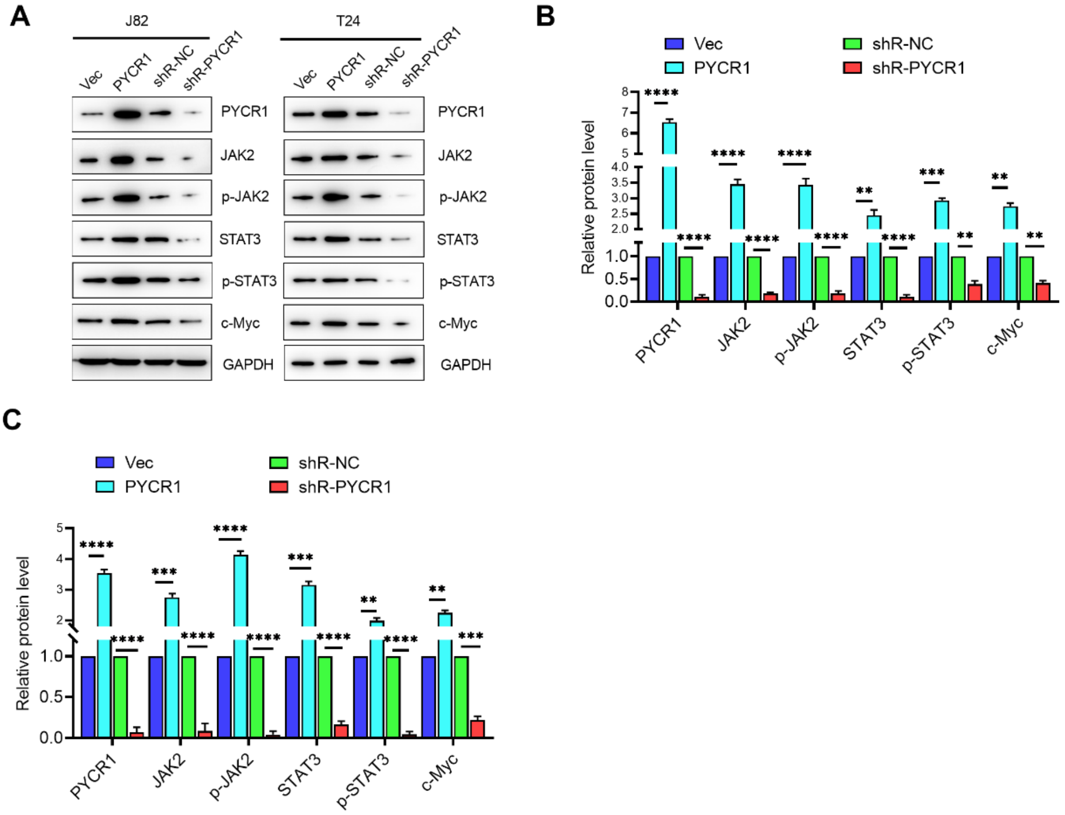

Figure S2. PYCR1 activated JAK/STAT signaling in BCa cells. (A) BCa cells were transfected with empty pcDNA3 vector (Vec), pcDNA3-PYCR1 (PYCR1), shR-NC, and shR-PYCR1, western blot was used to detect the protein levels of PYCR1, JAK2, p-JAK2, STAT3, p-STAT3 and c-Myc. (B and C) Quantification of the protein expression of PYCR1, JAK2, p-JAK2, STAT3, p-STAT3 and c-Myc were detected by WB. The proteins were normalizing to the vector and the shR control separately. **p<0.01; ***p<0.001; ****p<0.0001
