# Supplementary figures and images for: RAC3 Promotes Proliferation, Migration and Invasion via PYCR1/JAK/STAT Signaling in Bladder Cancer
Source: Front Mol Biosci. 2020 Aug 31;7:218. doi: 10.3389/fmolb.2020.00218 (PMC7488983; doi:10.3389/fmolb.2020.00218)

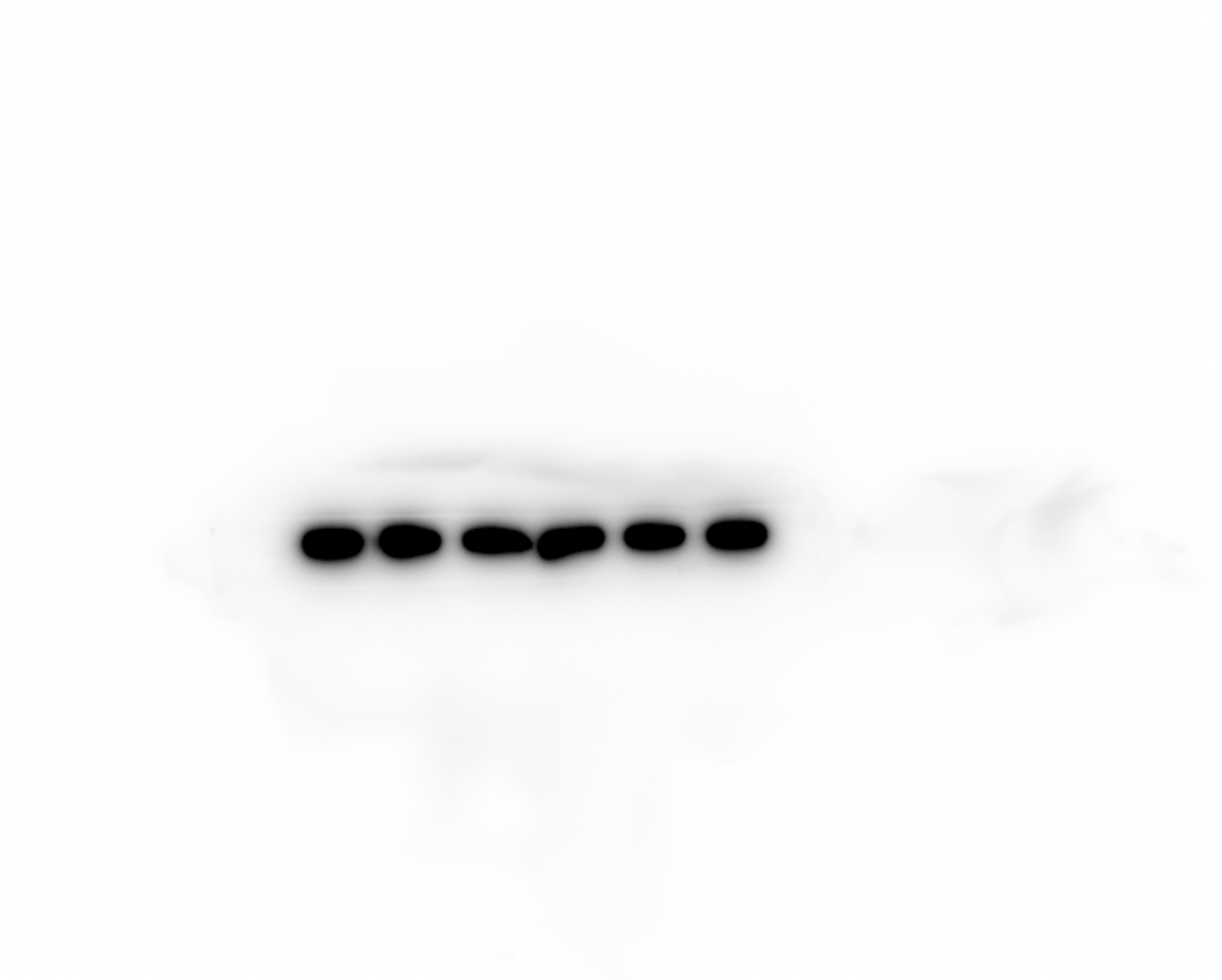

Supplement: DATA SHEET S1 — A full scan of the entire original gel(s). [file Data_Sheet_1.zip › original image files/Figure 1E/GAPDH.Tif]

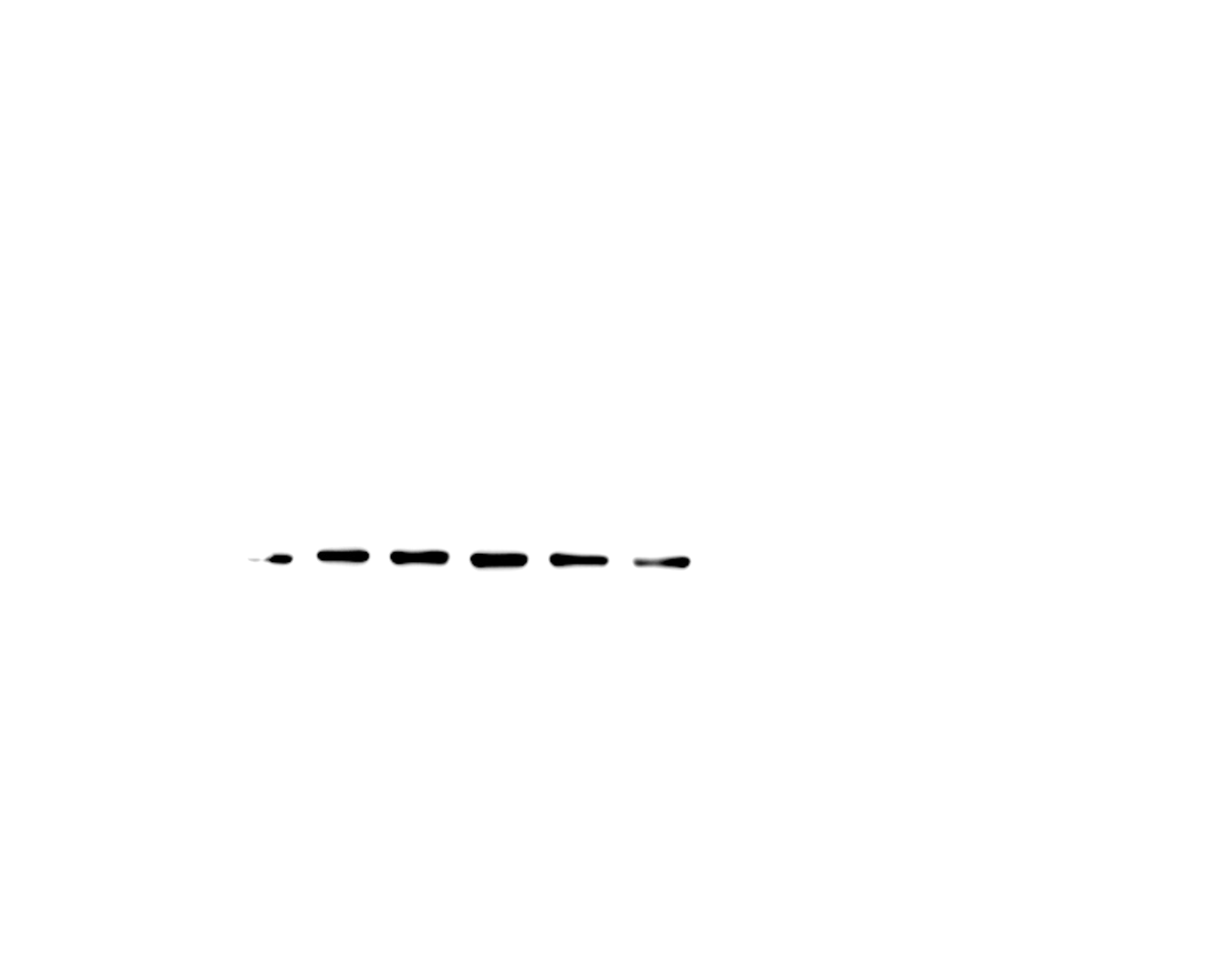

Supplement: DATA SHEET S1 — A full scan of the entire original gel(s). [file Data_Sheet_1.zip › original image files/Figure 1E/RAC3-active .Tif]

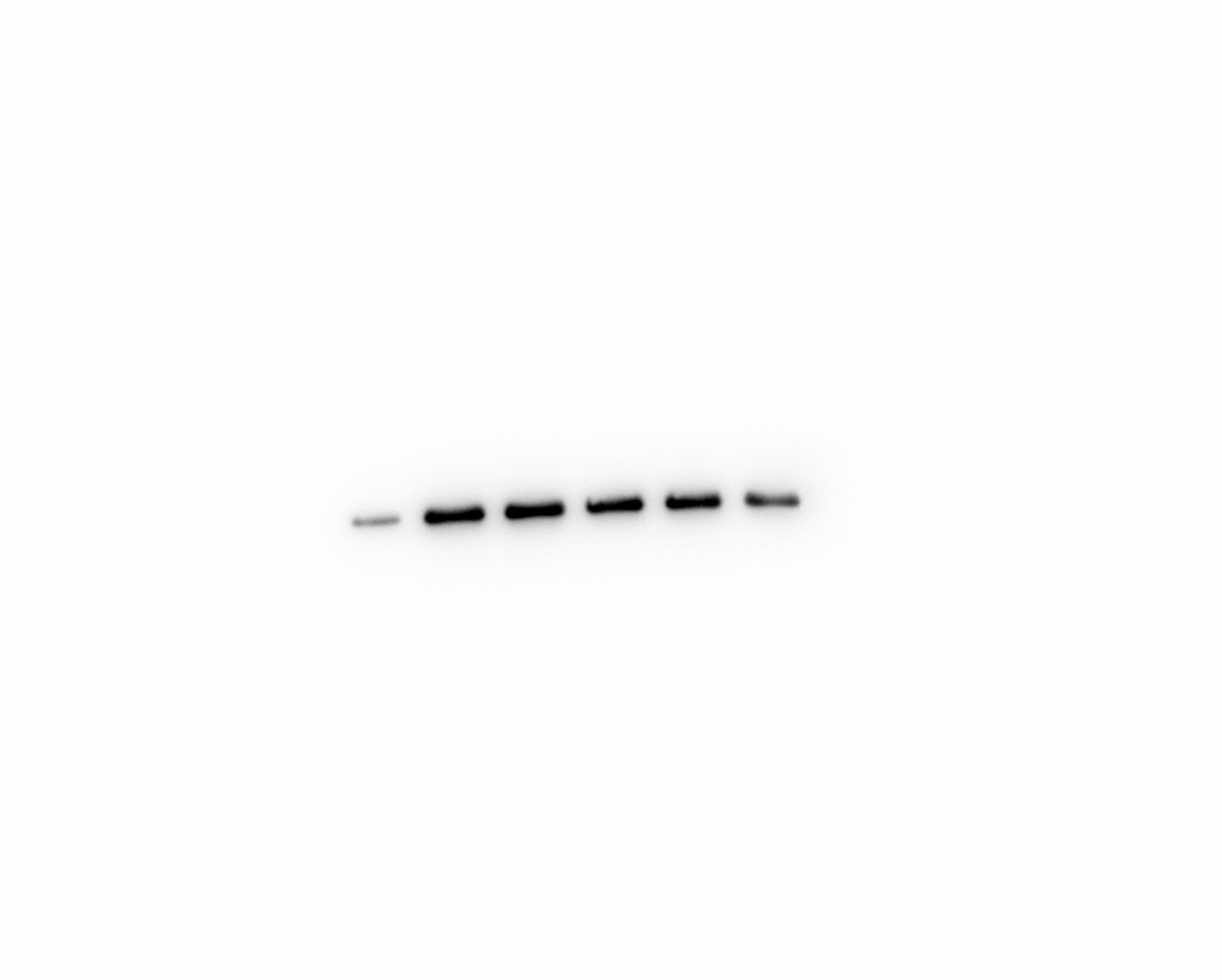

Supplement: DATA SHEET S1 — A full scan of the entire original gel(s). [file Data_Sheet_1.zip › original image files/Figure 1E/RAC3.Tif]

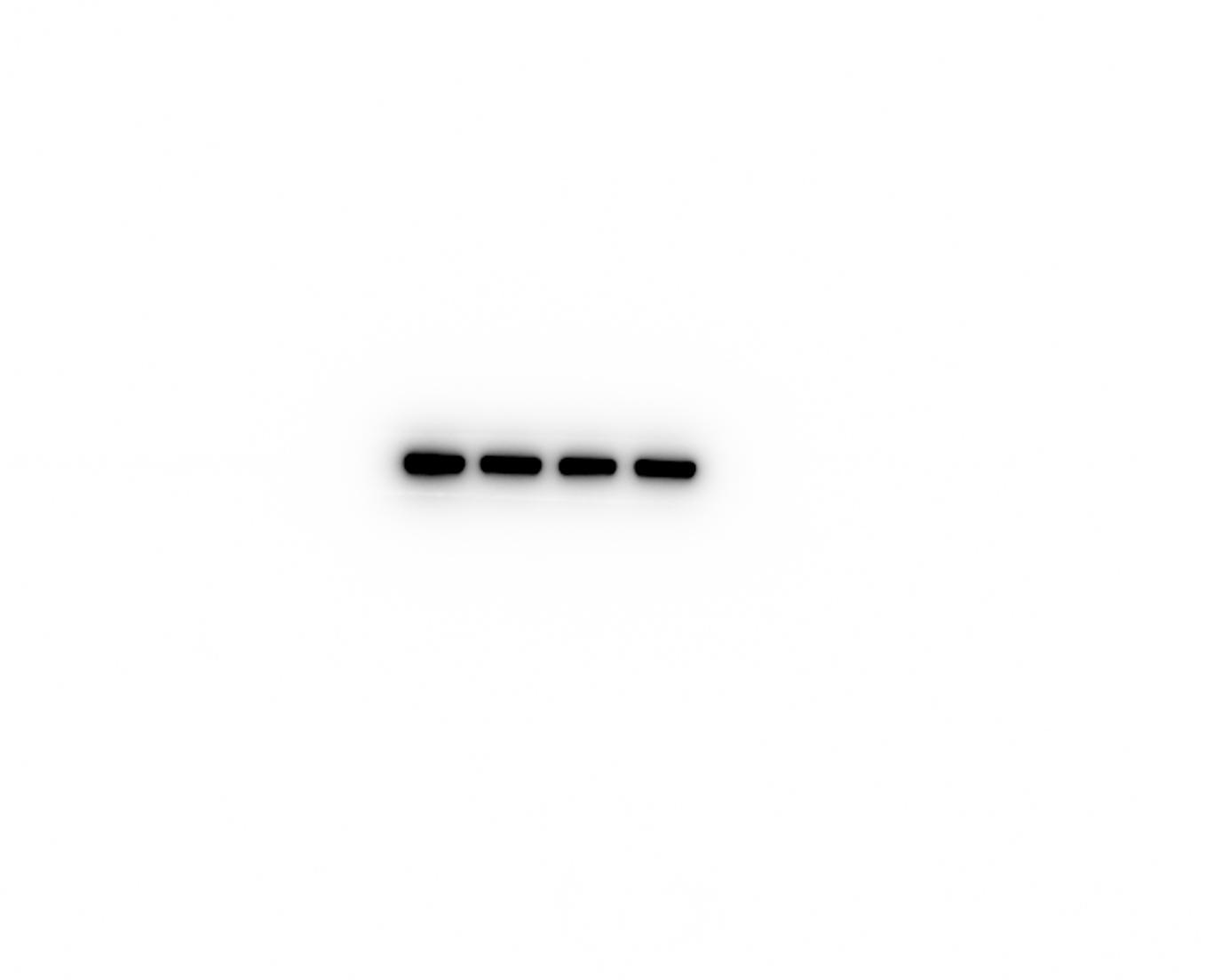

Supplement: DATA SHEET S1 — A full scan of the entire original gel(s). [file Data_Sheet_1.zip › original image files/Figure 3A/J82/GAPDH.Tif]

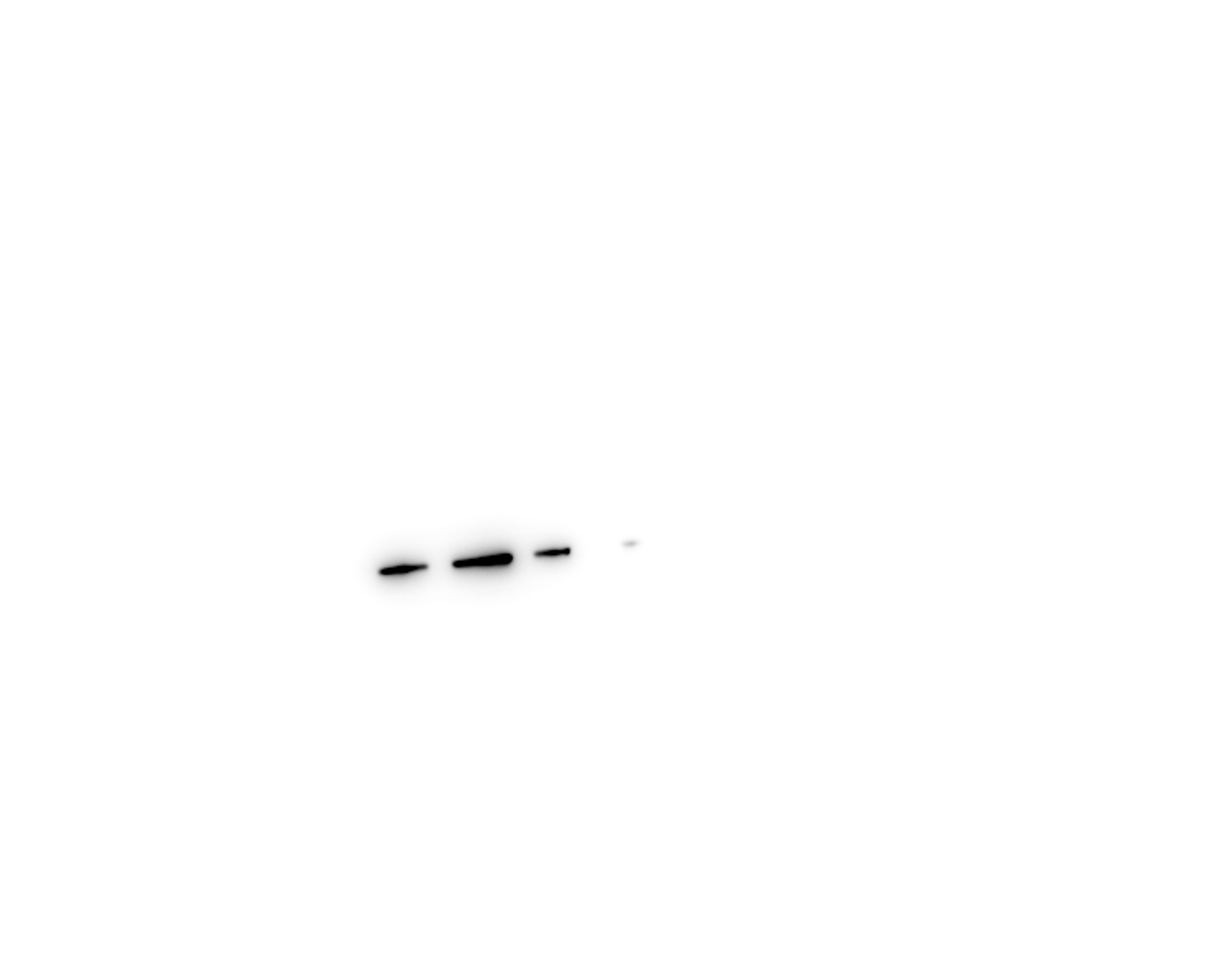

Supplement: DATA SHEET S1 — A full scan of the entire original gel(s). [file Data_Sheet_1.zip › original image files/Figure 3A/J82/JAK2.Tif]

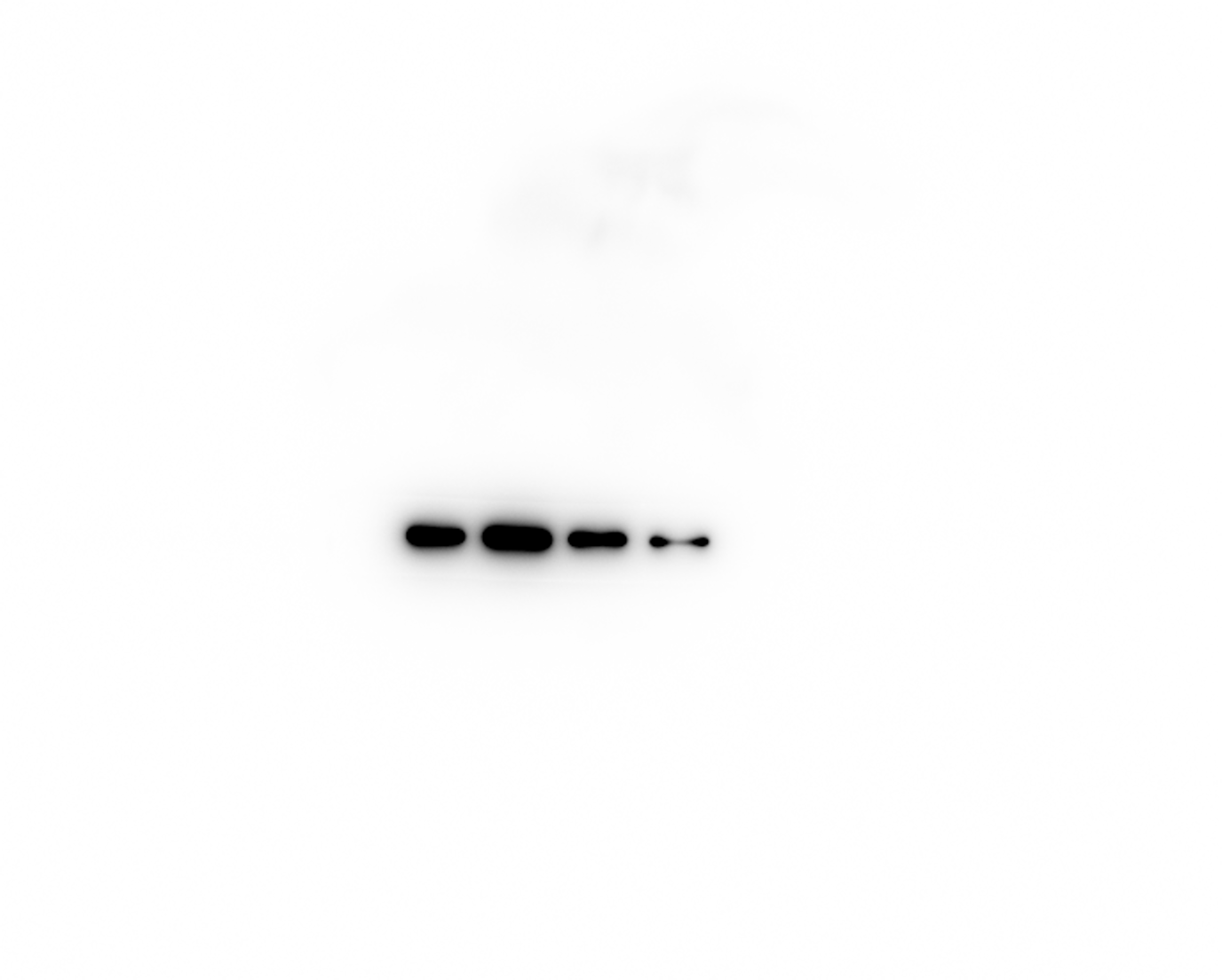

Supplement: DATA SHEET S1 — A full scan of the entire original gel(s). [file Data_Sheet_1.zip › original image files/Figure 3A/J82/RAC3.Tif]

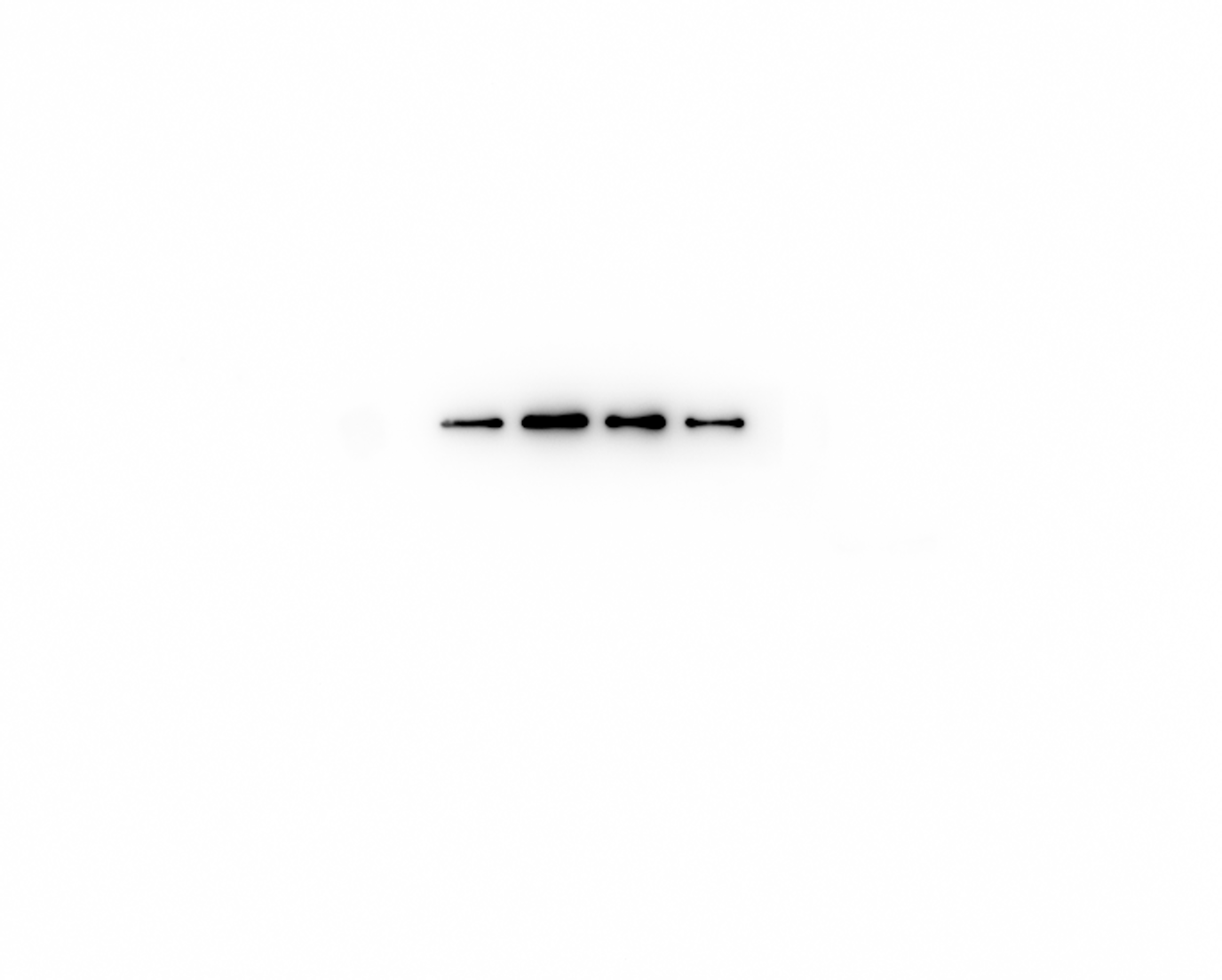

Supplement: DATA SHEET S1 — A full scan of the entire original gel(s). [file Data_Sheet_1.zip › original image files/Figure 3A/J82/STAT3.Tif]

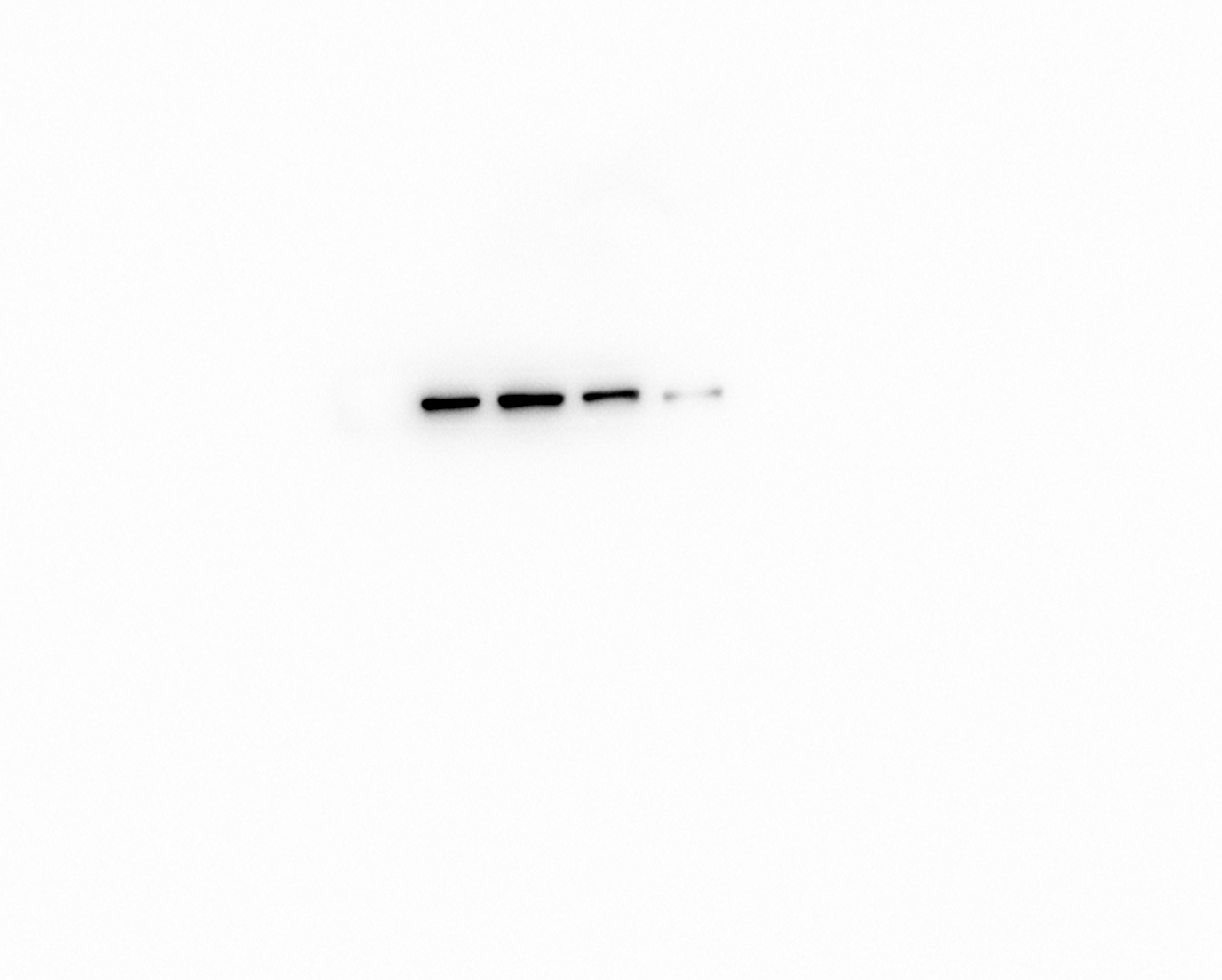

Supplement: DATA SHEET S1 — A full scan of the entire original gel(s). [file Data_Sheet_1.zip › original image files/Figure 3A/J82/c-MYC.Tif]

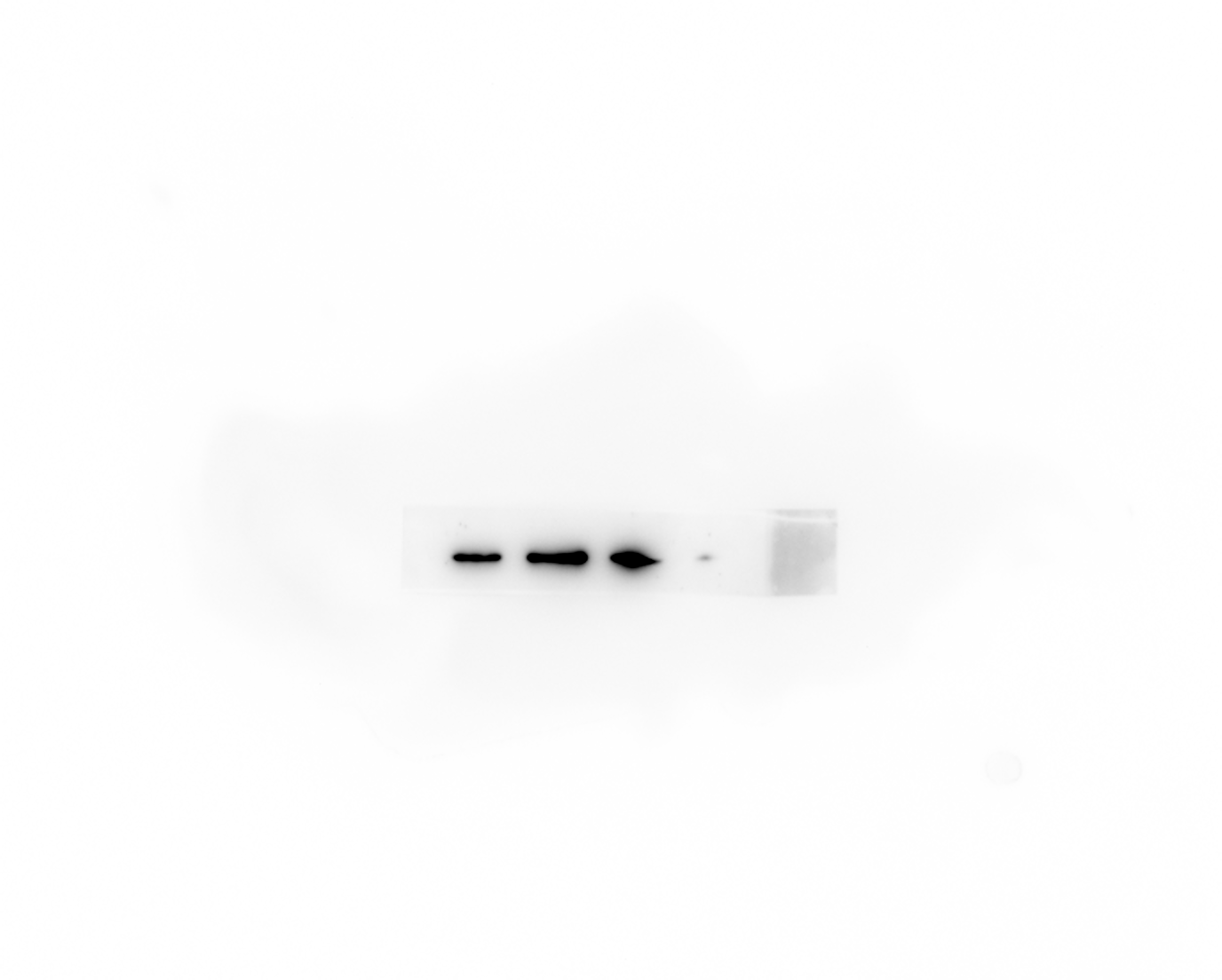

Supplement: DATA SHEET S1 — A full scan of the entire original gel(s). [file Data_Sheet_1.zip › original image files/Figure 3A/J82/pJAK2.Tif]

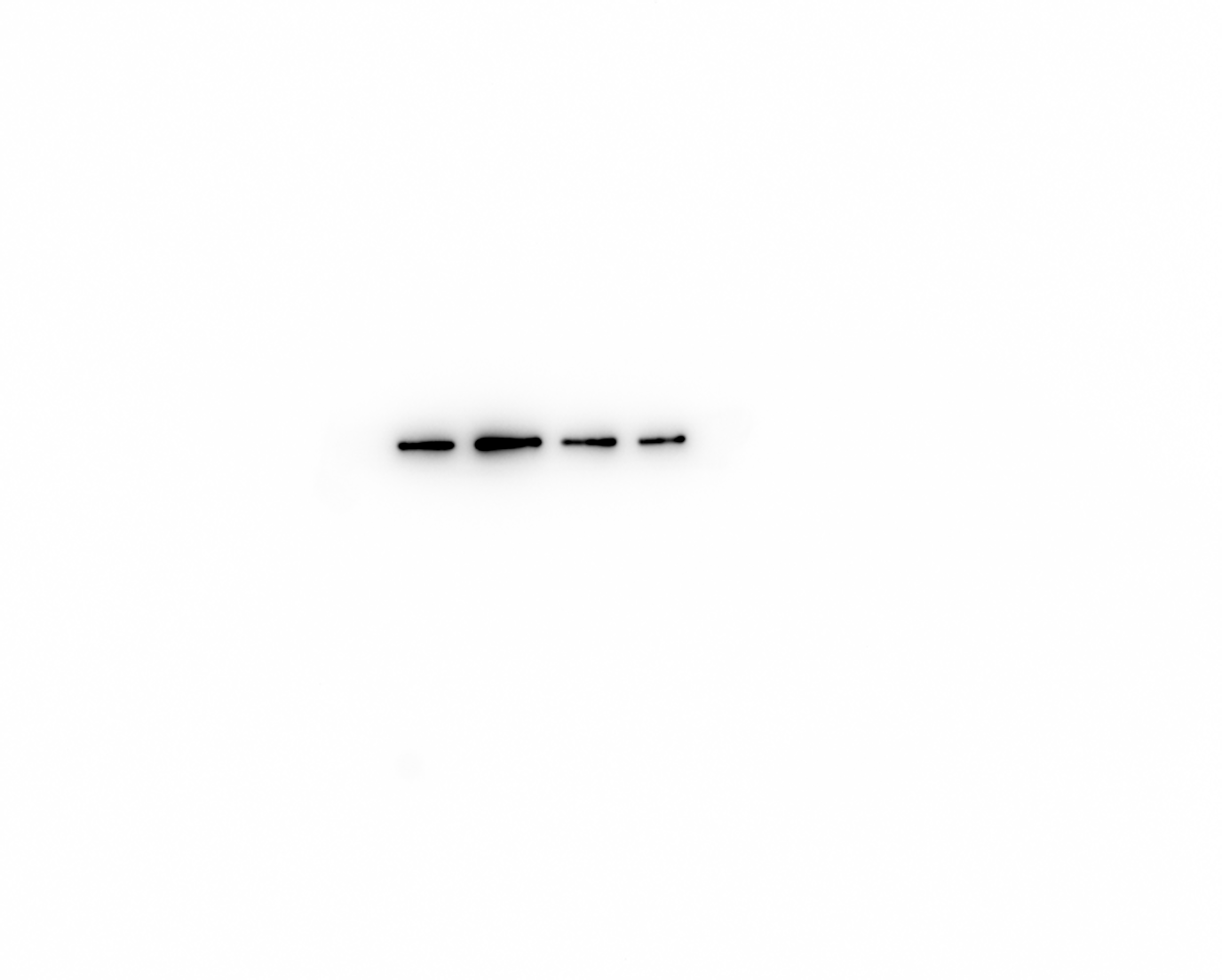

Supplement: DATA SHEET S1 — A full scan of the entire original gel(s). [file Data_Sheet_1.zip › original image files/Figure 3A/J82/pSTAT3.Tif]

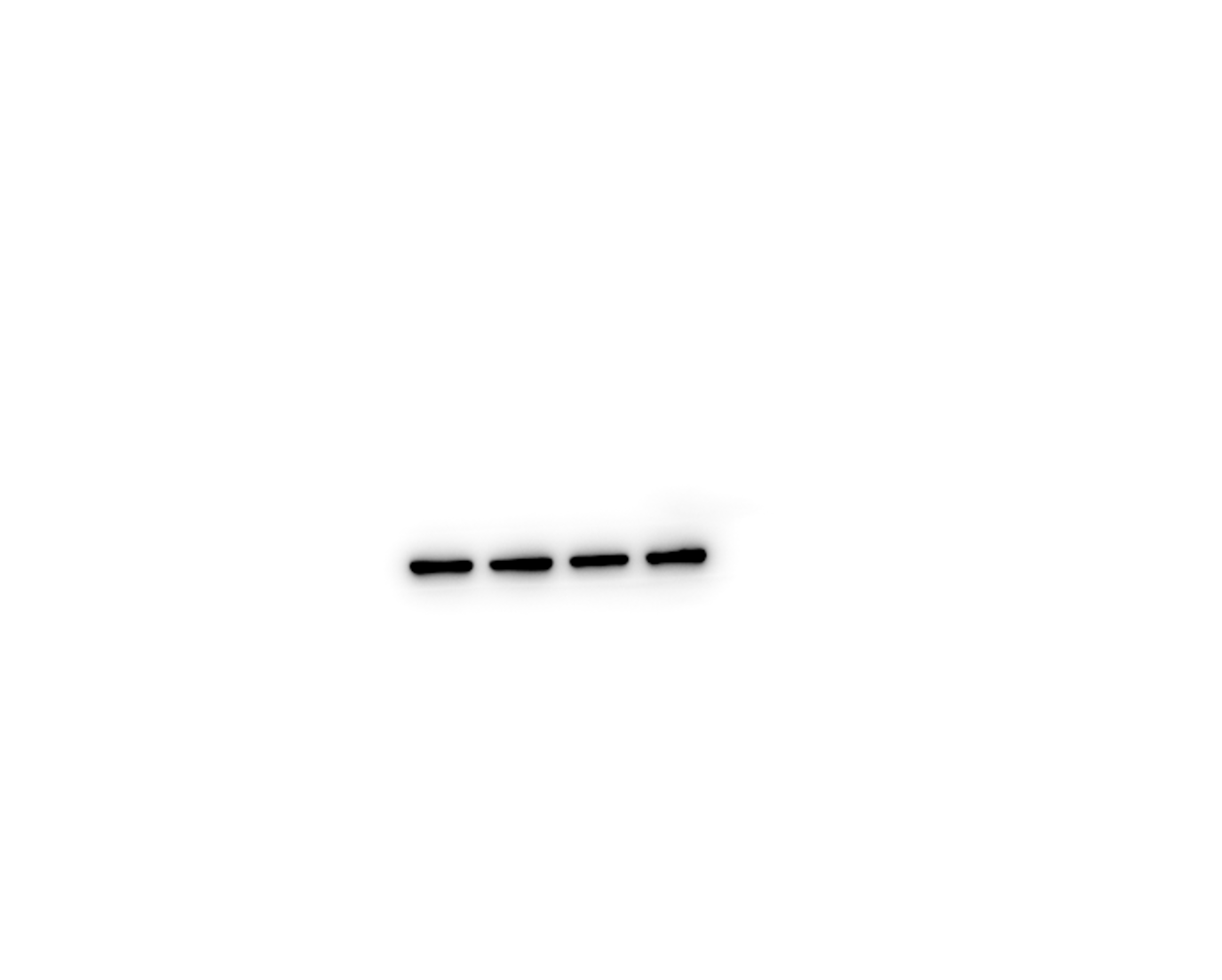

Supplement: DATA SHEET S1 — A full scan of the entire original gel(s). [file Data_Sheet_1.zip › original image files/Figure 3A/T24/GAPDH.Tif]

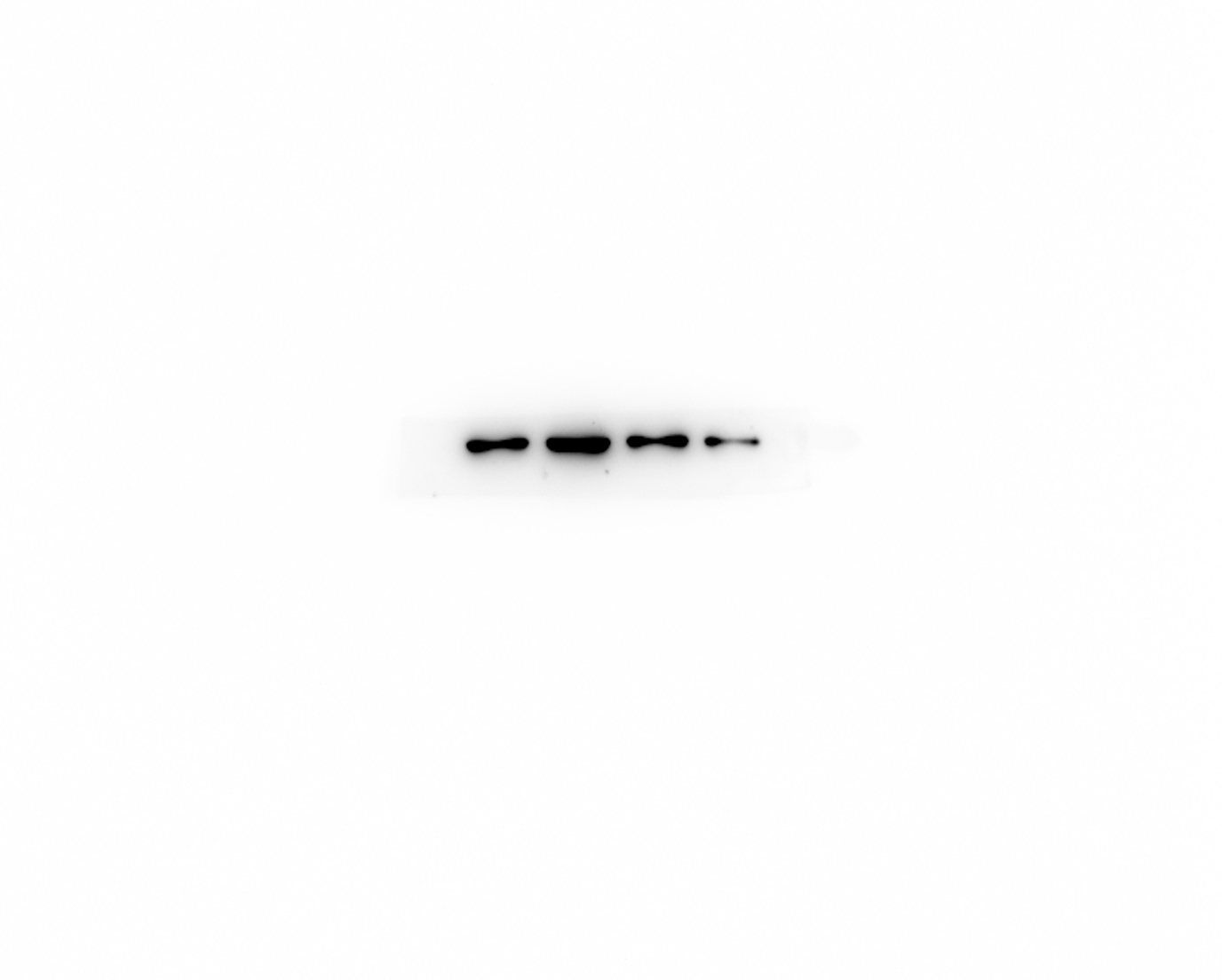

Supplement: DATA SHEET S1 — A full scan of the entire original gel(s). [file Data_Sheet_1.zip › original image files/Figure 3A/T24/JAK2.Tif]

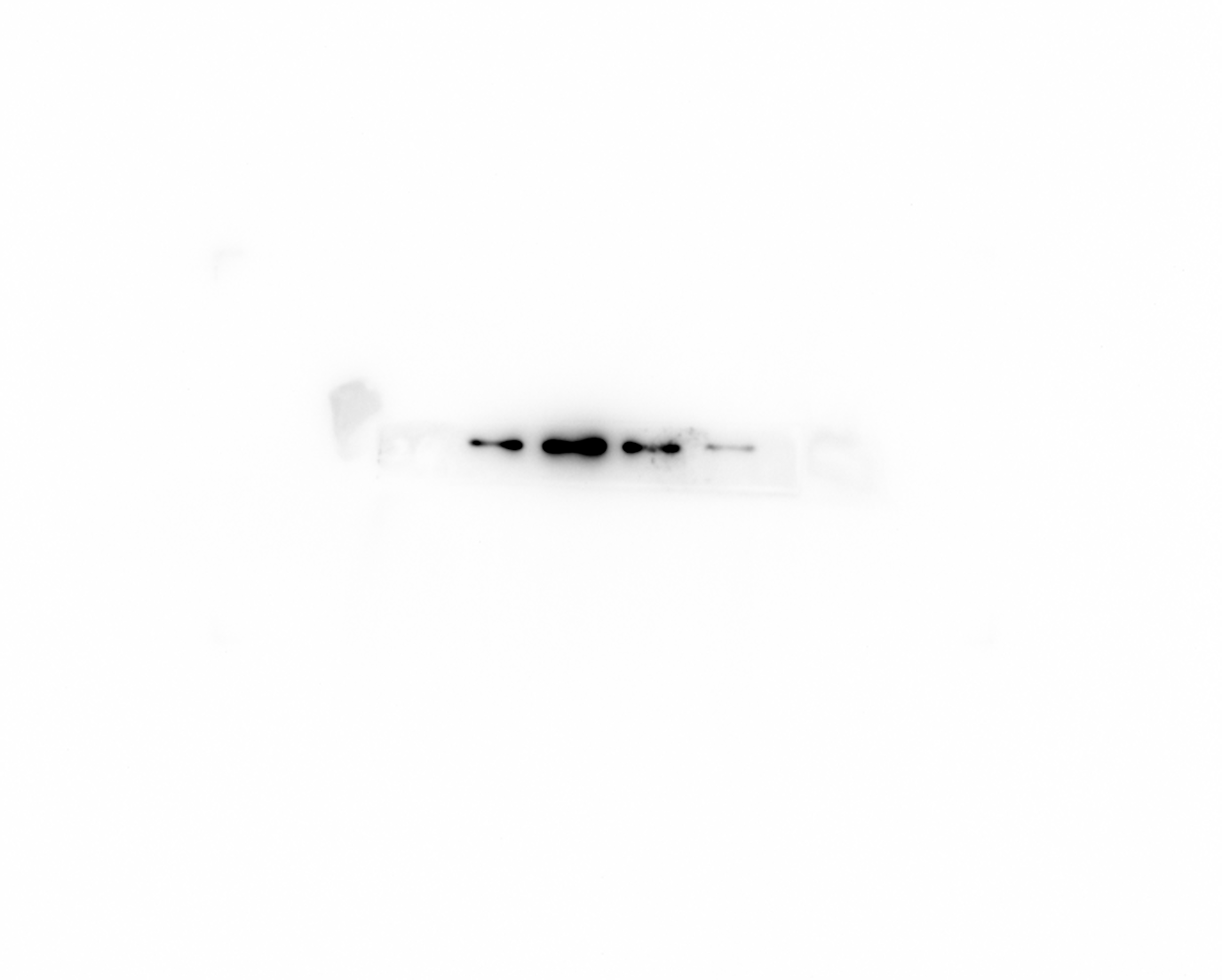

Supplement: DATA SHEET S1 — A full scan of the entire original gel(s). [file Data_Sheet_1.zip › original image files/Figure 3A/T24/RAC3.Tif]

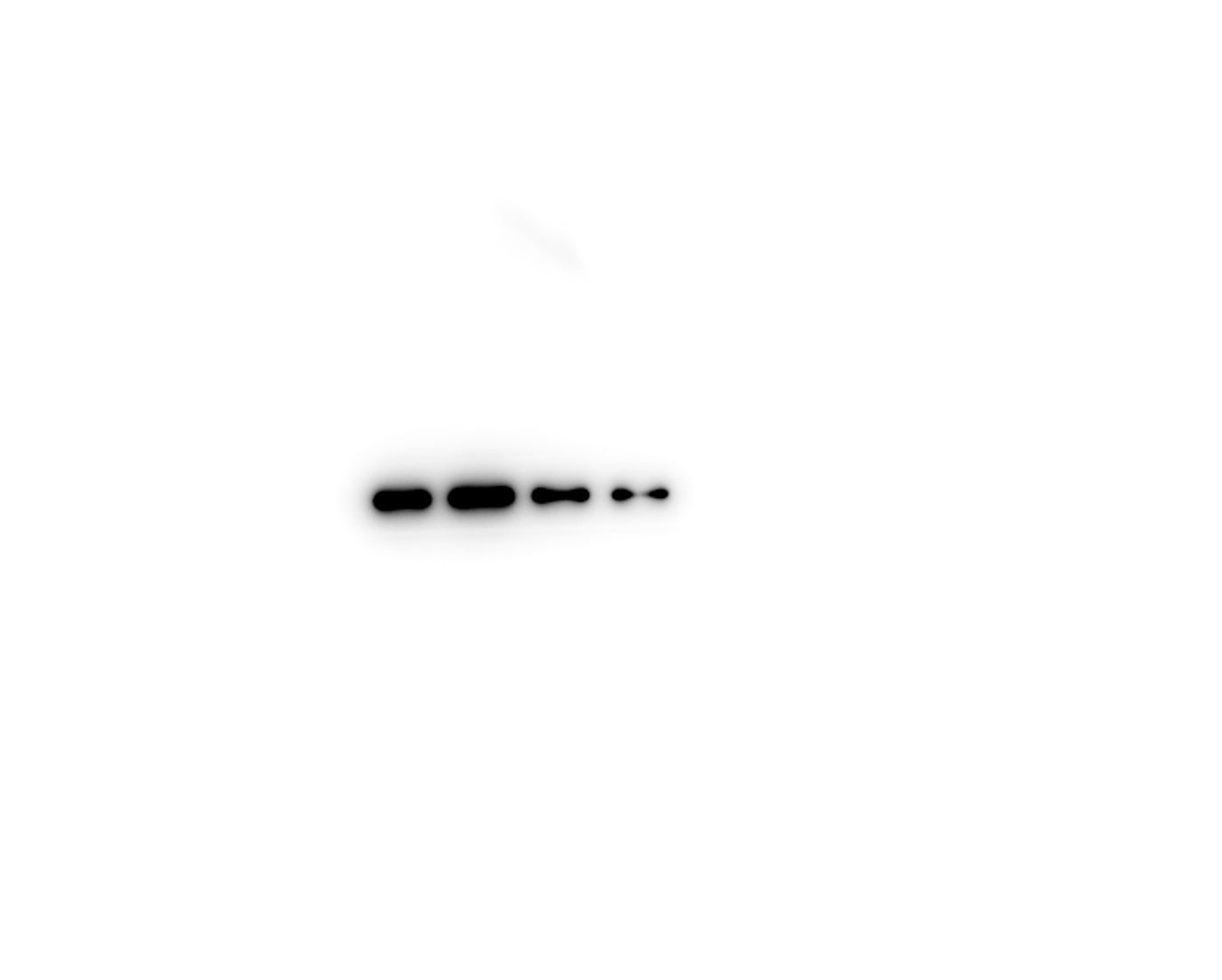

Supplement: DATA SHEET S1 — A full scan of the entire original gel(s). [file Data_Sheet_1.zip › original image files/Figure 3A/T24/STAT3.Tif]

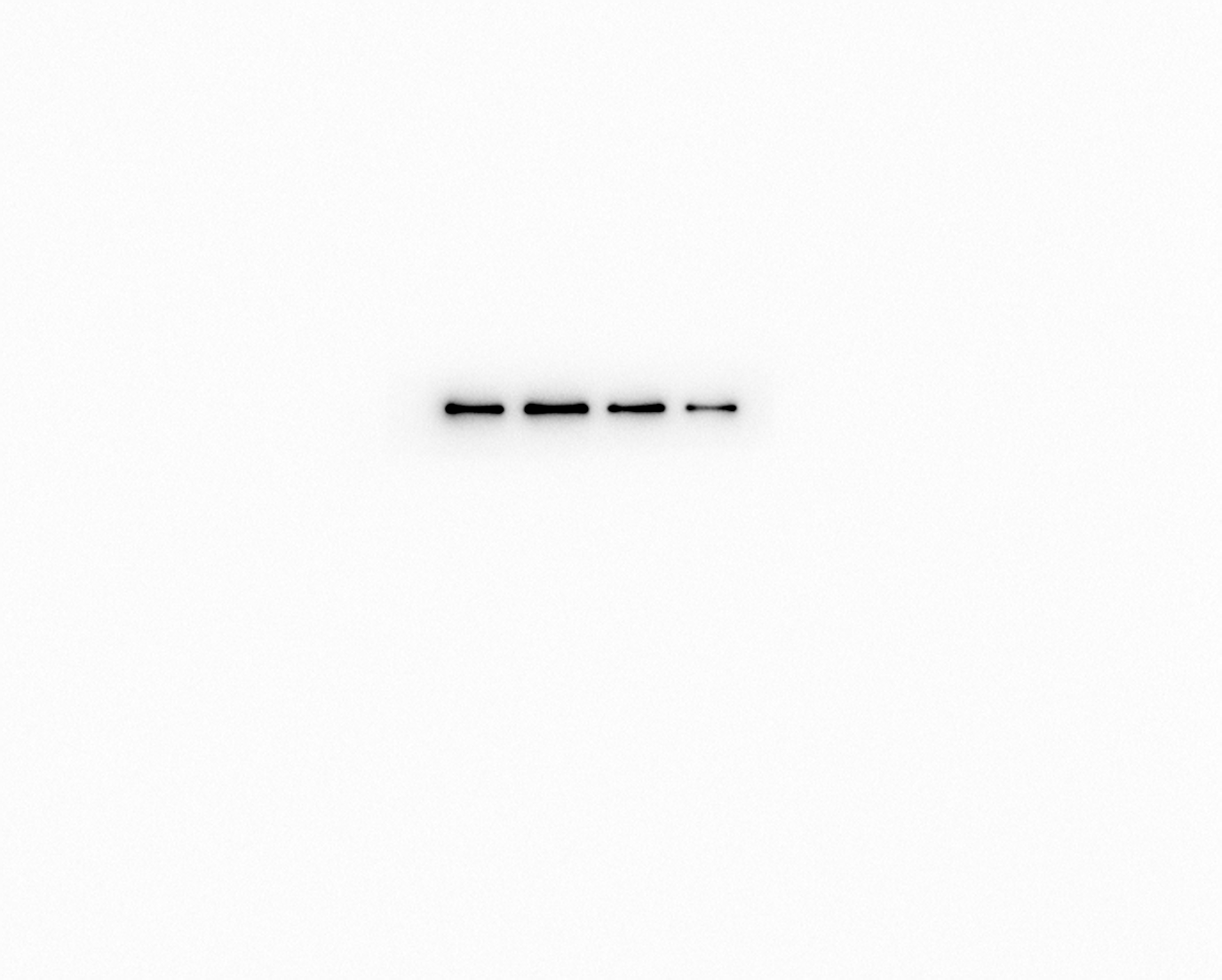

Supplement: DATA SHEET S1 — A full scan of the entire original gel(s). [file Data_Sheet_1.zip › original image files/Figure 3A/T24/c-myc.Tif]

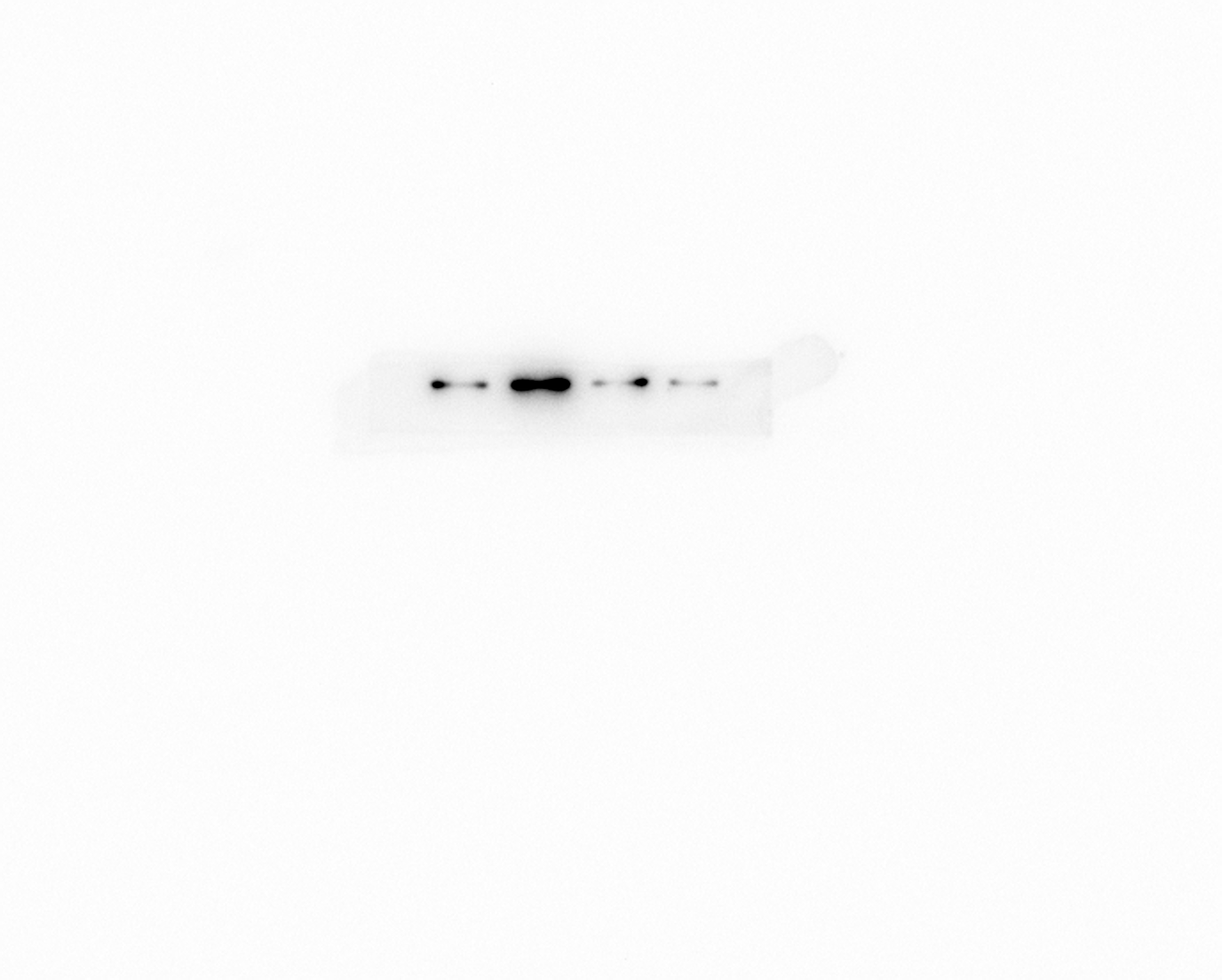

Supplement: DATA SHEET S1 — A full scan of the entire original gel(s). [file Data_Sheet_1.zip › original image files/Figure 3A/T24/pJAK2.Tif]

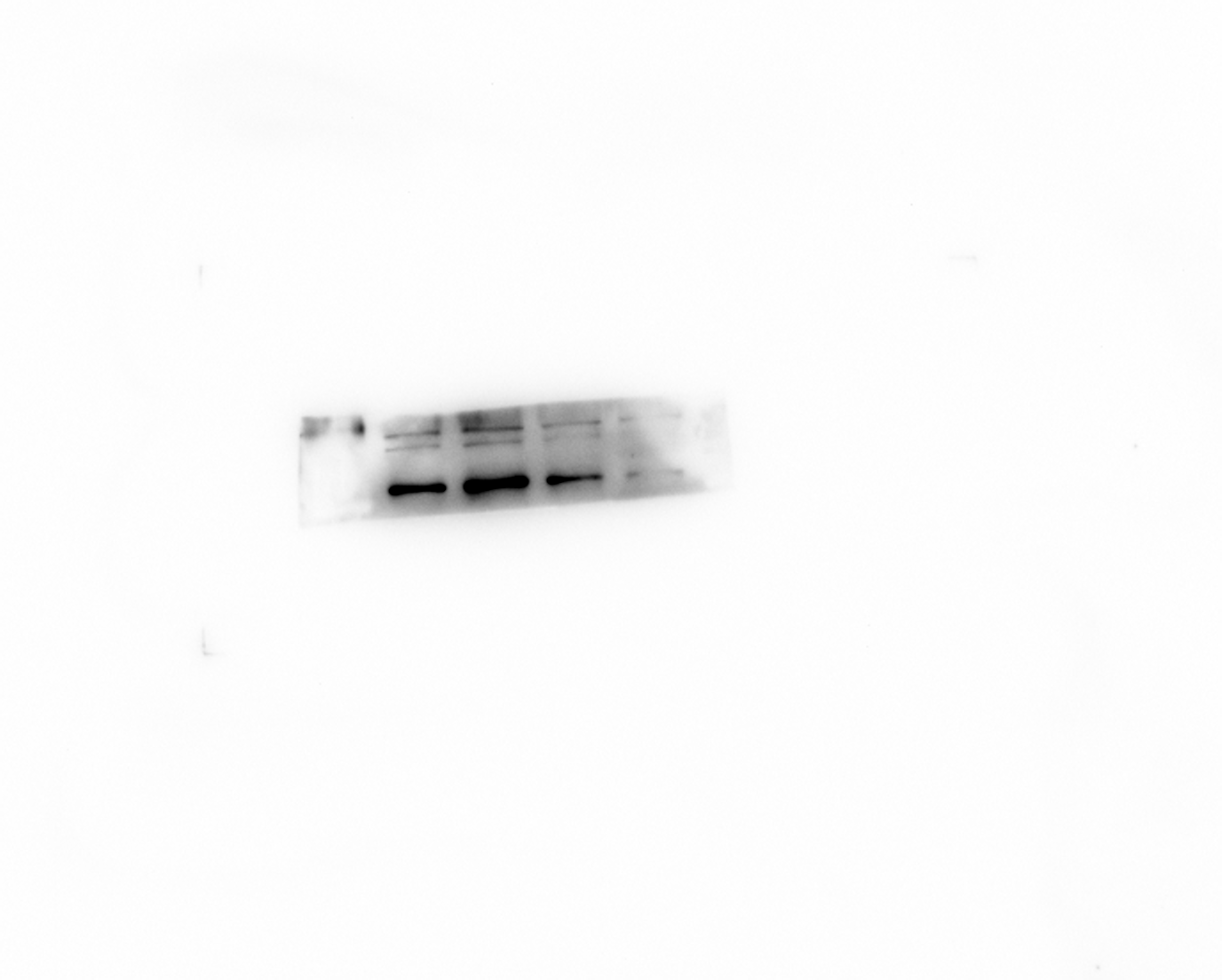

Supplement: DATA SHEET S1 — A full scan of the entire original gel(s). [file Data_Sheet_1.zip › original image files/Figure 3A/T24/pSTAT3.Tif]

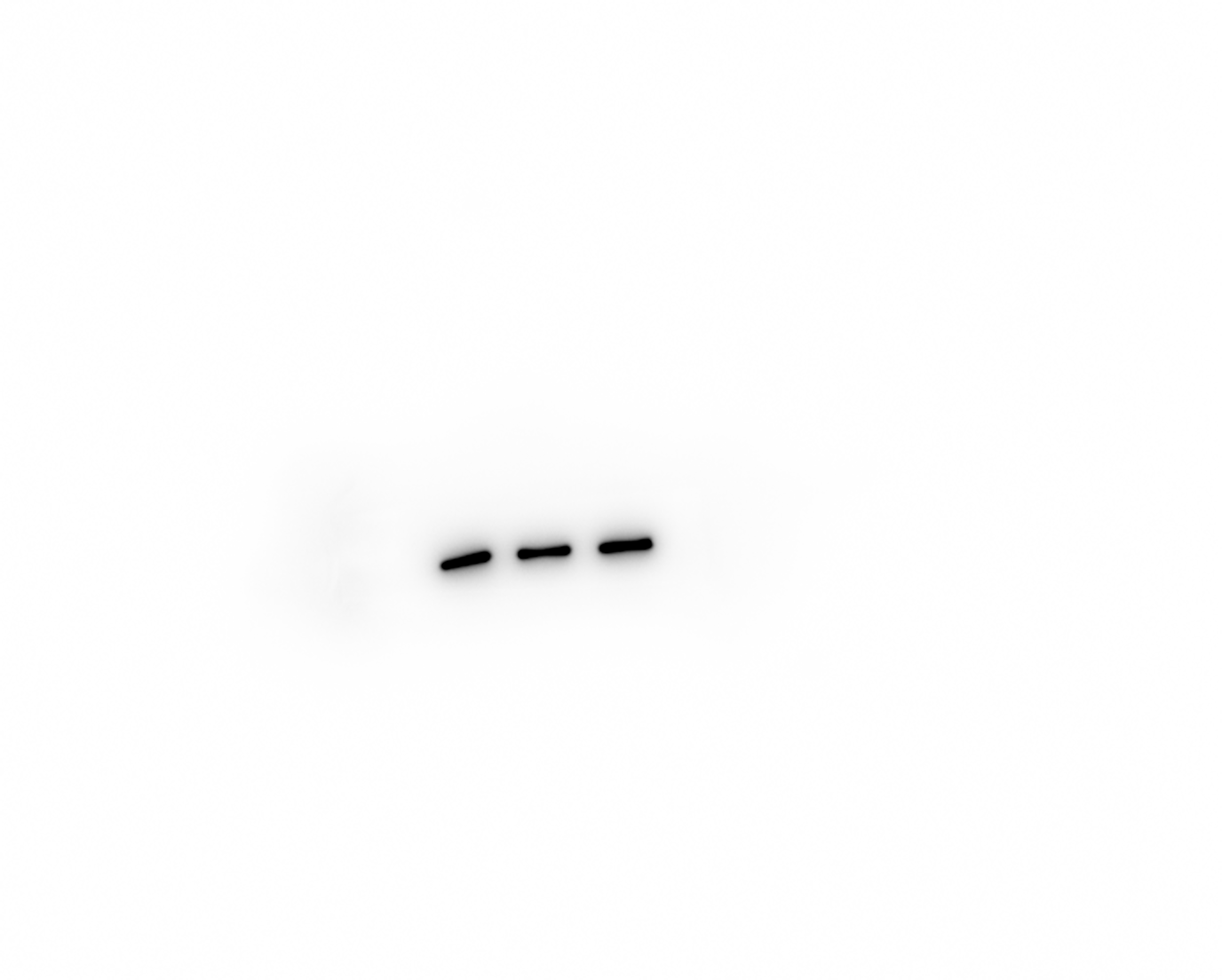

Supplement: DATA SHEET S1 — A full scan of the entire original gel(s). [file Data_Sheet_1.zip › original image files/Figure 3C/J82/GAPDH.Tif]

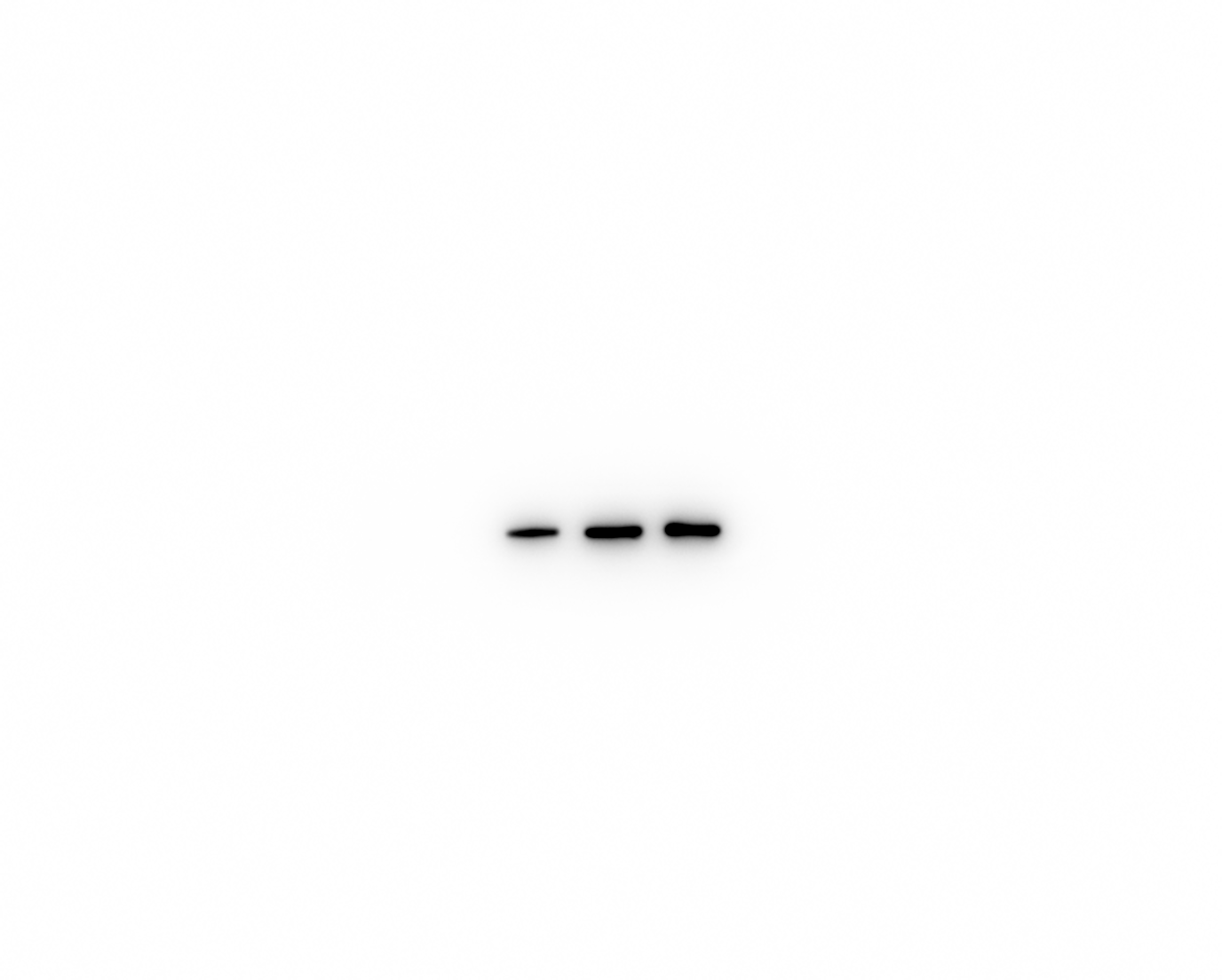

Supplement: DATA SHEET S1 — A full scan of the entire original gel(s). [file Data_Sheet_1.zip › original image files/Figure 3C/J82/JAK2.Tif]

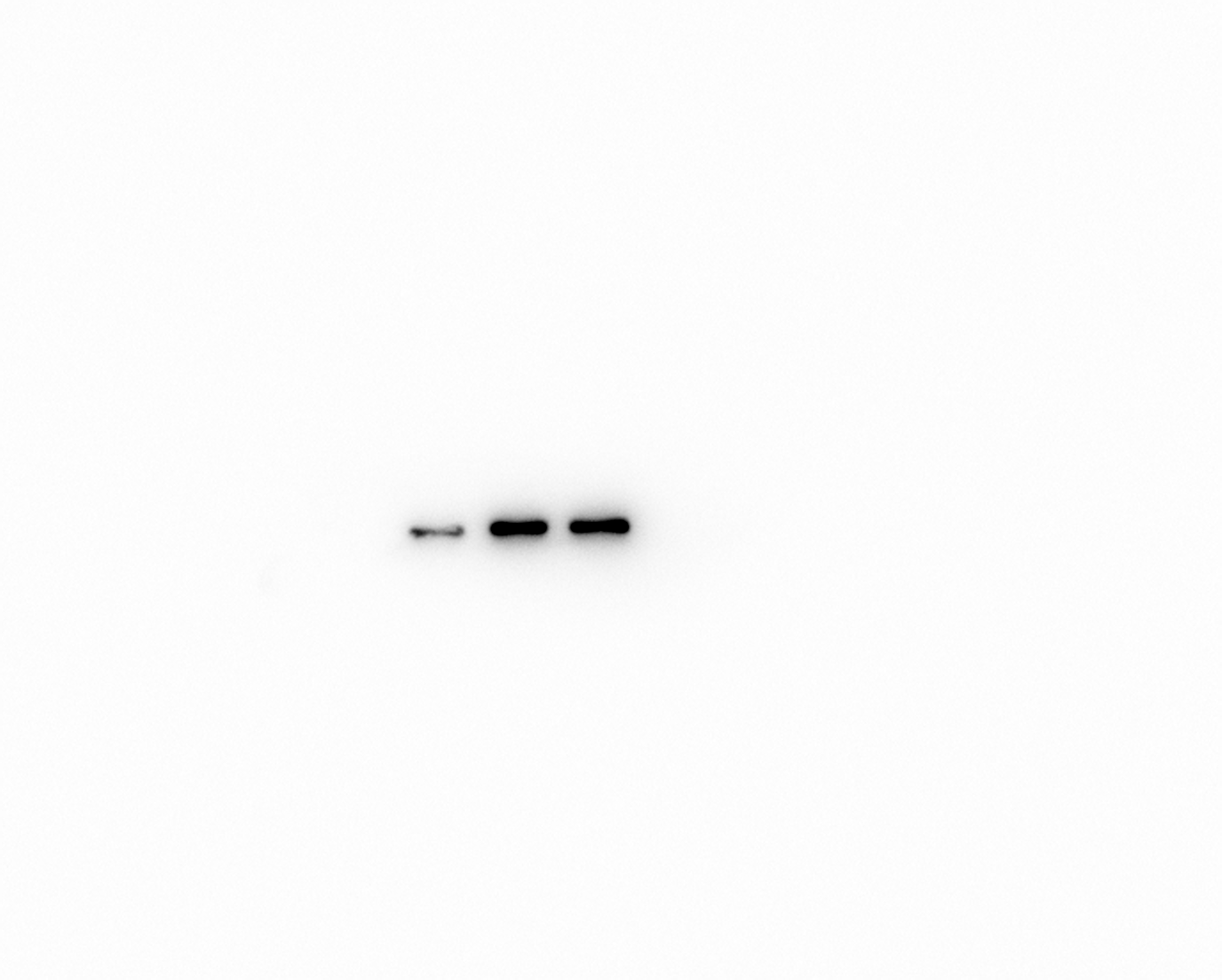

Supplement: DATA SHEET S1 — A full scan of the entire original gel(s). [file Data_Sheet_1.zip › original image files/Figure 3C/J82/RAC3.Tif]

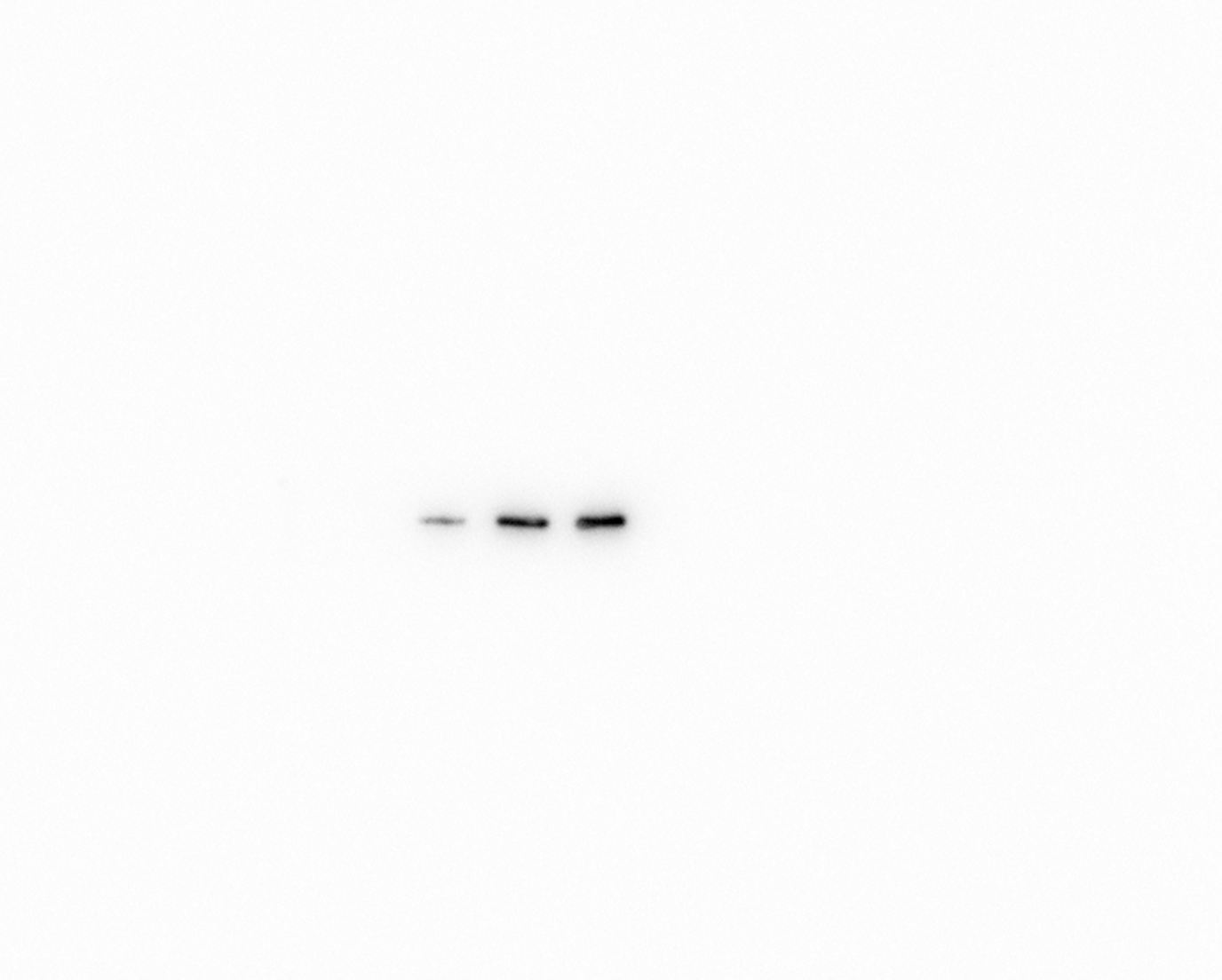

Supplement: DATA SHEET S1 — A full scan of the entire original gel(s). [file Data_Sheet_1.zip › original image files/Figure 3C/J82/STAT3.Tif]

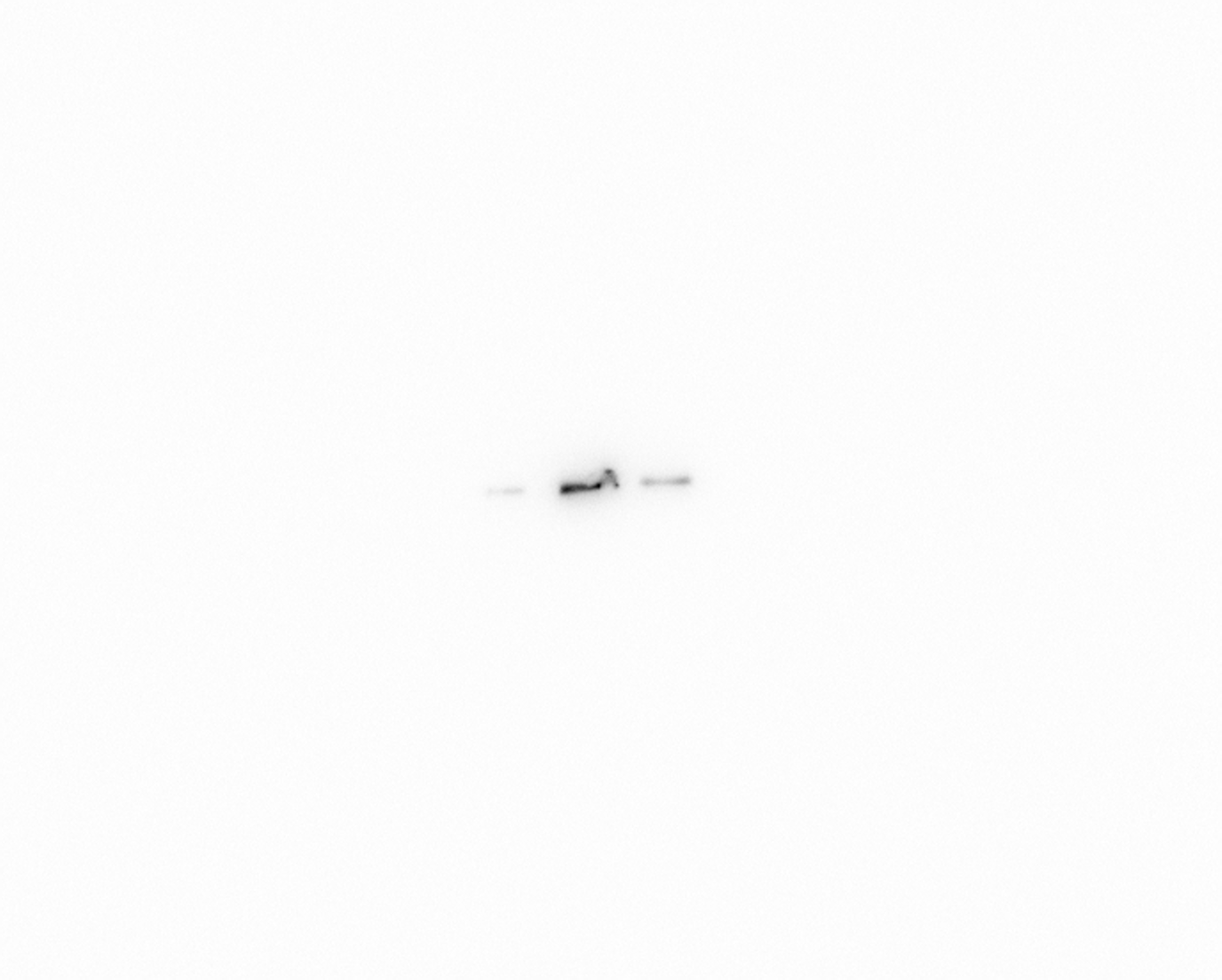

Supplement: DATA SHEET S1 — A full scan of the entire original gel(s). [file Data_Sheet_1.zip › original image files/Figure 3C/J82/c-MYC.Tif]

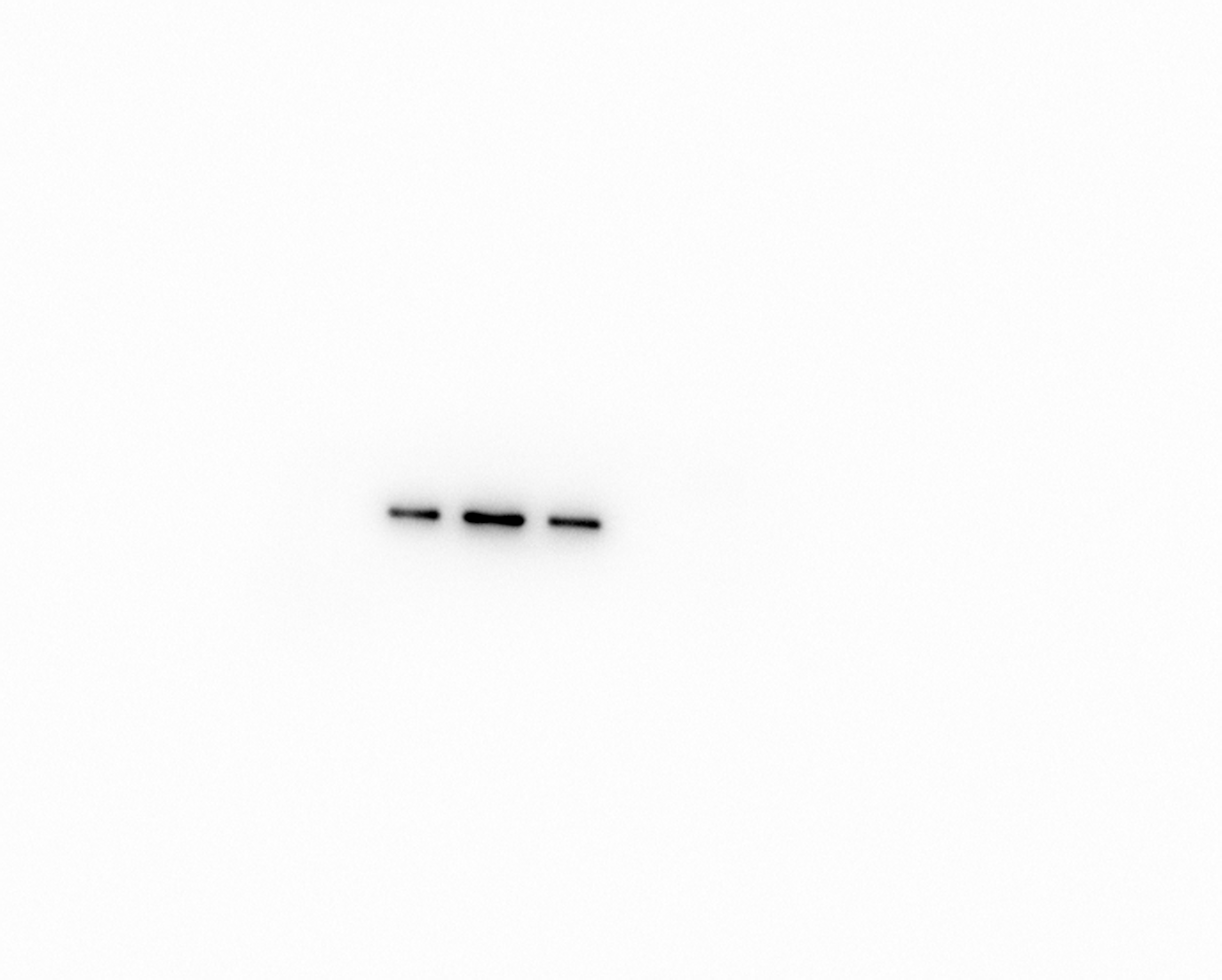

Supplement: DATA SHEET S1 — A full scan of the entire original gel(s). [file Data_Sheet_1.zip › original image files/Figure 3C/J82/pJAK2.Tif]

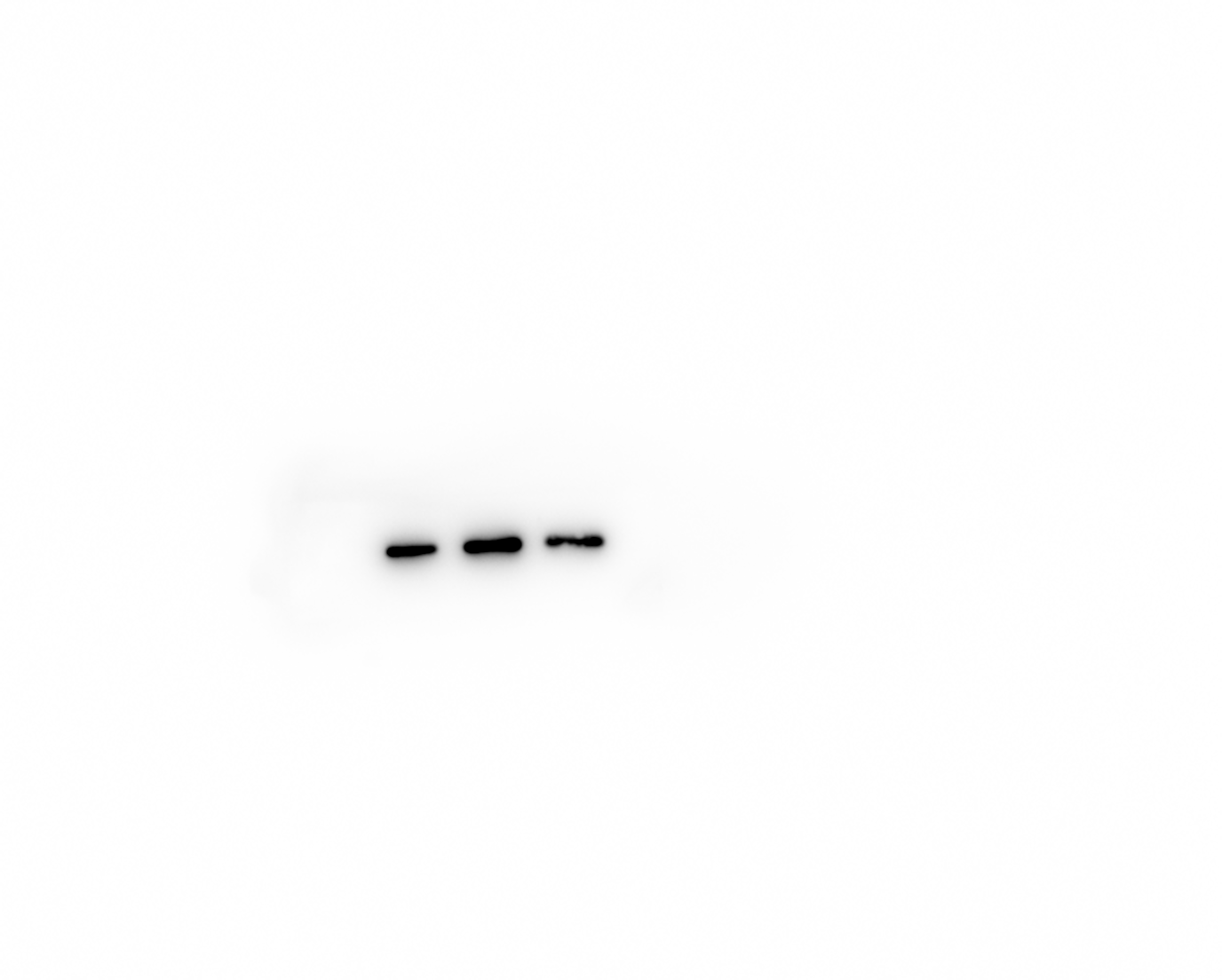

Supplement: DATA SHEET S1 — A full scan of the entire original gel(s). [file Data_Sheet_1.zip › original image files/Figure 3C/J82/pSTAT3.Tif]

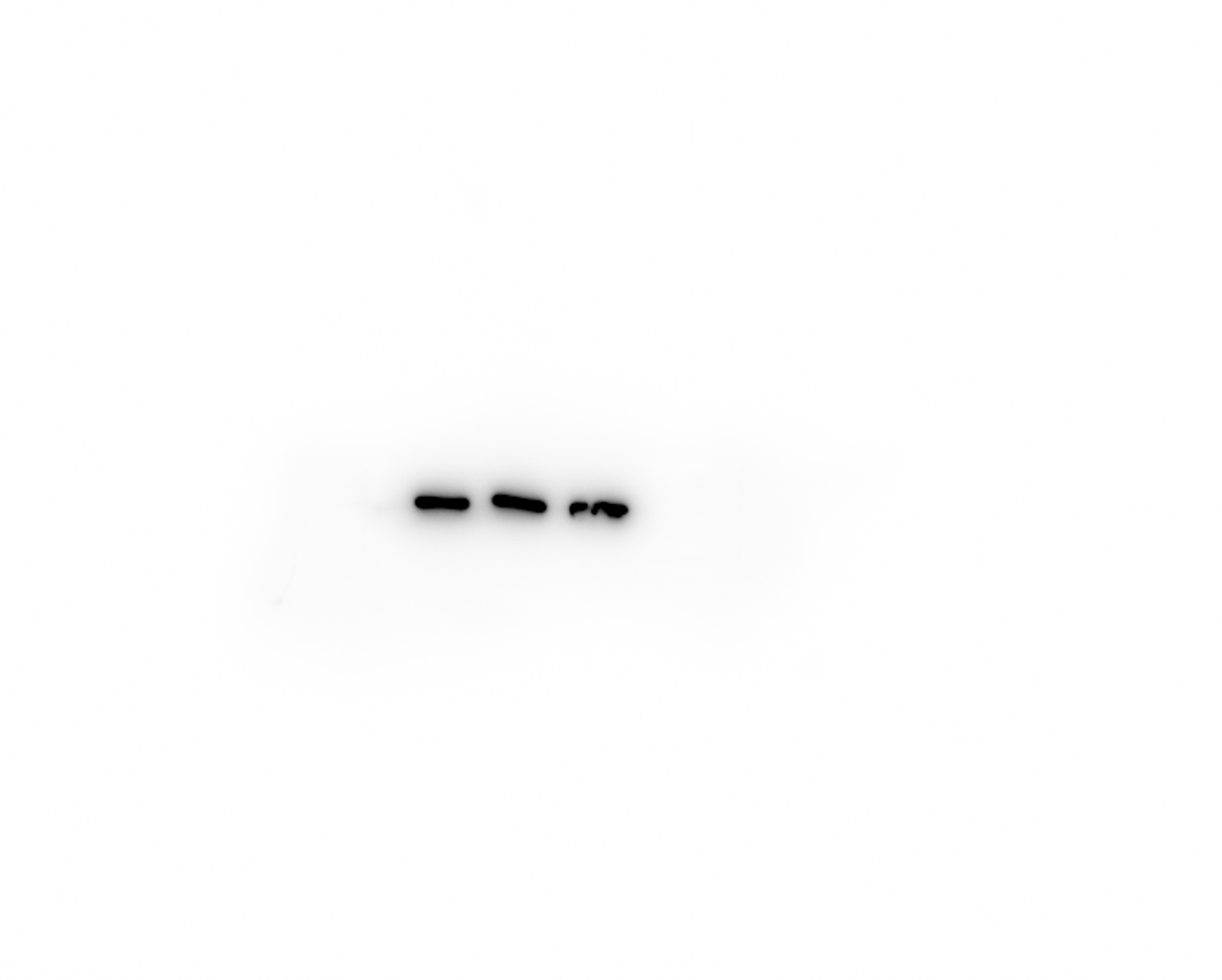

Supplement: DATA SHEET S1 — A full scan of the entire original gel(s). [file Data_Sheet_1.zip › original image files/Figure 3C/T24/GAPDH.Tif]

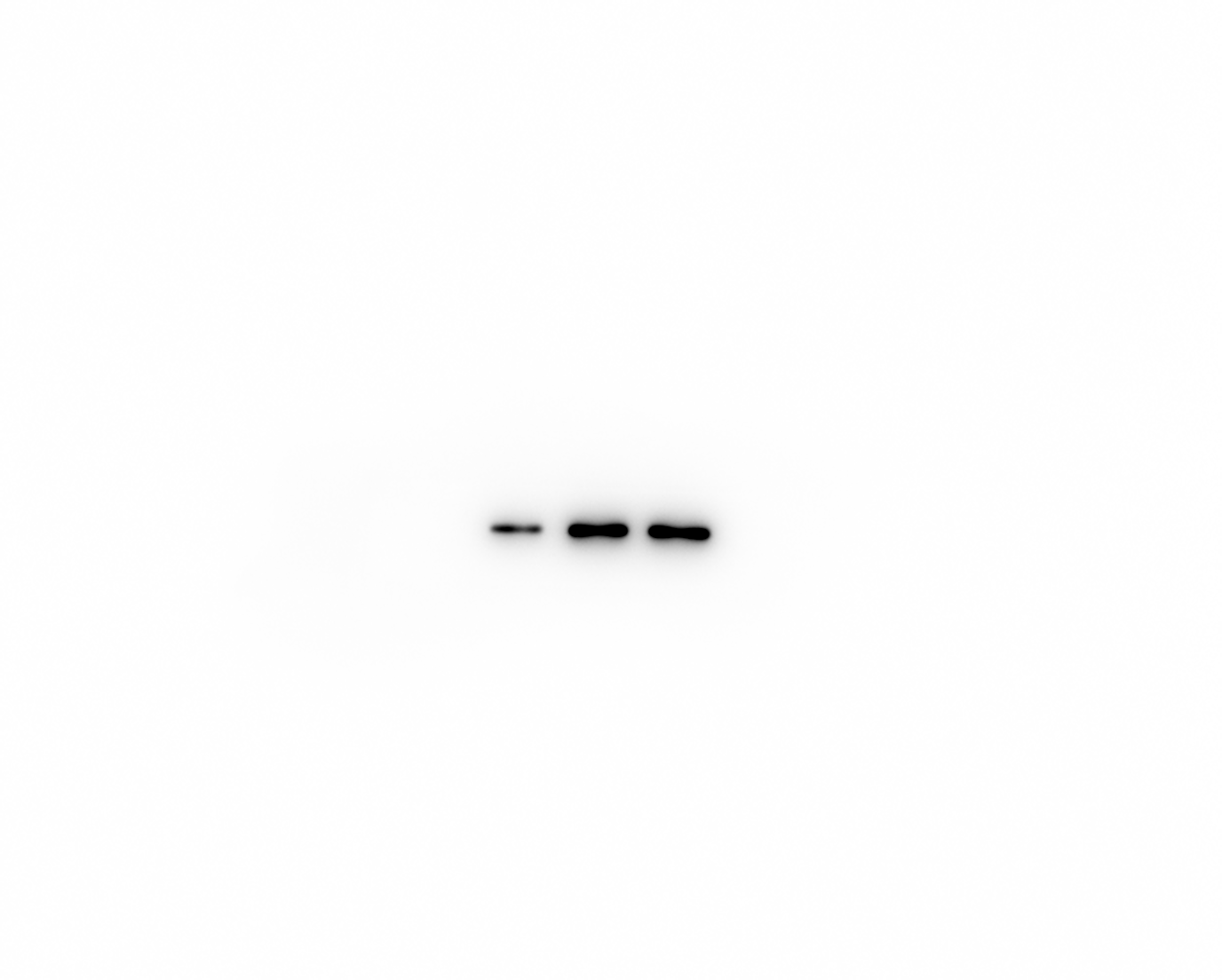

Supplement: DATA SHEET S1 — A full scan of the entire original gel(s). [file Data_Sheet_1.zip › original image files/Figure 3C/T24/JAK2.Tif]

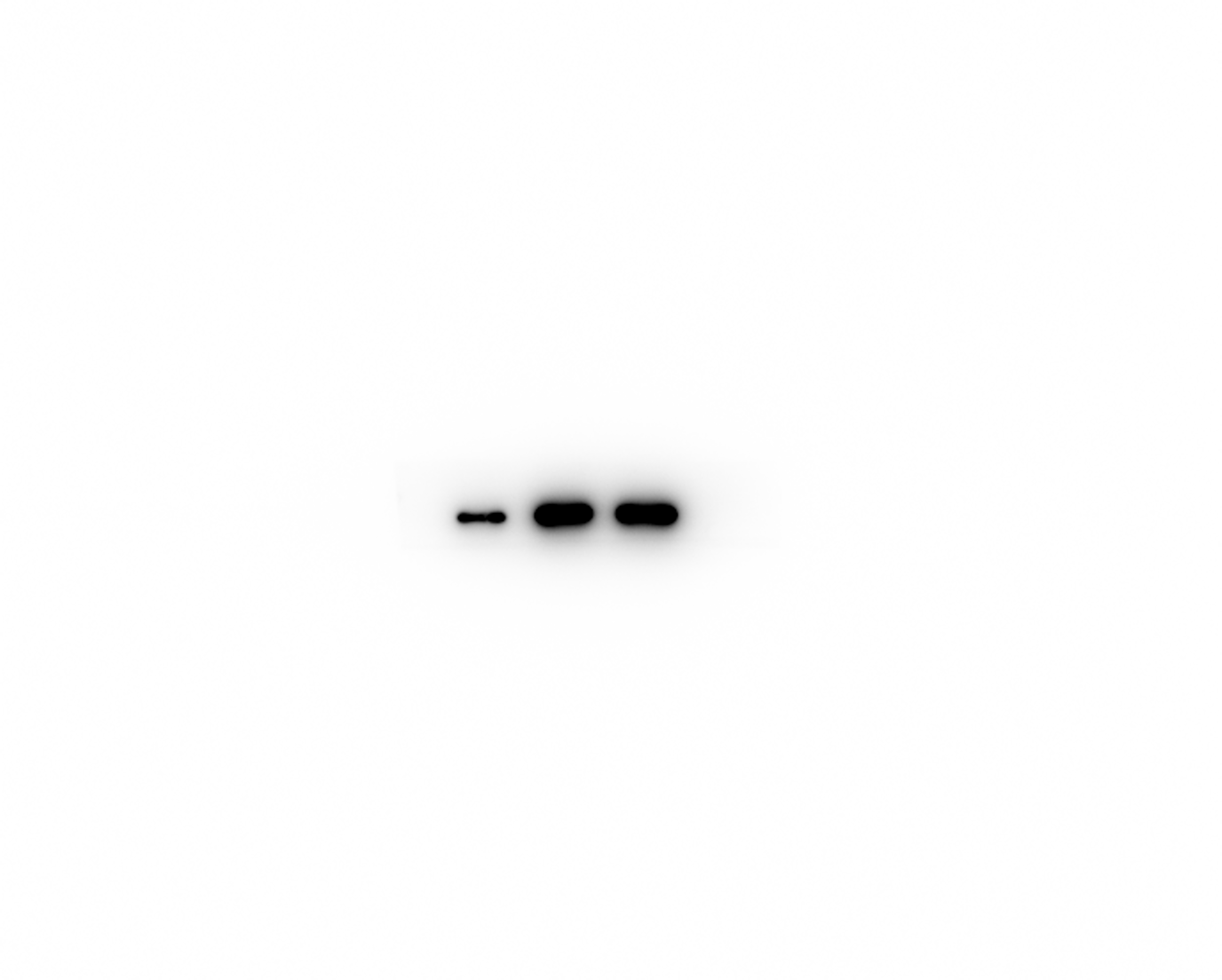

Supplement: DATA SHEET S1 — A full scan of the entire original gel(s). [file Data_Sheet_1.zip › original image files/Figure 3C/T24/RAC3.Tif]

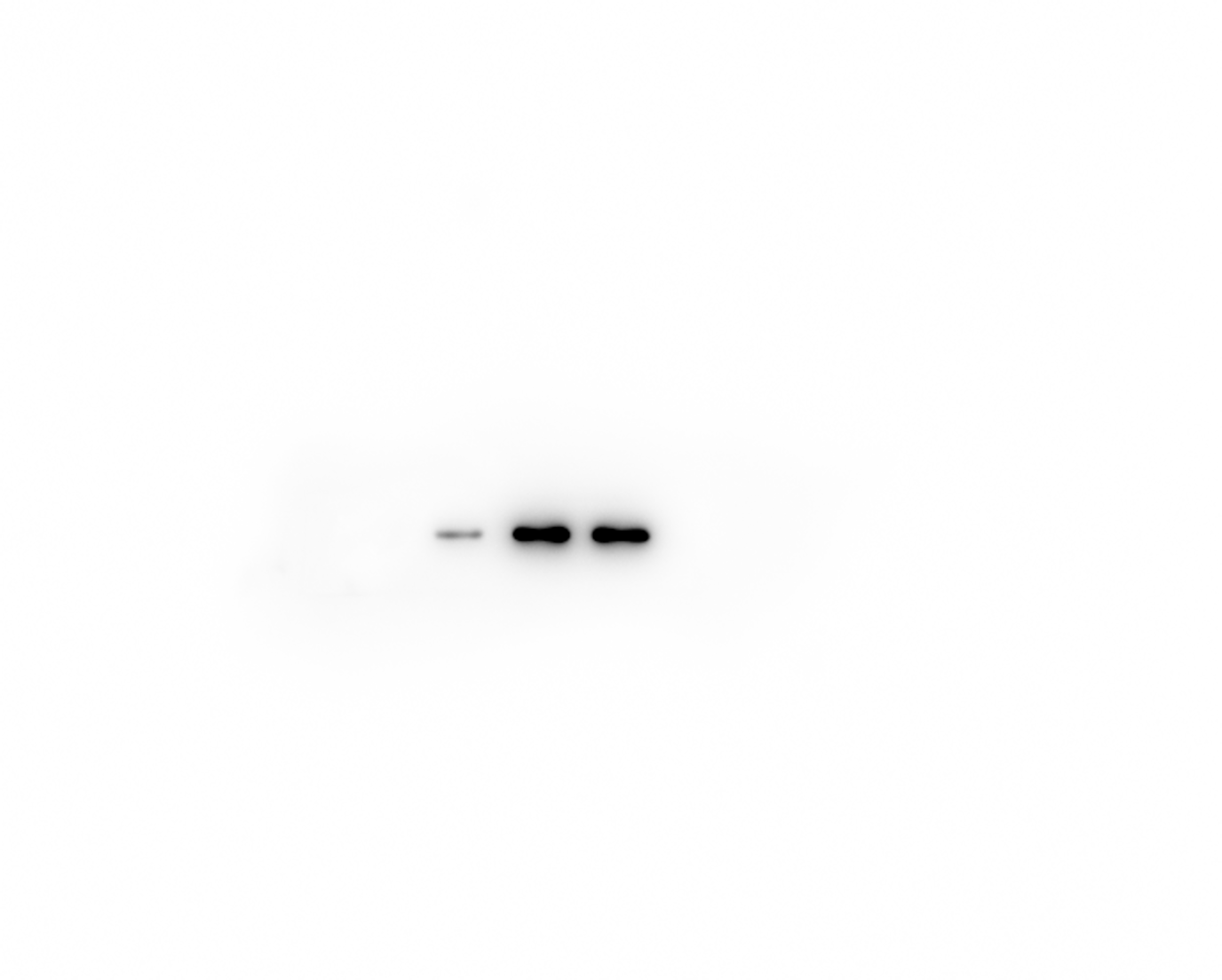

Supplement: DATA SHEET S1 — A full scan of the entire original gel(s). [file Data_Sheet_1.zip › original image files/Figure 3C/T24/STAT3.Tif]

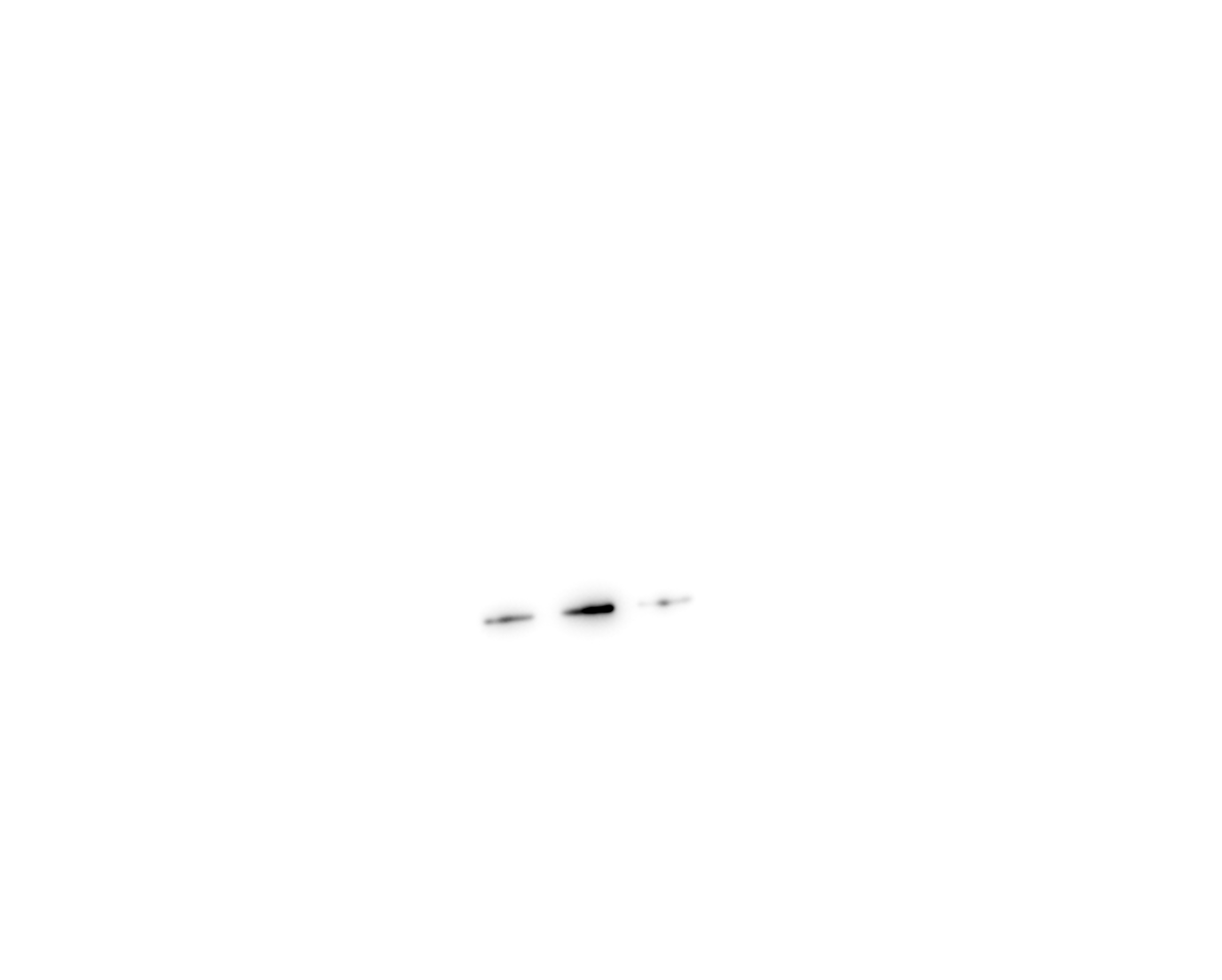

Supplement: DATA SHEET S1 — A full scan of the entire original gel(s). [file Data_Sheet_1.zip › original image files/Figure 3C/T24/c-MYC.Tif]

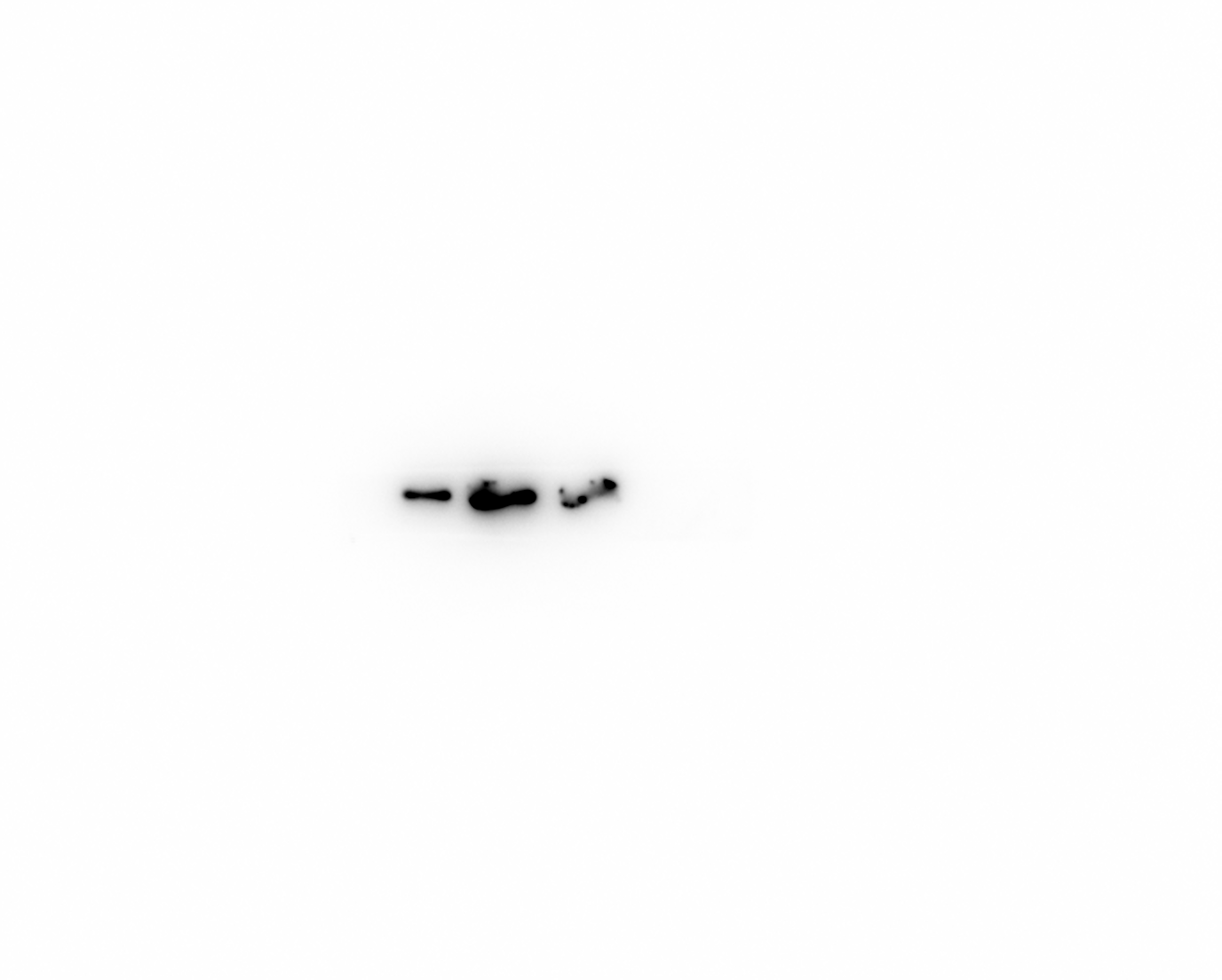

Supplement: DATA SHEET S1 — A full scan of the entire original gel(s). [file Data_Sheet_1.zip › original image files/Figure 3C/T24/pJAK2.Tif]

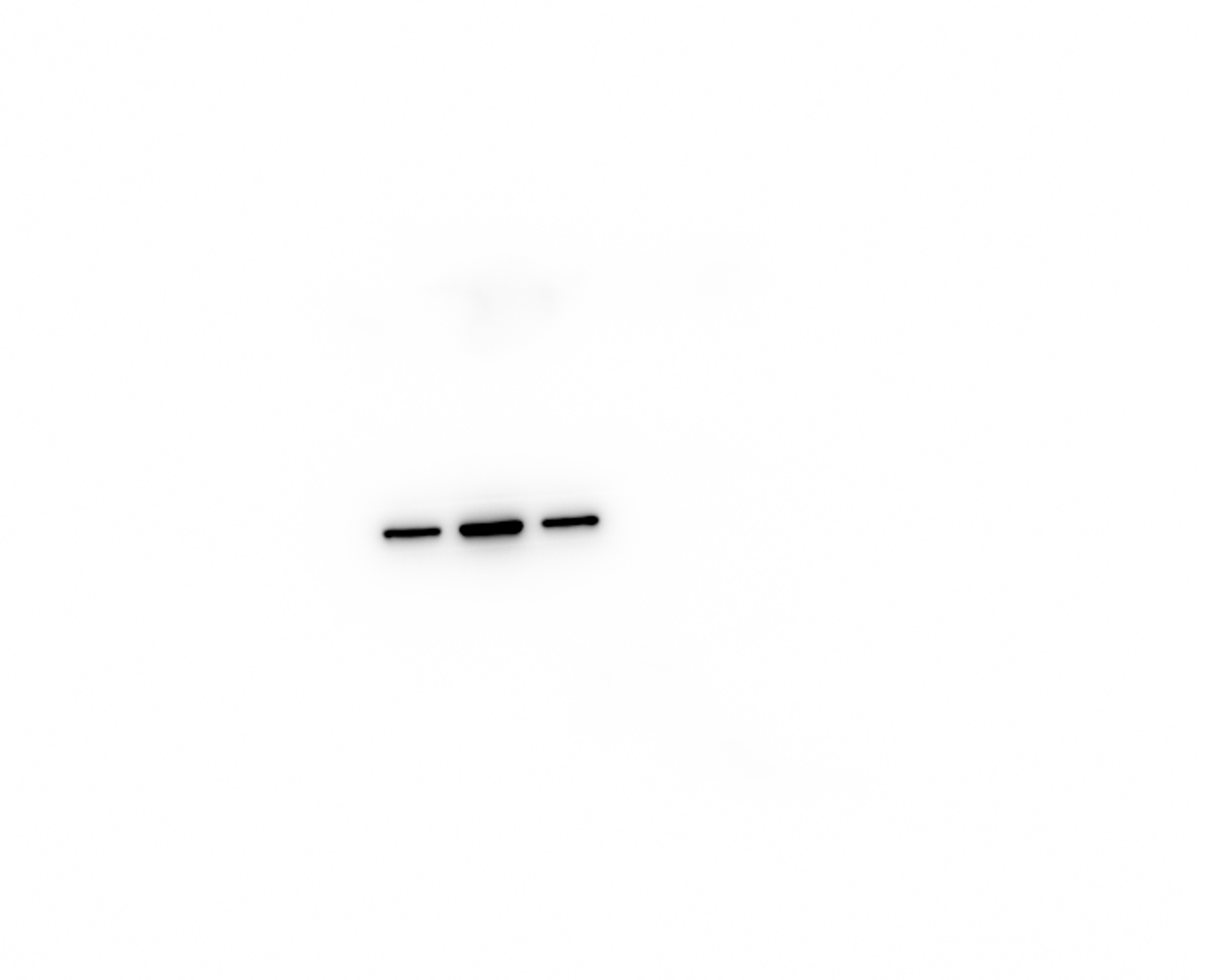

Supplement: DATA SHEET S1 — A full scan of the entire original gel(s). [file Data_Sheet_1.zip › original image files/Figure 3C/T24/pSTAT3.Tif]

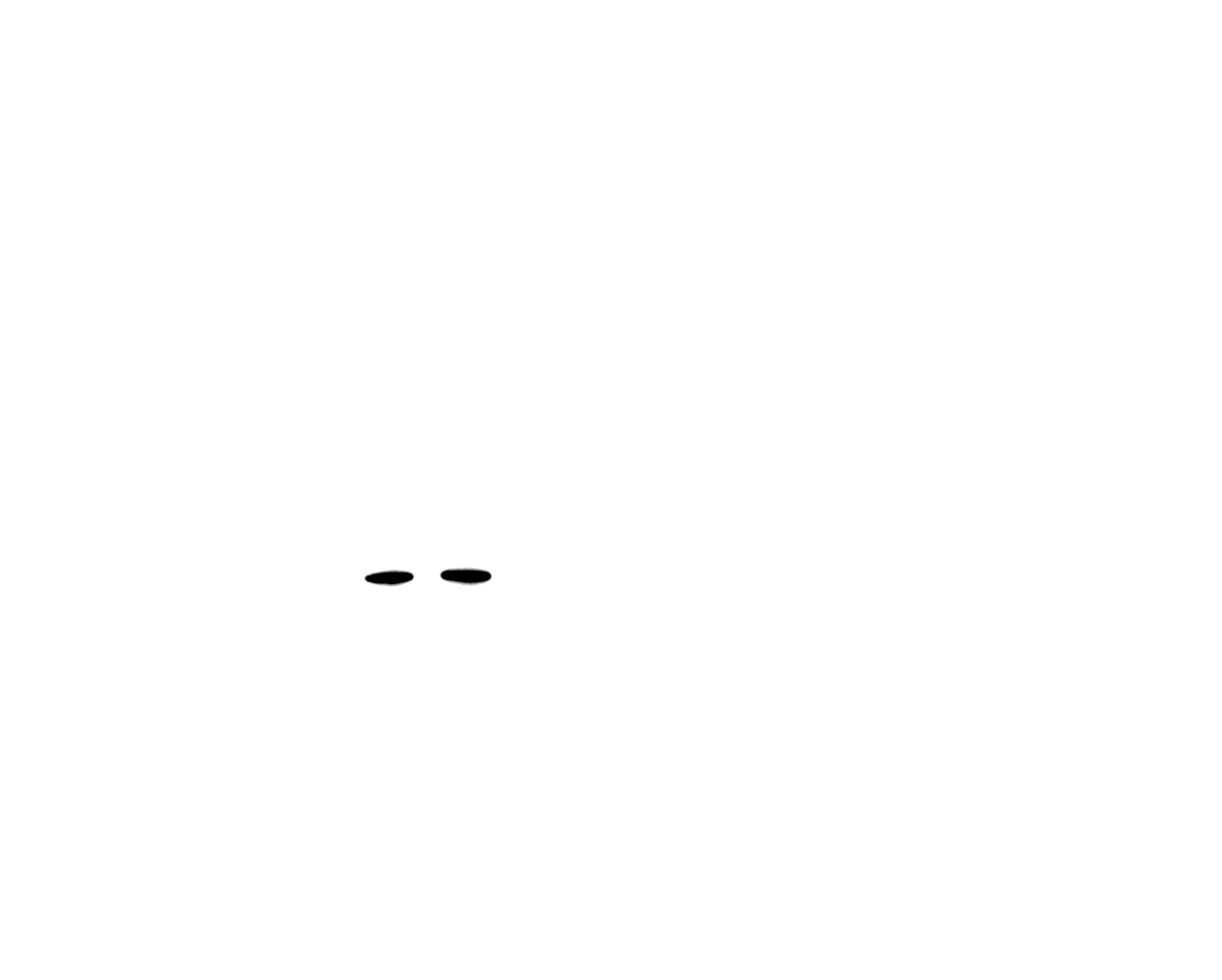

Supplement: DATA SHEET S1 — A full scan of the entire original gel(s). [file Data_Sheet_1.zip › original image files/Figure 4F/J82/RAC3-knockdown/GAPDH.tif]

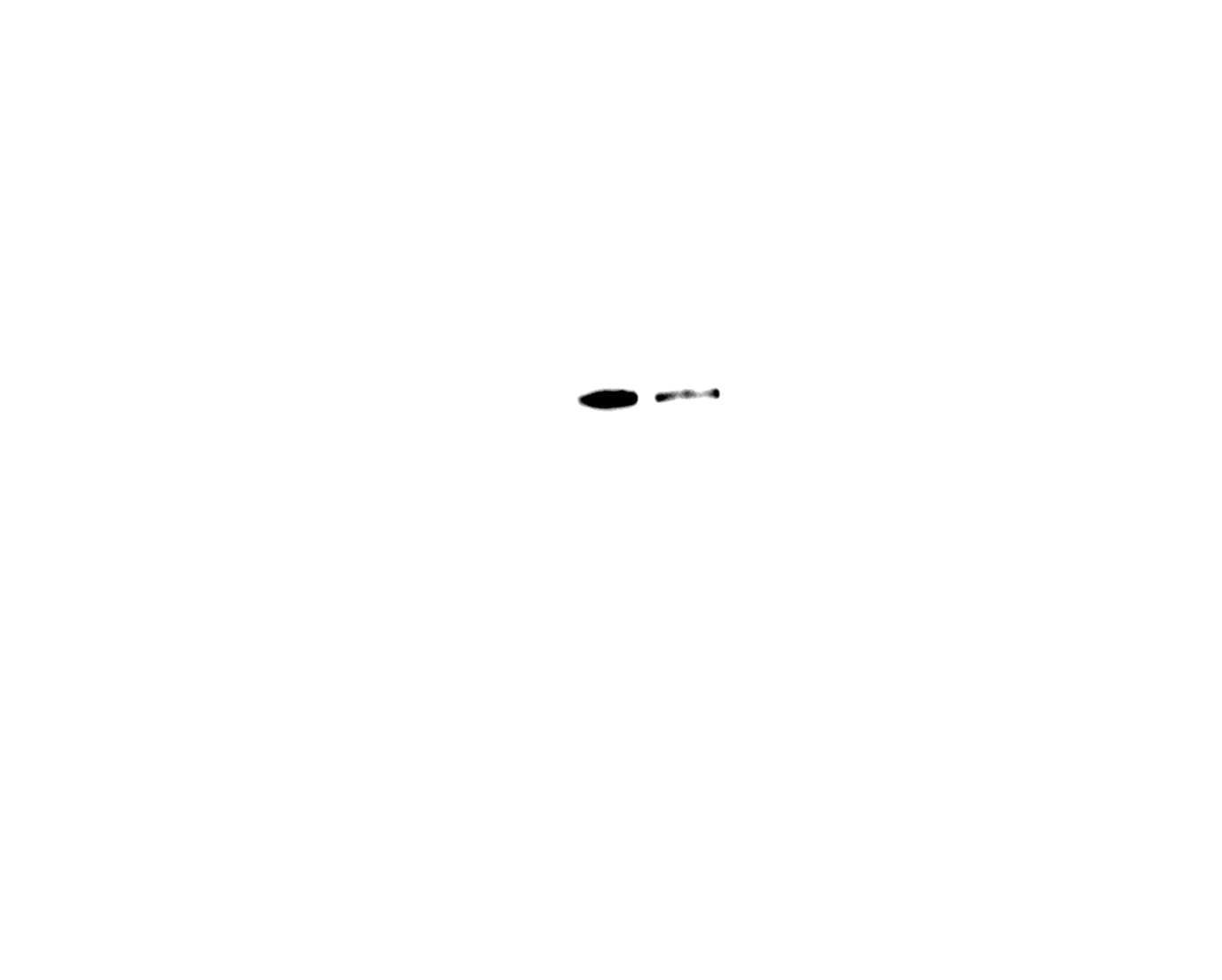

Supplement: DATA SHEET S1 — A full scan of the entire original gel(s). [file Data_Sheet_1.zip › original image files/Figure 4F/J82/RAC3-knockdown/PYCR1.tif]

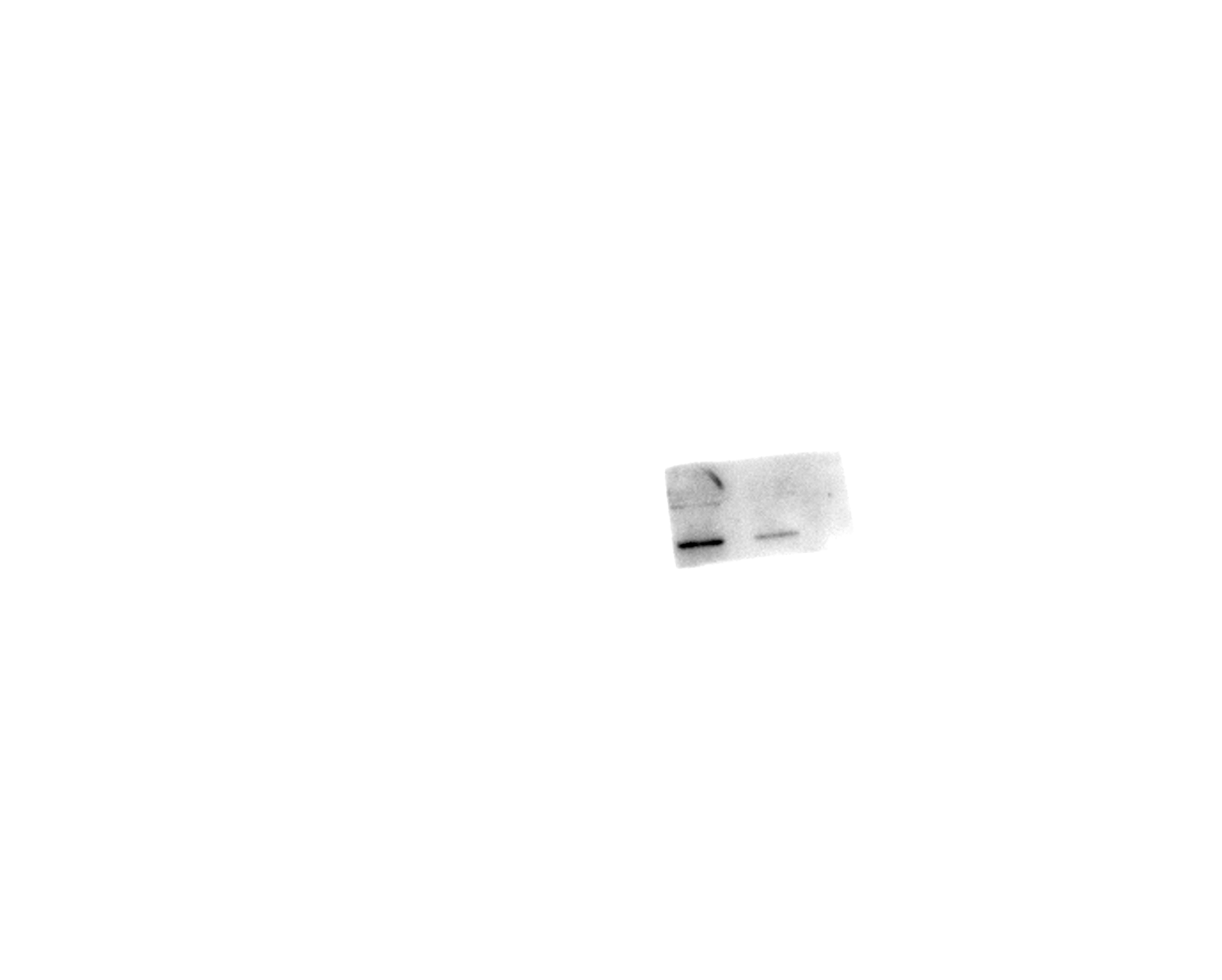

Supplement: DATA SHEET S1 — A full scan of the entire original gel(s). [file Data_Sheet_1.zip › original image files/Figure 4F/J82/RAC3-knockdown/RAC3.Tif]

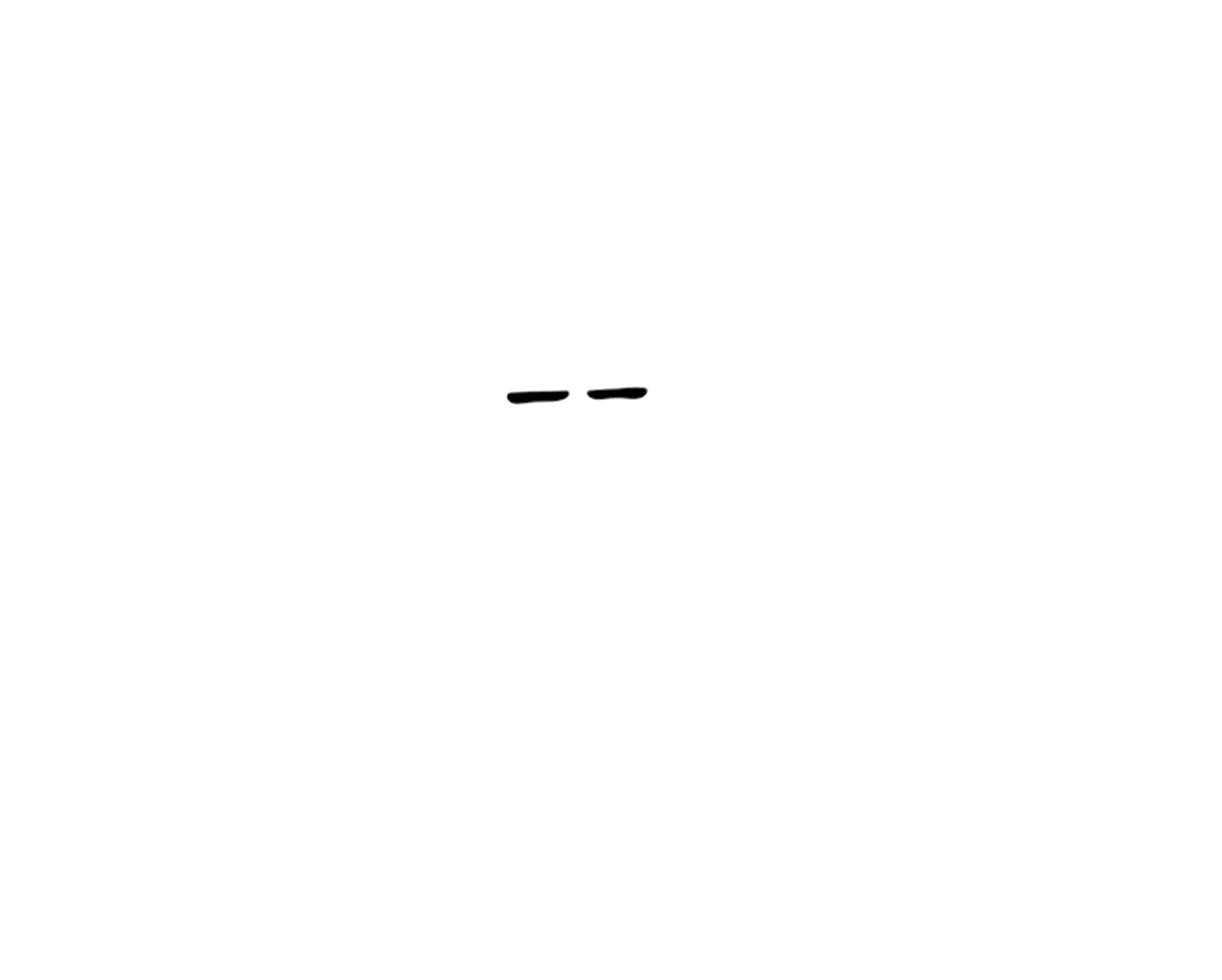

Supplement: DATA SHEET S1 — A full scan of the entire original gel(s). [file Data_Sheet_1.zip › original image files/Figure 4F/J82/RAC3-overexpression/GAPDH.tif]

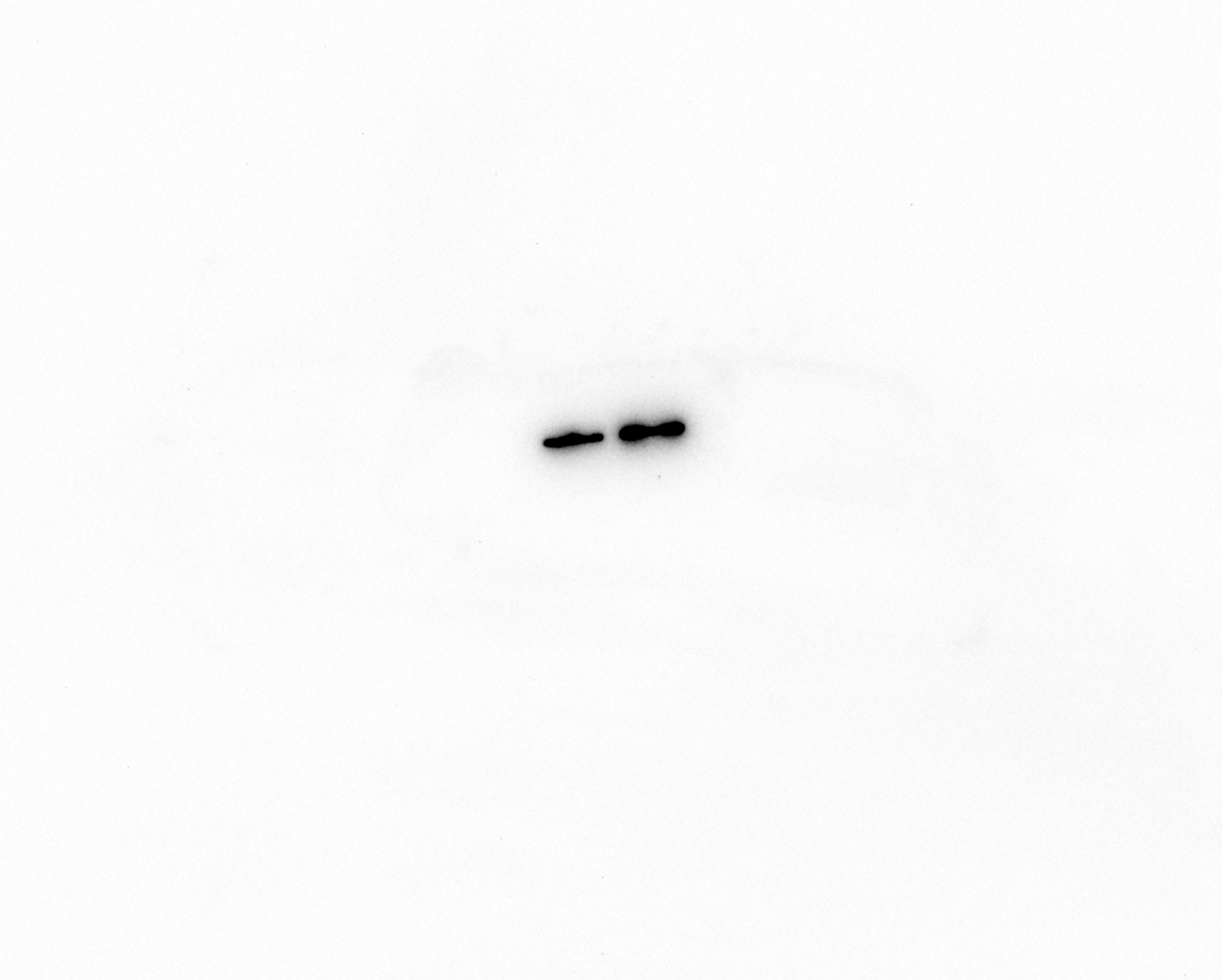

Supplement: DATA SHEET S1 — A full scan of the entire original gel(s). [file Data_Sheet_1.zip › original image files/Figure 4F/J82/RAC3-overexpression/PYCR1.Tif]

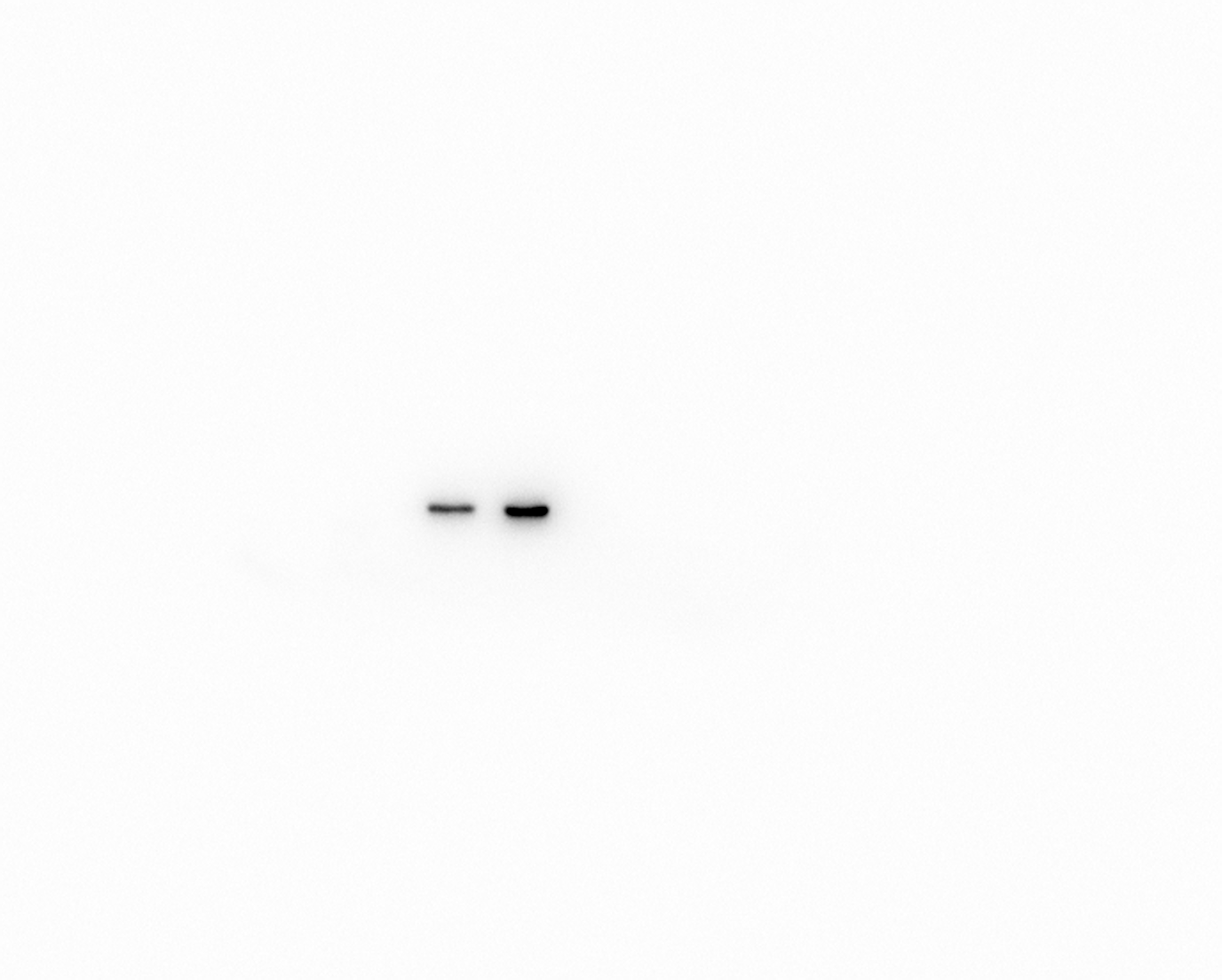

Supplement: DATA SHEET S1 — A full scan of the entire original gel(s). [file Data_Sheet_1.zip › original image files/Figure 4F/J82/RAC3-overexpression/RAC3.Tif]

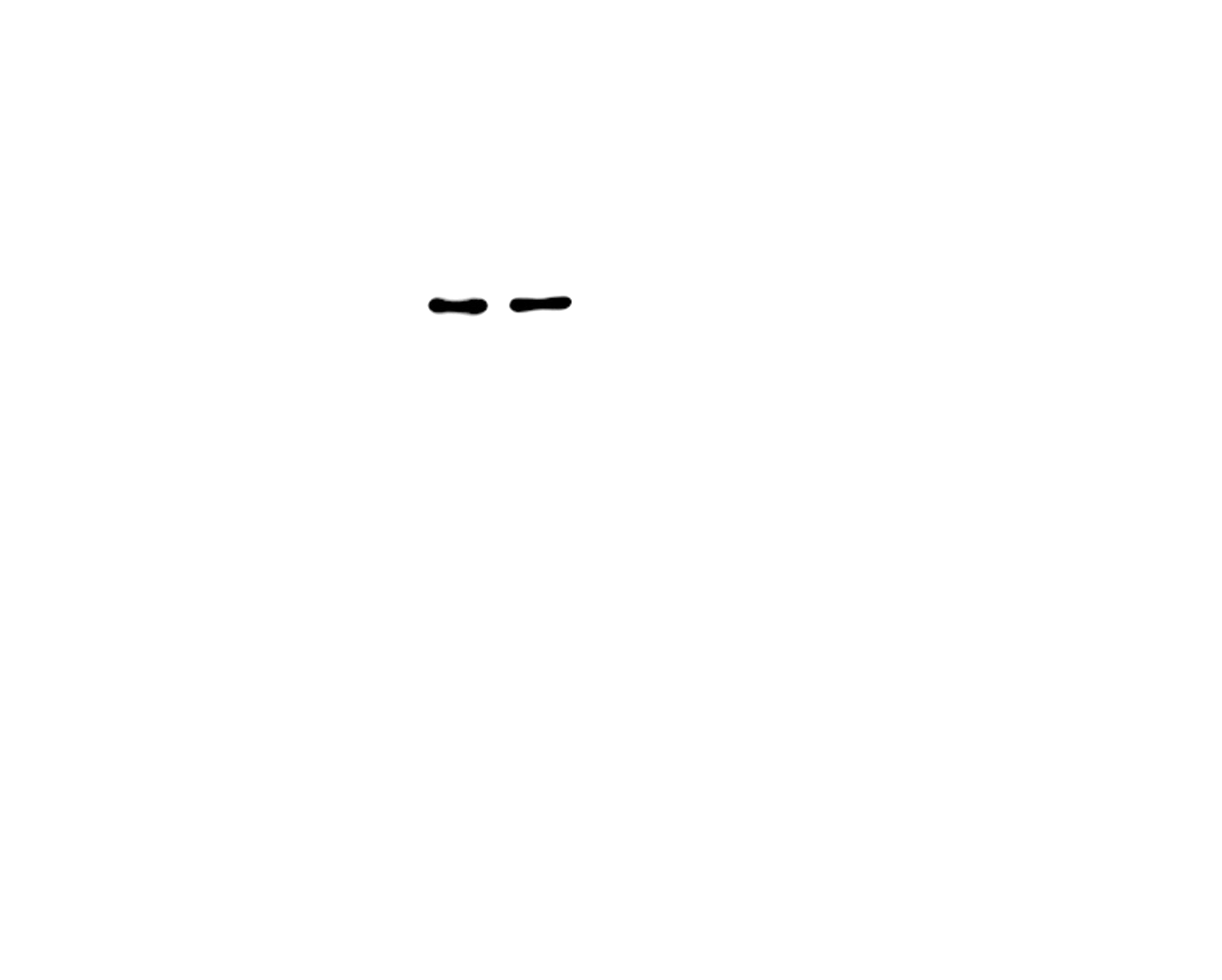

Supplement: DATA SHEET S1 — A full scan of the entire original gel(s). [file Data_Sheet_1.zip › original image files/Figure 4F/T24/RAC3-knockdown/GAPDH.tif]

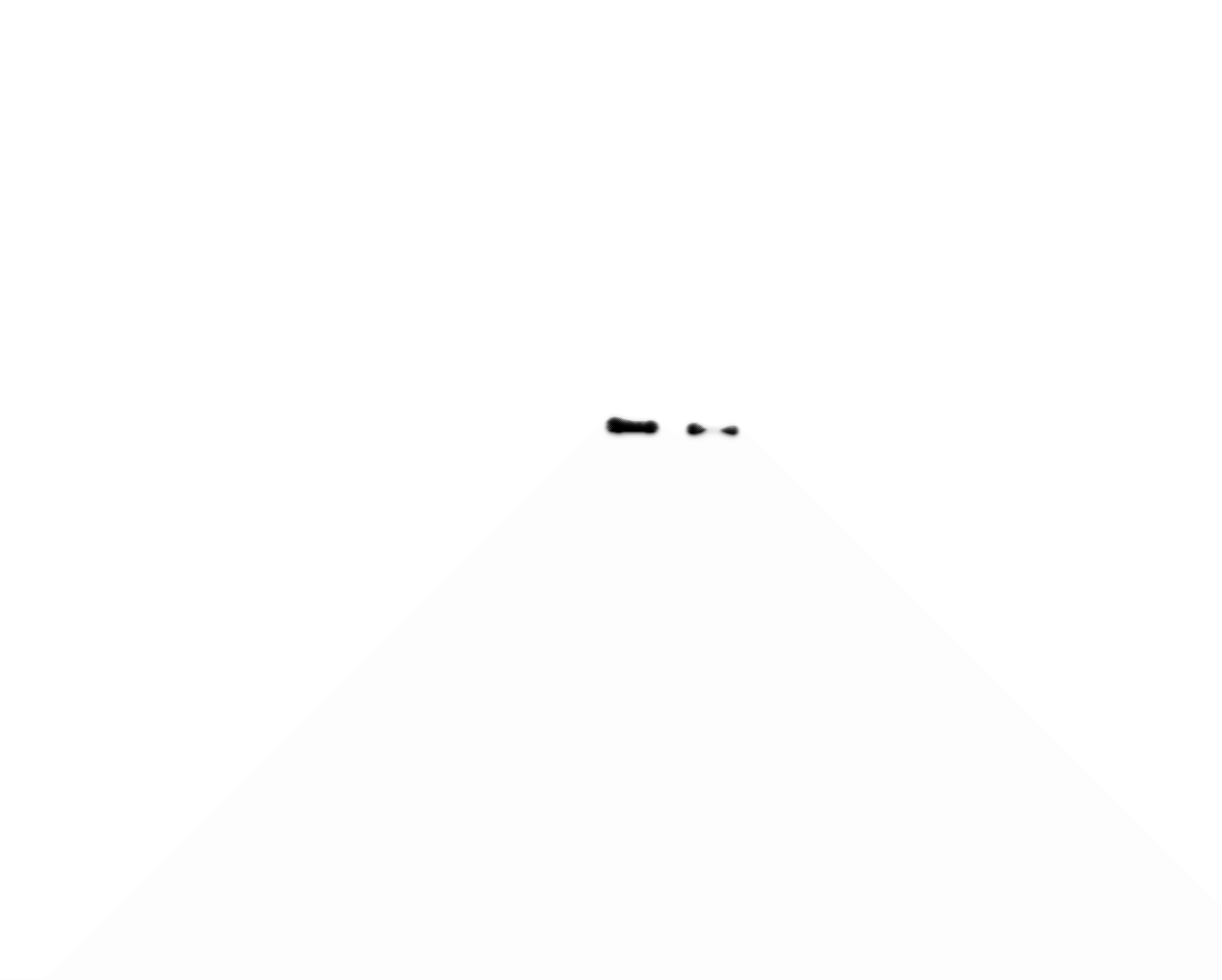

Supplement: DATA SHEET S1 — A full scan of the entire original gel(s). [file Data_Sheet_1.zip › original image files/Figure 4F/T24/RAC3-knockdown/PYCR1.tif]

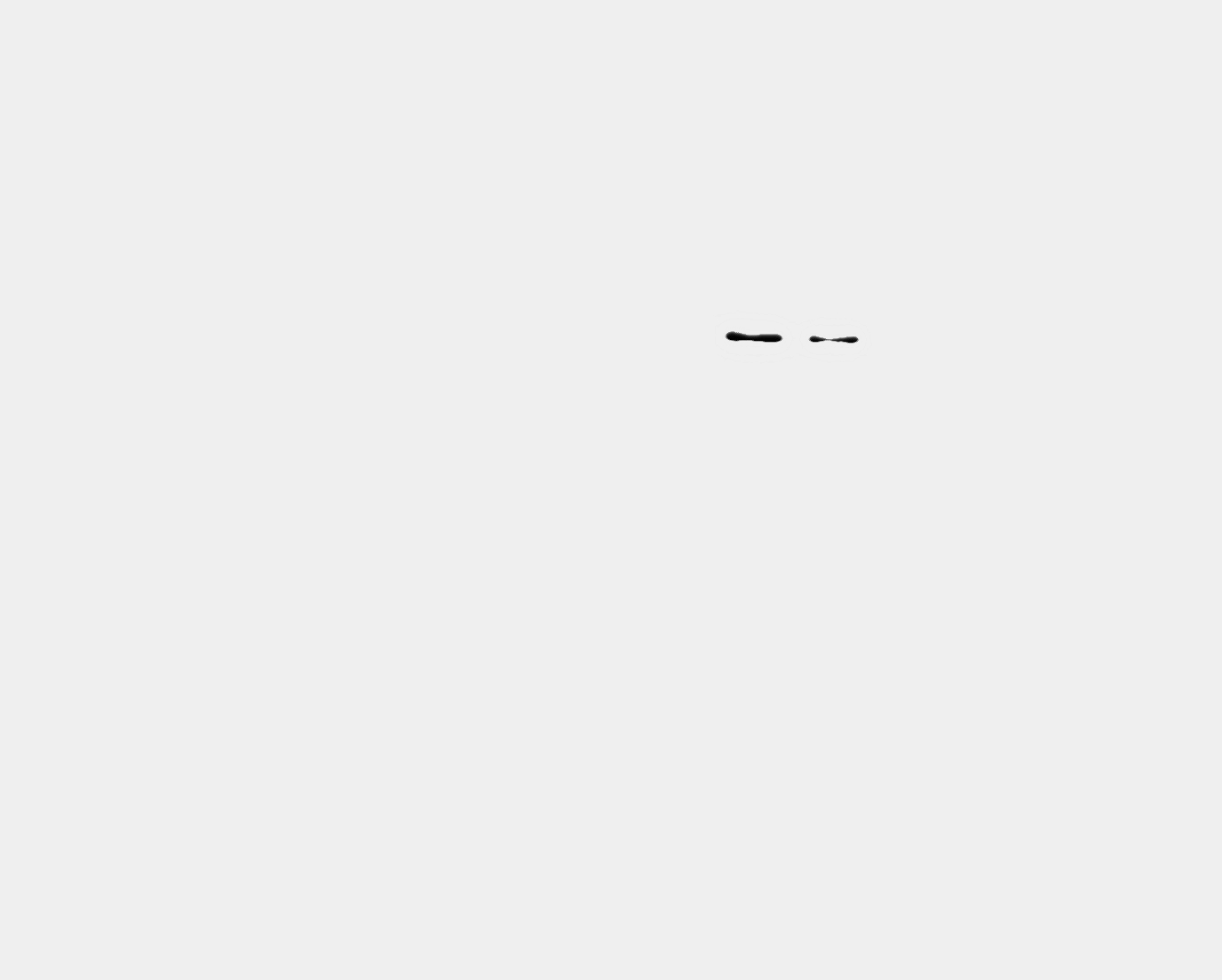

Supplement: DATA SHEET S1 — A full scan of the entire original gel(s). [file Data_Sheet_1.zip › original image files/Figure 4F/T24/RAC3-knockdown/RAC3.Tif]

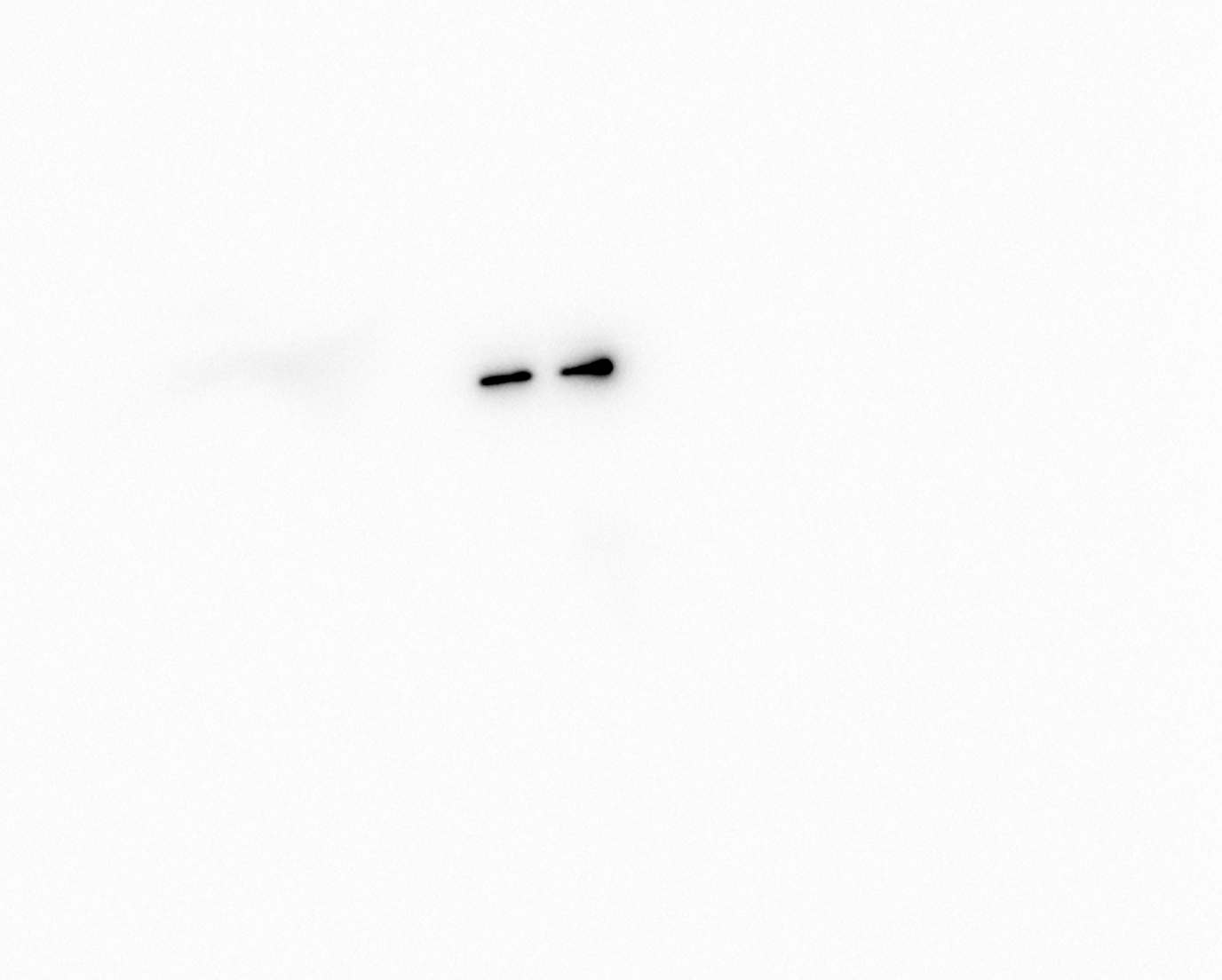

Supplement: DATA SHEET S1 — A full scan of the entire original gel(s). [file Data_Sheet_1.zip › original image files/Figure 4F/T24/RAC3-overexpression/GAPDH.Tif]

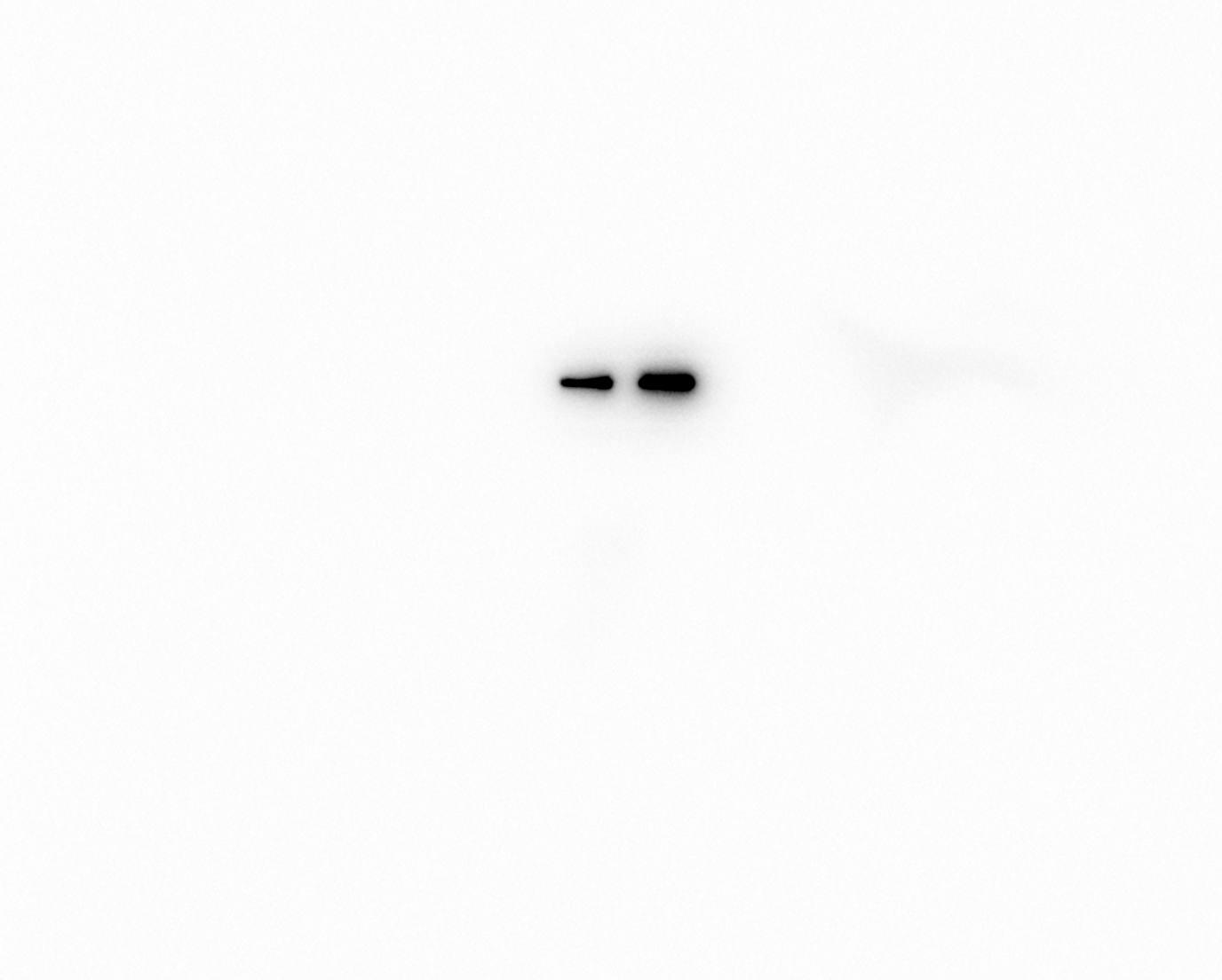

Supplement: DATA SHEET S1 — A full scan of the entire original gel(s). [file Data_Sheet_1.zip › original image files/Figure 4F/T24/RAC3-overexpression/PYCR1.Tif]

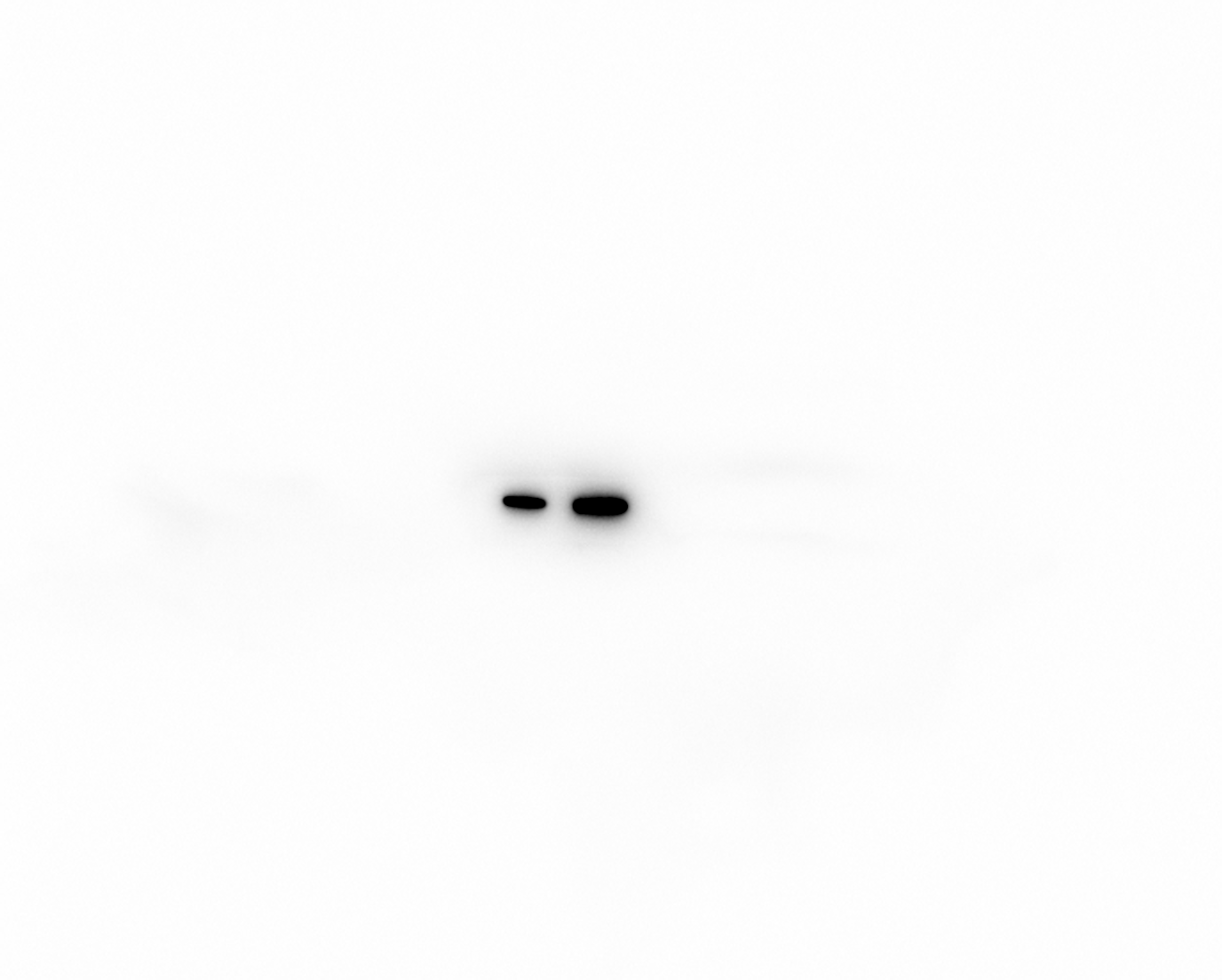

Supplement: DATA SHEET S1 — A full scan of the entire original gel(s). [file Data_Sheet_1.zip › original image files/Figure 4F/T24/RAC3-overexpression/RAC3.Tif]

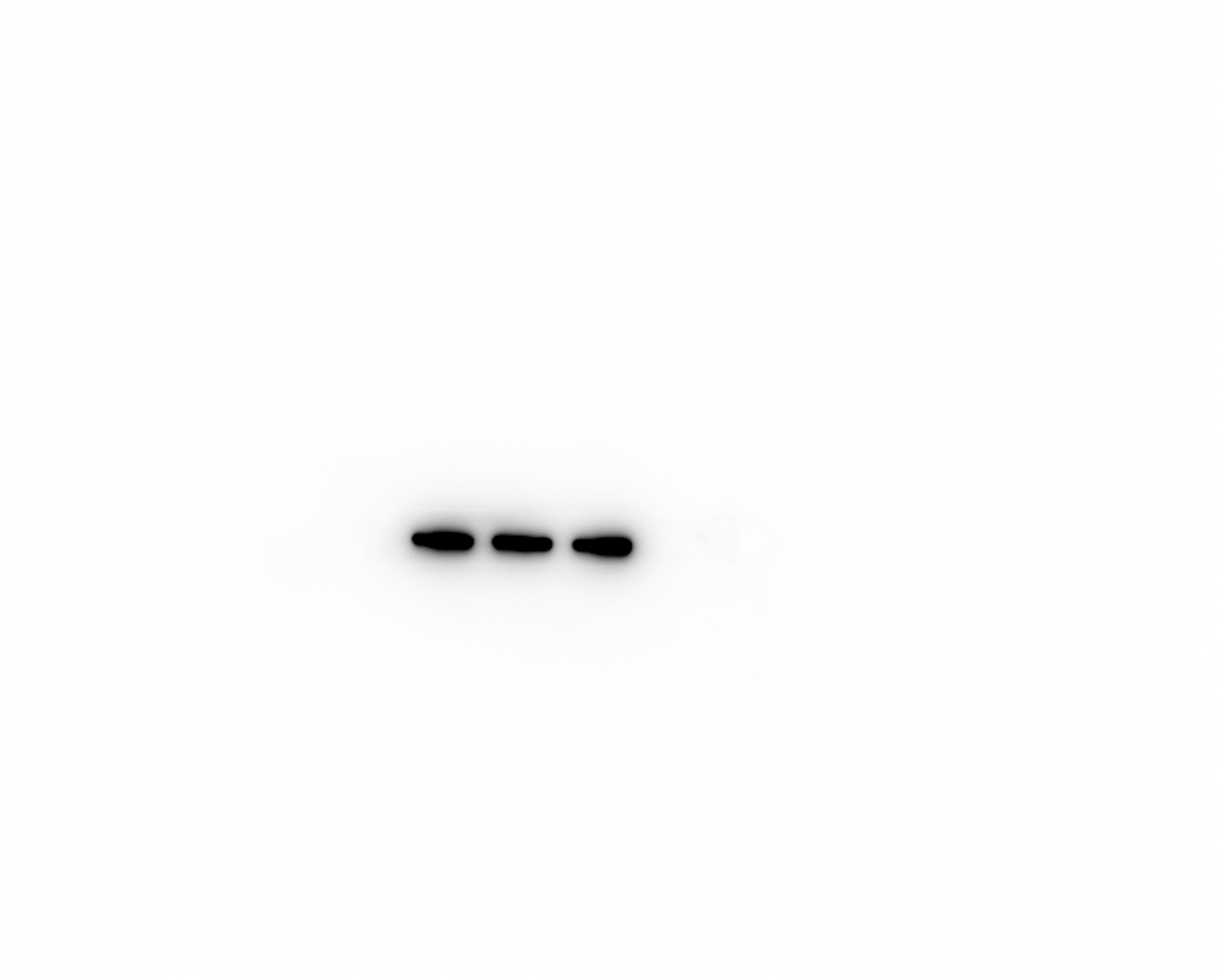

Supplement: DATA SHEET S1 — A full scan of the entire original gel(s). [file Data_Sheet_1.zip › original image files/Figure 6D/J82/GAPDH.Tif]

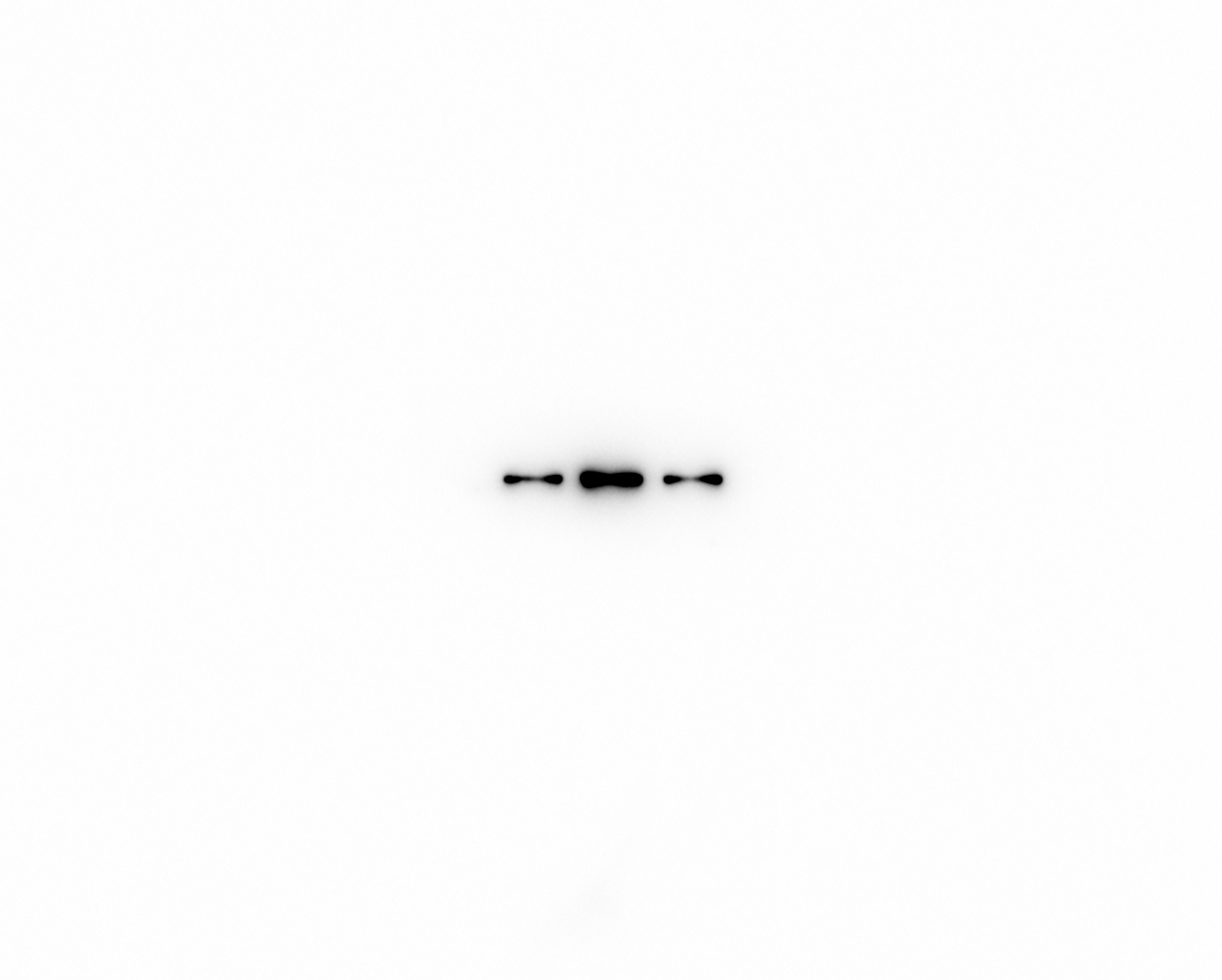

Supplement: DATA SHEET S1 — A full scan of the entire original gel(s). [file Data_Sheet_1.zip › original image files/Figure 6D/J82/JAK2.Tif]

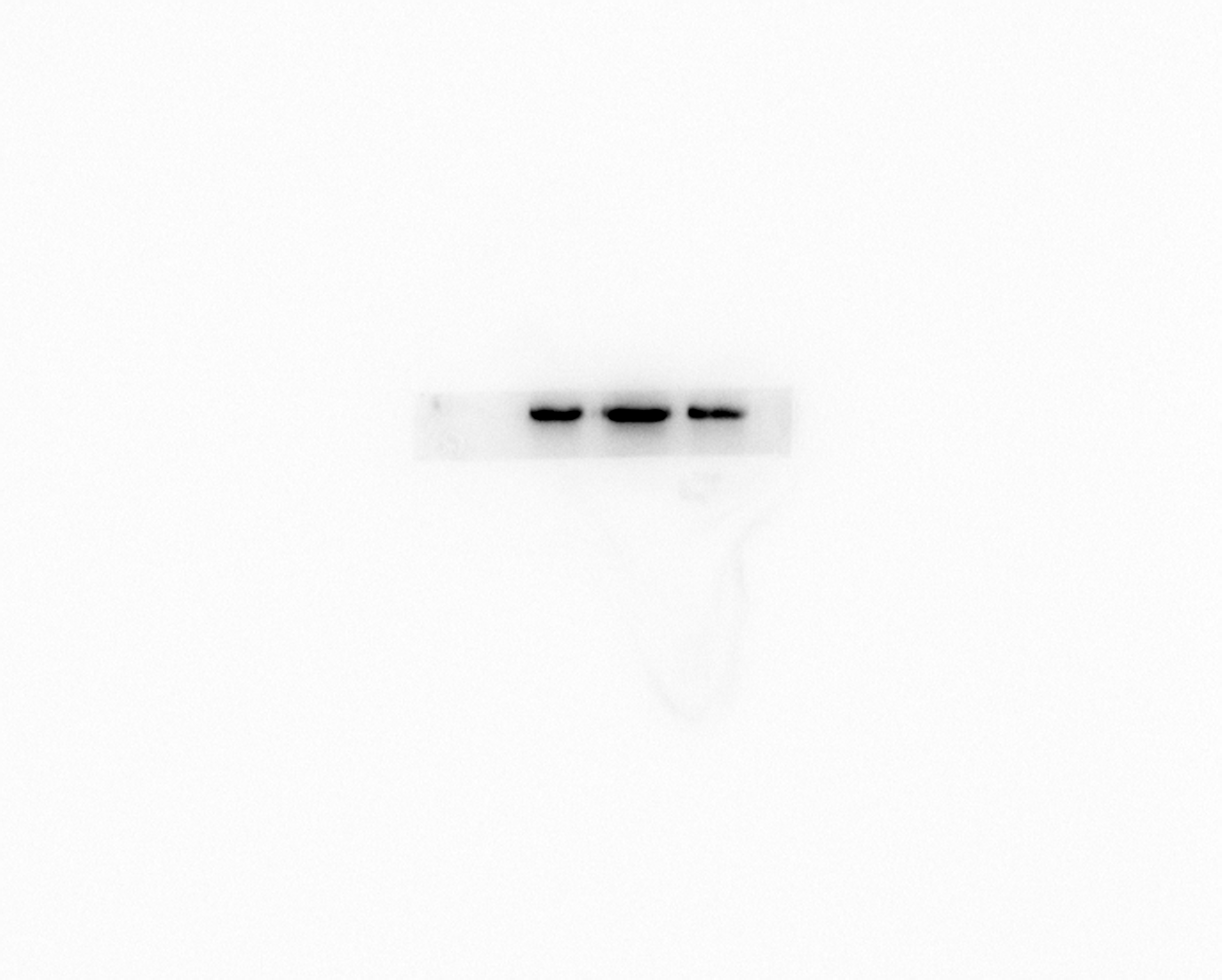

Supplement: DATA SHEET S1 — A full scan of the entire original gel(s). [file Data_Sheet_1.zip › original image files/Figure 6D/J82/P-JAK2.Tif]

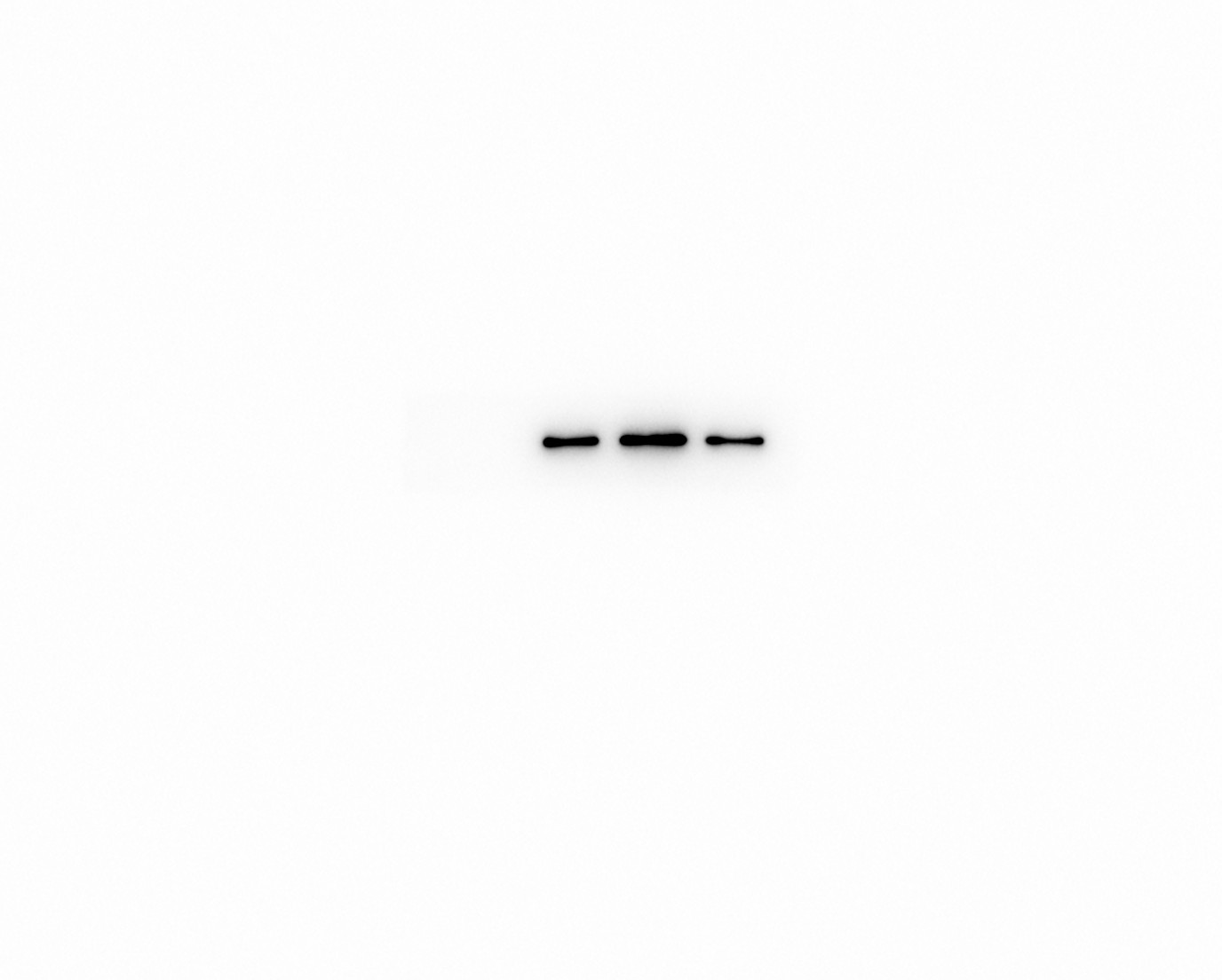

Supplement: DATA SHEET S1 — A full scan of the entire original gel(s). [file Data_Sheet_1.zip › original image files/Figure 6D/J82/PYCR1.Tif]

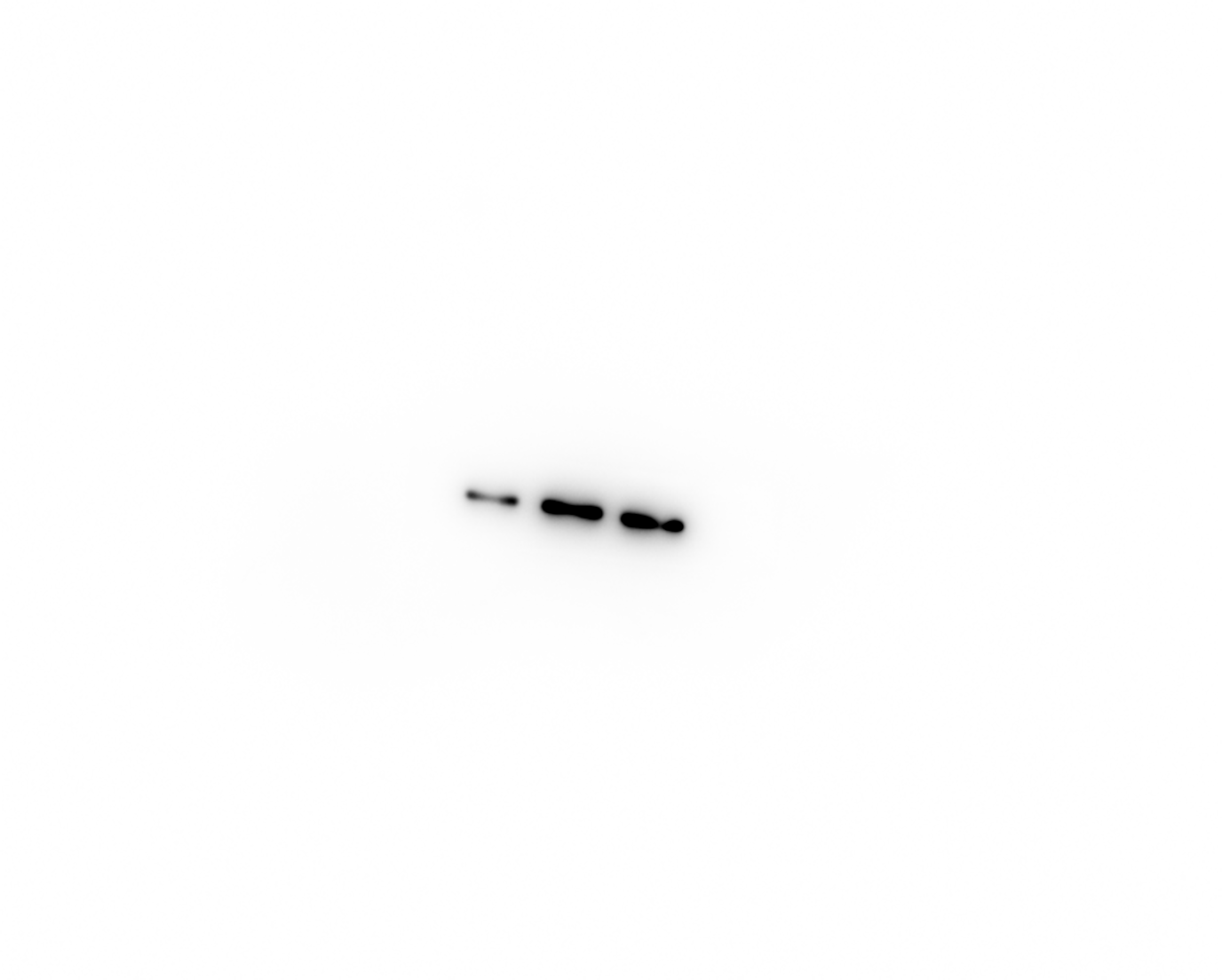

Supplement: DATA SHEET S1 — A full scan of the entire original gel(s). [file Data_Sheet_1.zip › original image files/Figure 6D/J82/RAC3.Tif]

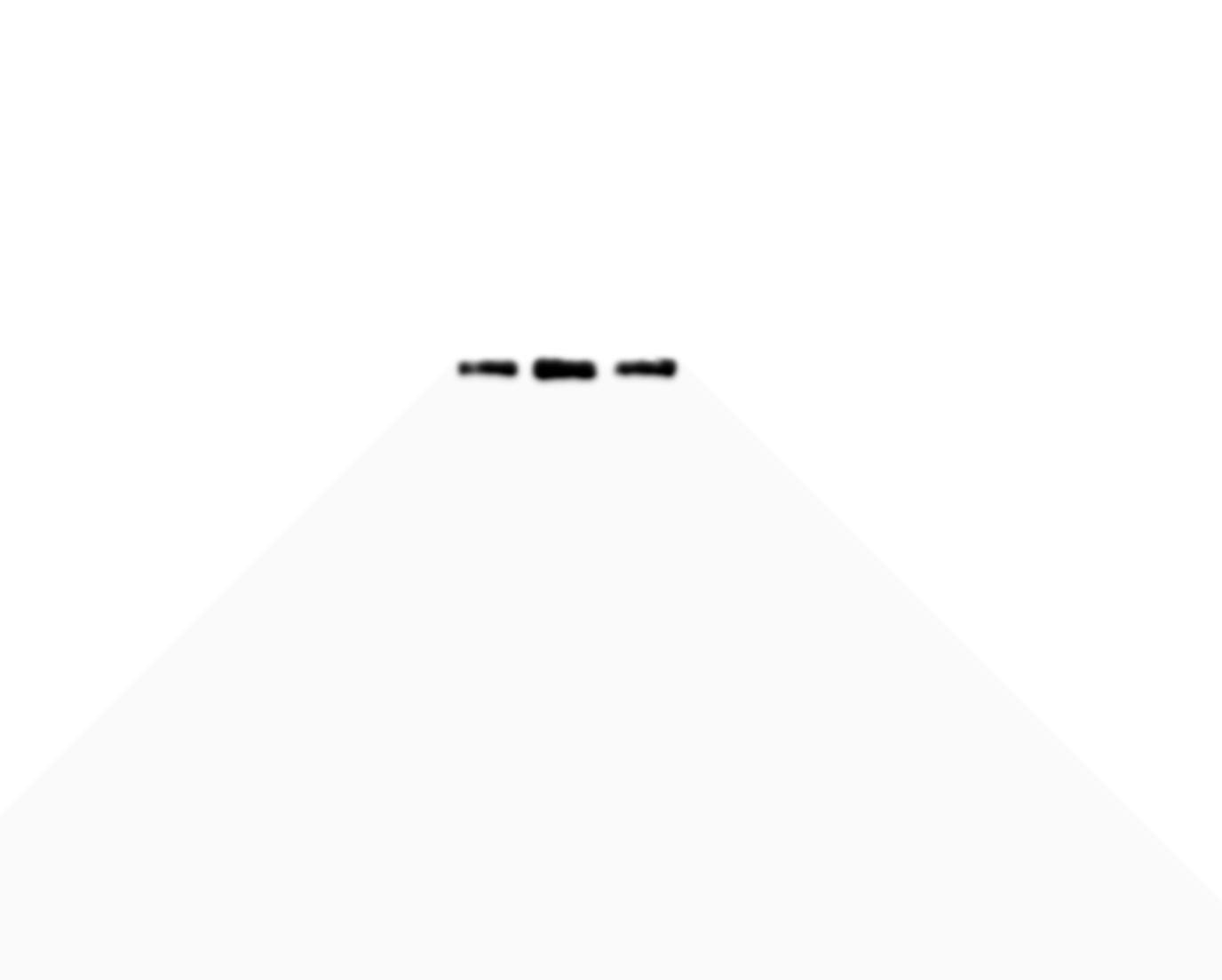

Supplement: DATA SHEET S1 — A full scan of the entire original gel(s). [file Data_Sheet_1.zip › original image files/Figure 6D/J82/STAT3.Tif]

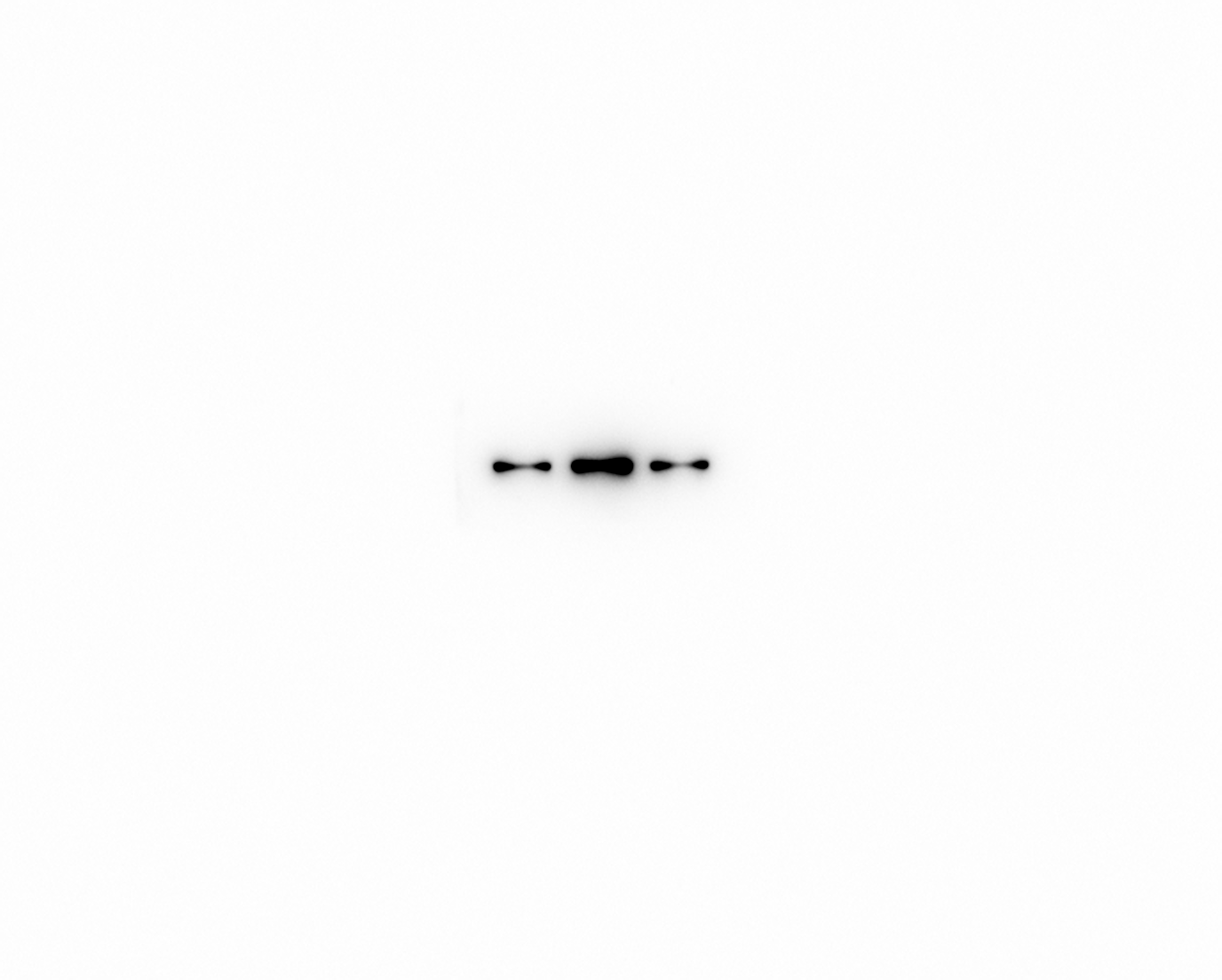

Supplement: DATA SHEET S1 — A full scan of the entire original gel(s). [file Data_Sheet_1.zip › original image files/Figure 6D/J82/cMYC.Tif]

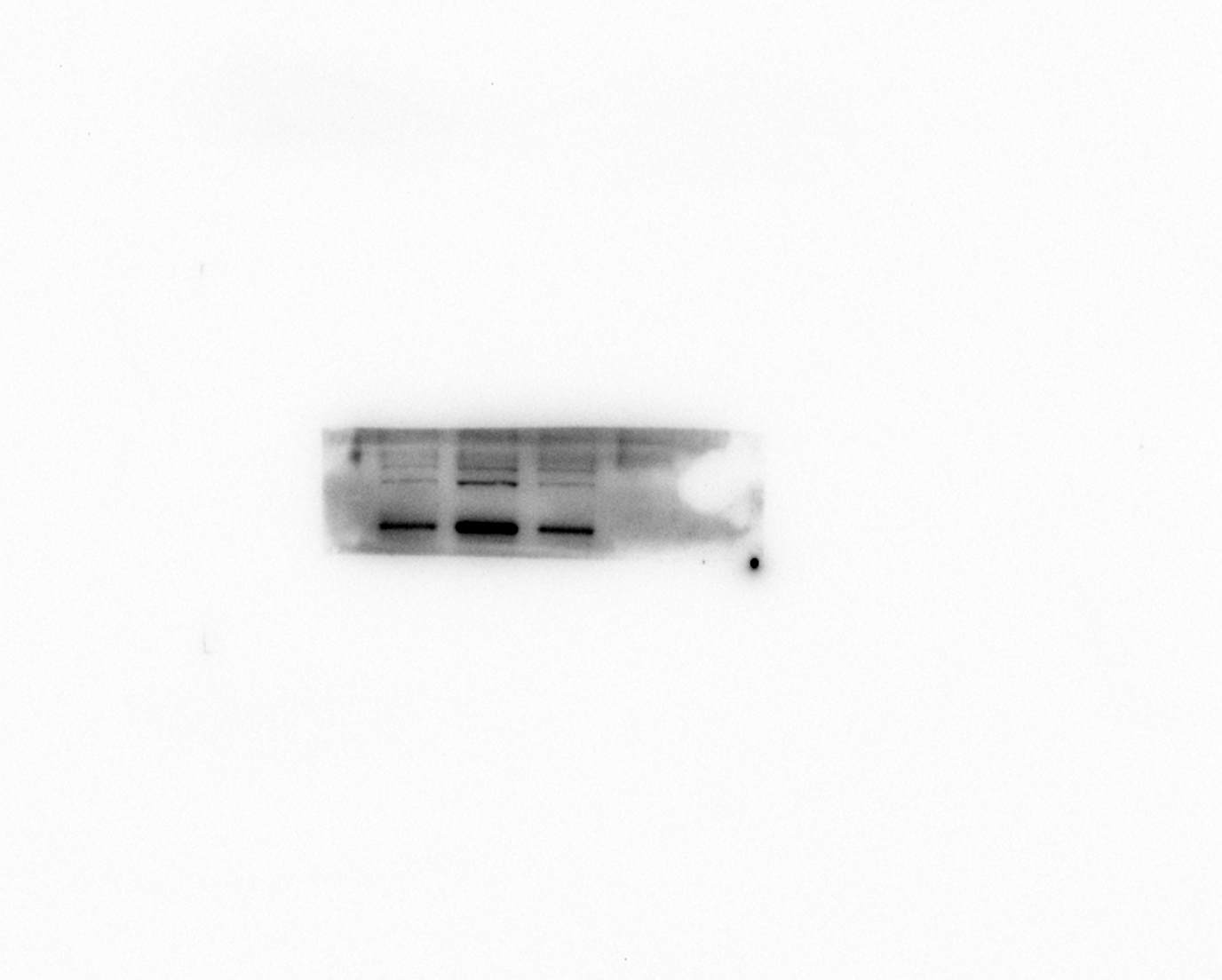

Supplement: DATA SHEET S1 — A full scan of the entire original gel(s). [file Data_Sheet_1.zip › original image files/Figure 6D/J82/pSTAT3.Tif]

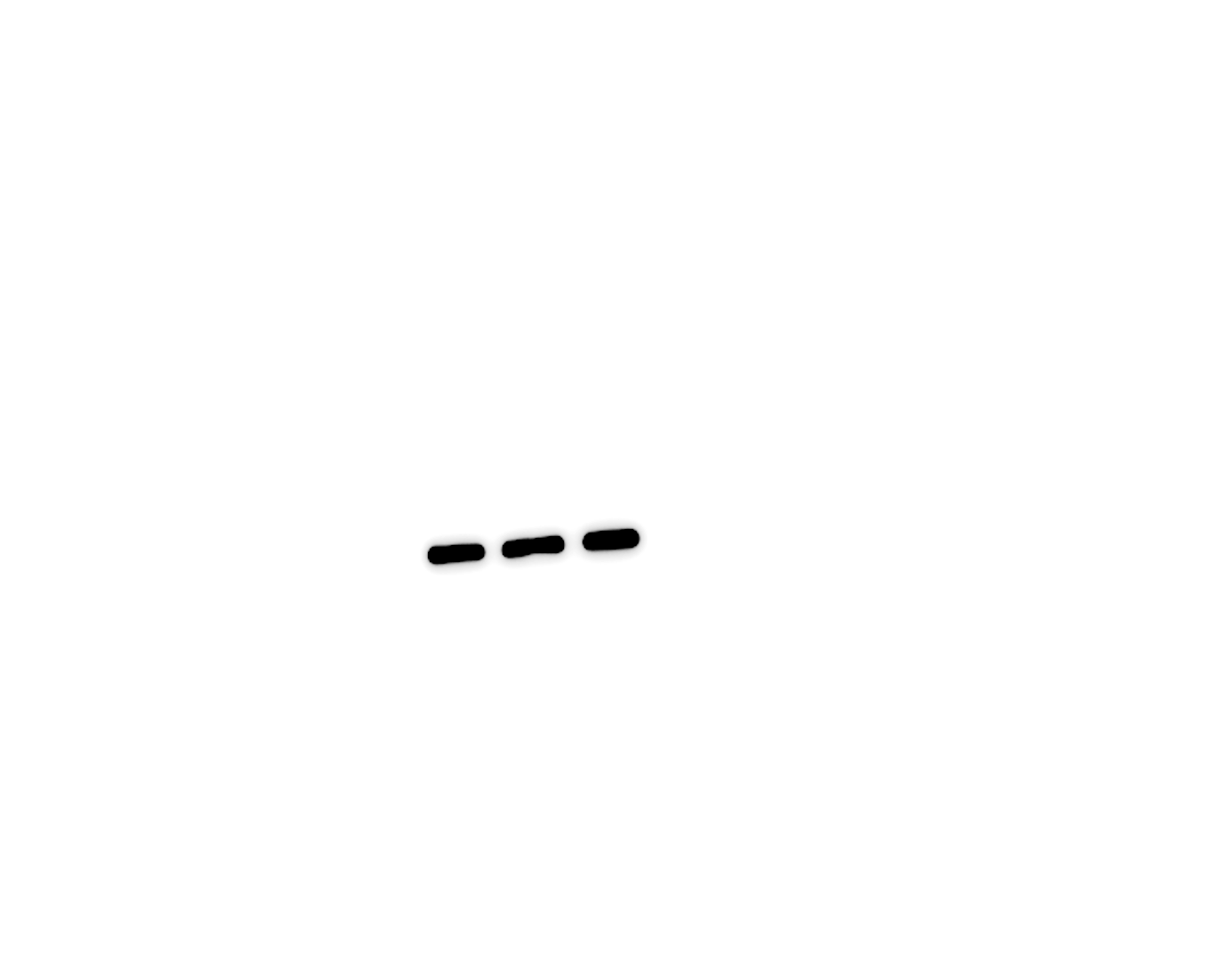

Supplement: DATA SHEET S1 — A full scan of the entire original gel(s). [file Data_Sheet_1.zip › original image files/Figure 6D/T24/GAPDH.Tif]

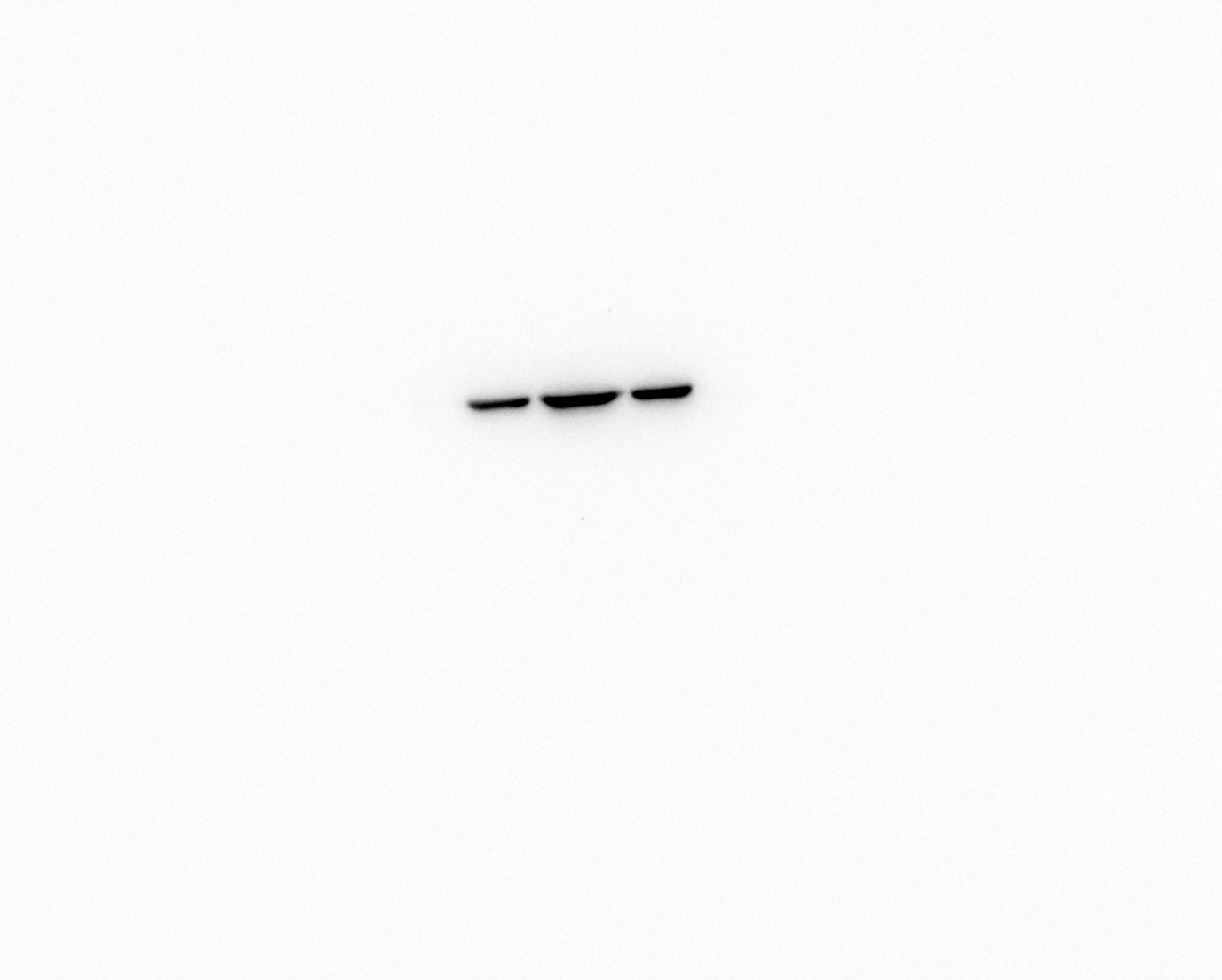

Supplement: DATA SHEET S1 — A full scan of the entire original gel(s). [file Data_Sheet_1.zip › original image files/Figure 6D/T24/JAK2.Tif]

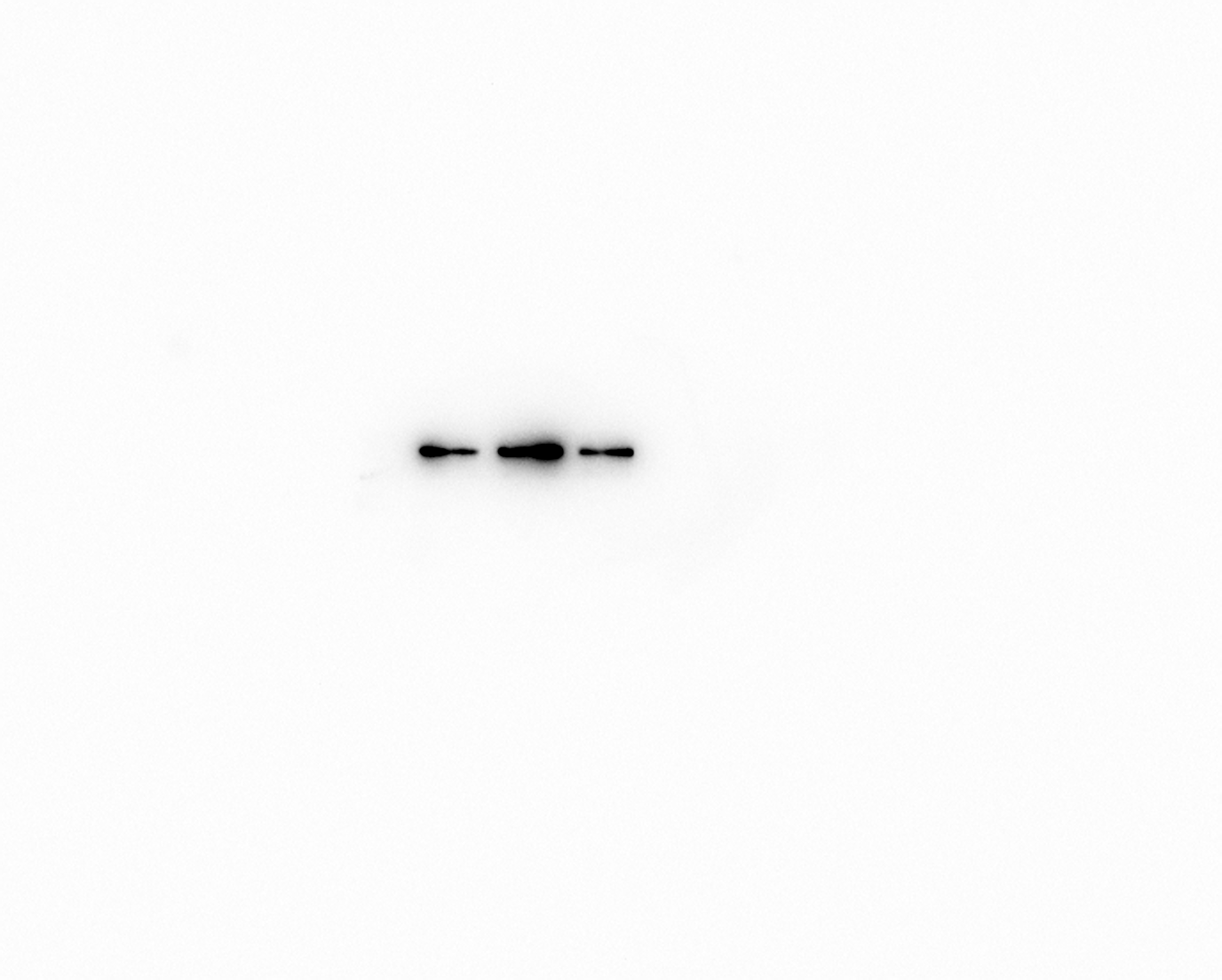

Supplement: DATA SHEET S1 — A full scan of the entire original gel(s). [file Data_Sheet_1.zip › original image files/Figure 6D/T24/P-JAK2.Tif]

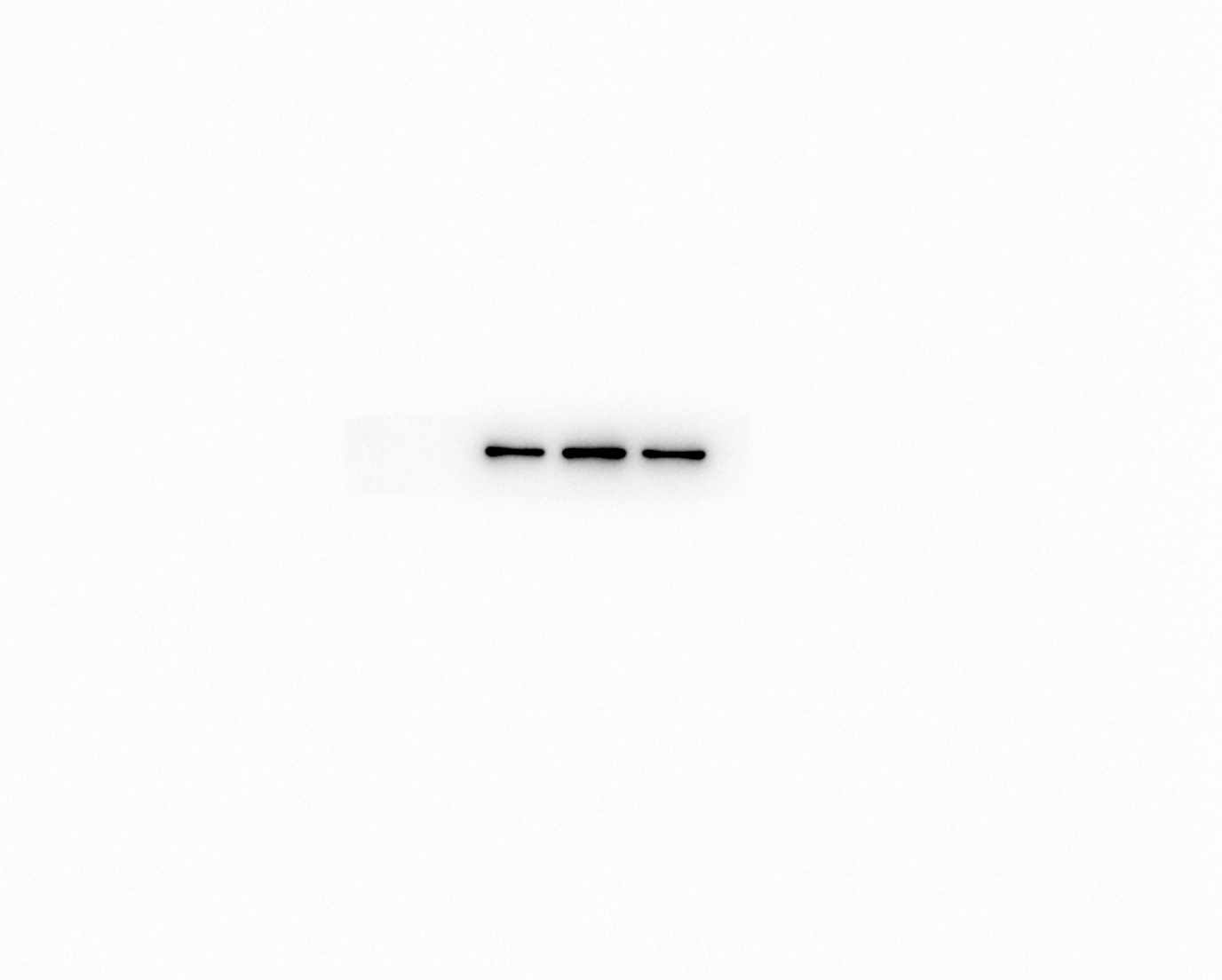

Supplement: DATA SHEET S1 — A full scan of the entire original gel(s). [file Data_Sheet_1.zip › original image files/Figure 6D/T24/PYCR1.Tif]

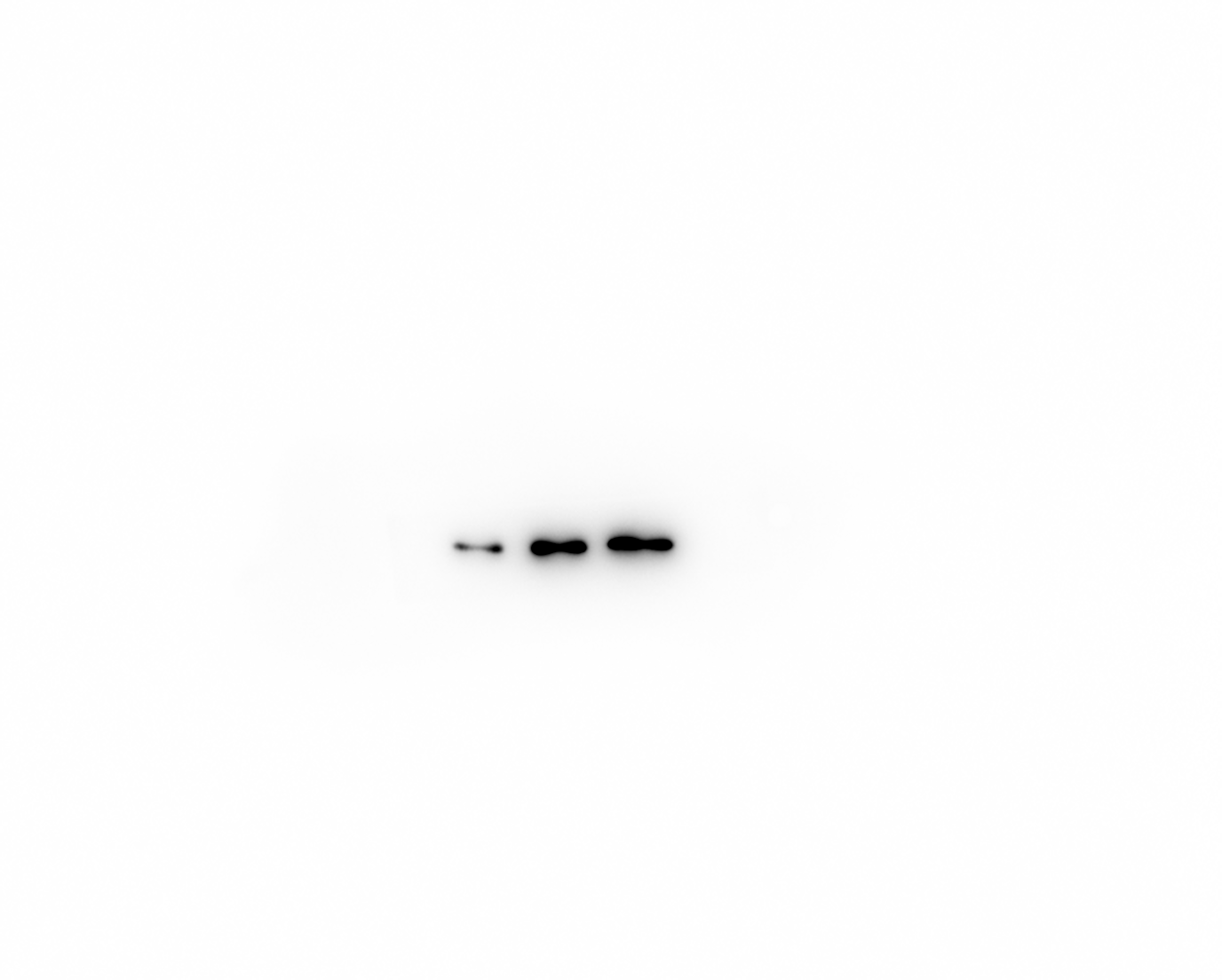

Supplement: DATA SHEET S1 — A full scan of the entire original gel(s). [file Data_Sheet_1.zip › original image files/Figure 6D/T24/RAC3.Tif]

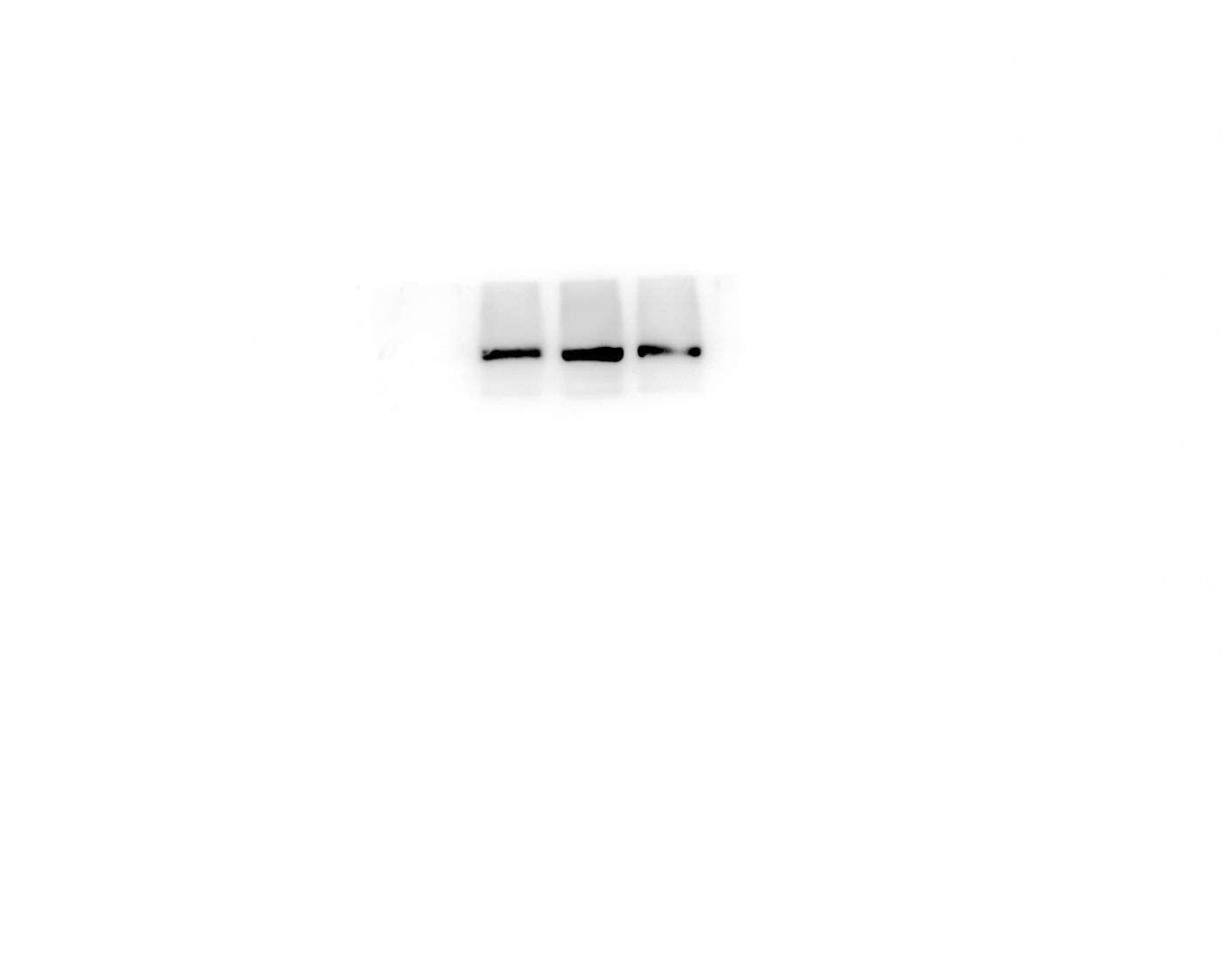

Supplement: DATA SHEET S1 — A full scan of the entire original gel(s). [file Data_Sheet_1.zip › original image files/Figure 6D/T24/STAT3.Tif]

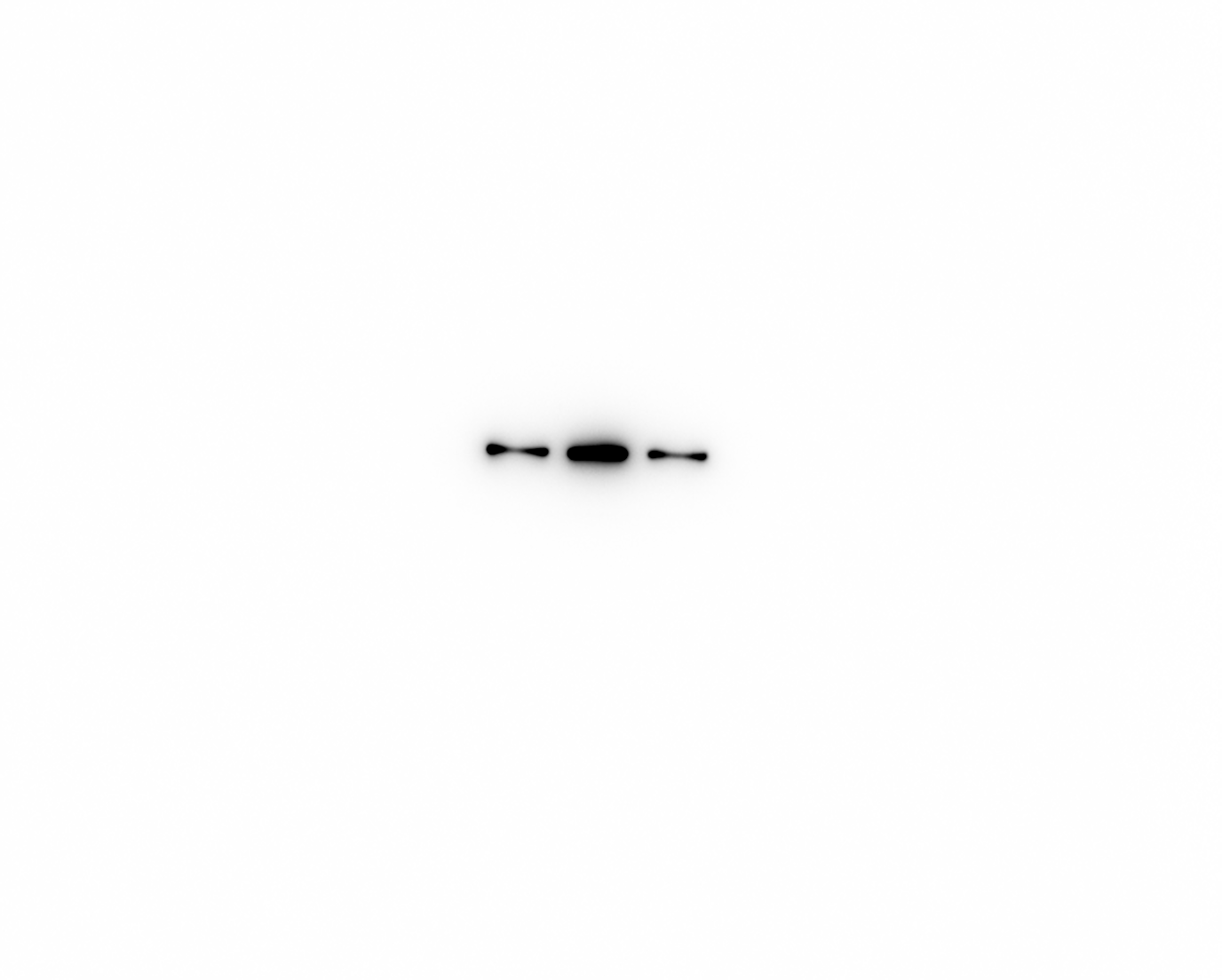

Supplement: DATA SHEET S1 — A full scan of the entire original gel(s). [file Data_Sheet_1.zip › original image files/Figure 6D/T24/c-Myc.Tif]

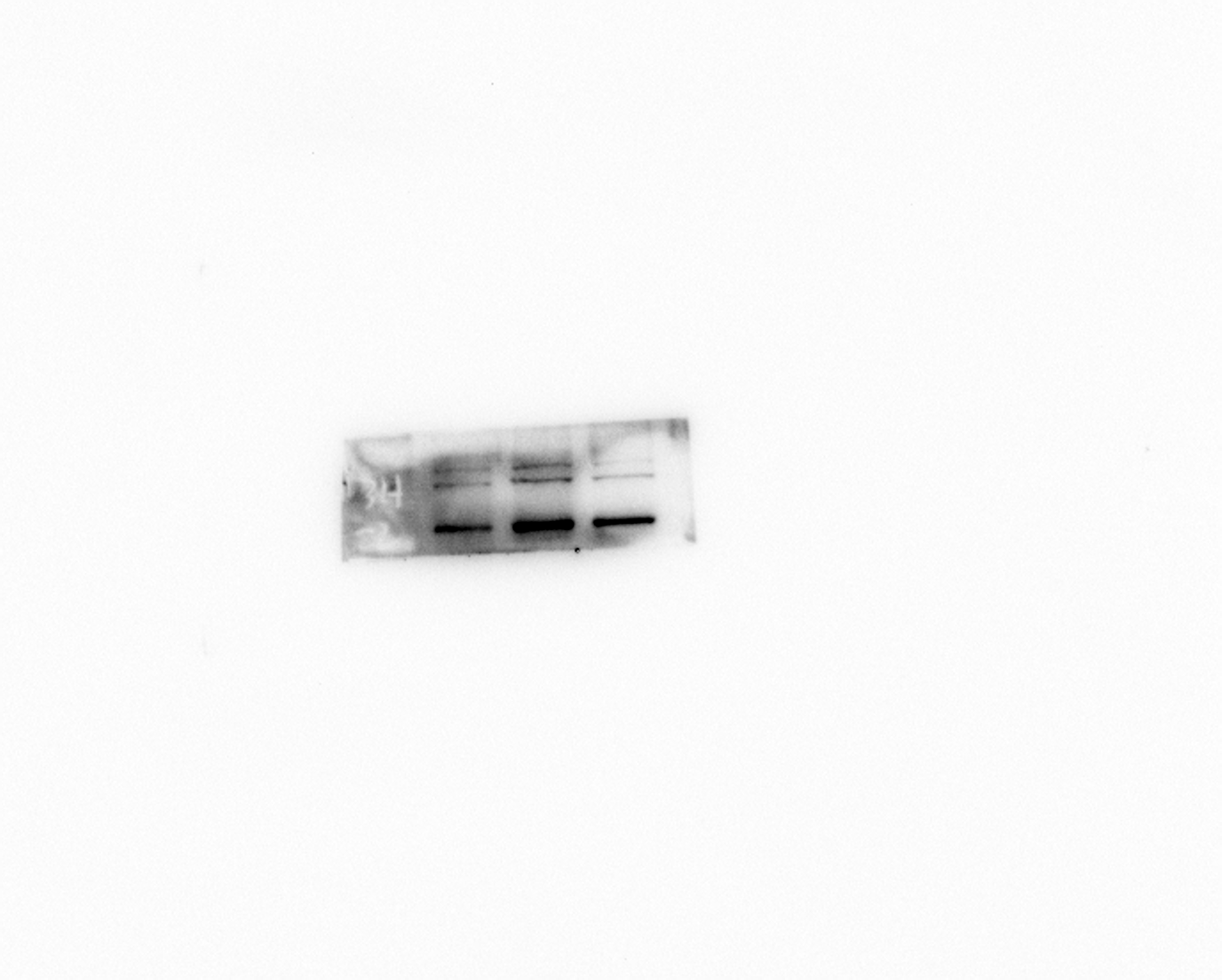

Supplement: DATA SHEET S1 — A full scan of the entire original gel(s). [file Data_Sheet_1.zip › original image files/Figure 6D/T24/p-STAT3.Tif]

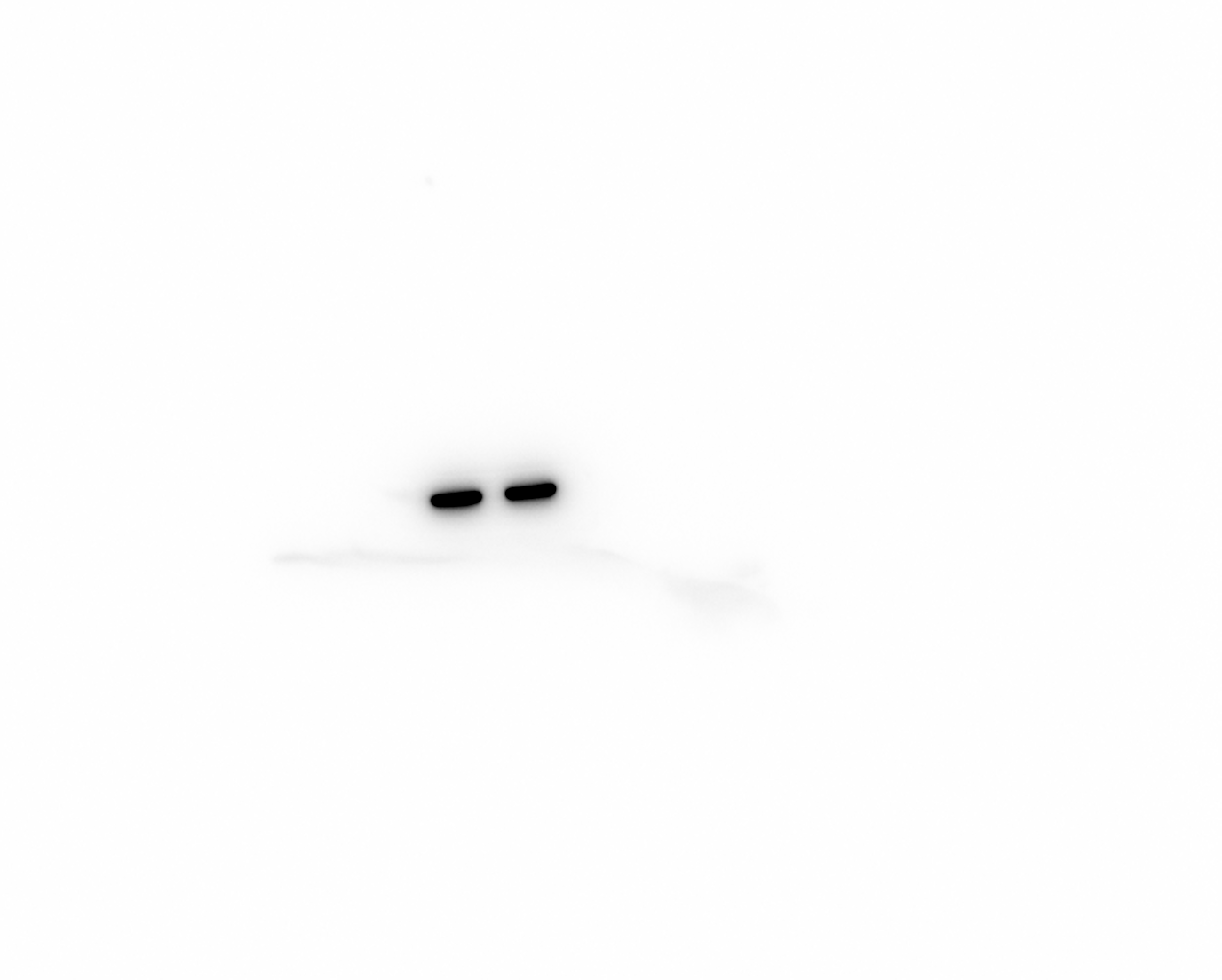

Supplement: DATA SHEET S1 — A full scan of the entire original gel(s). [file Data_Sheet_1.zip › original image files/Figure S1/J82/GAPDH.Tif]

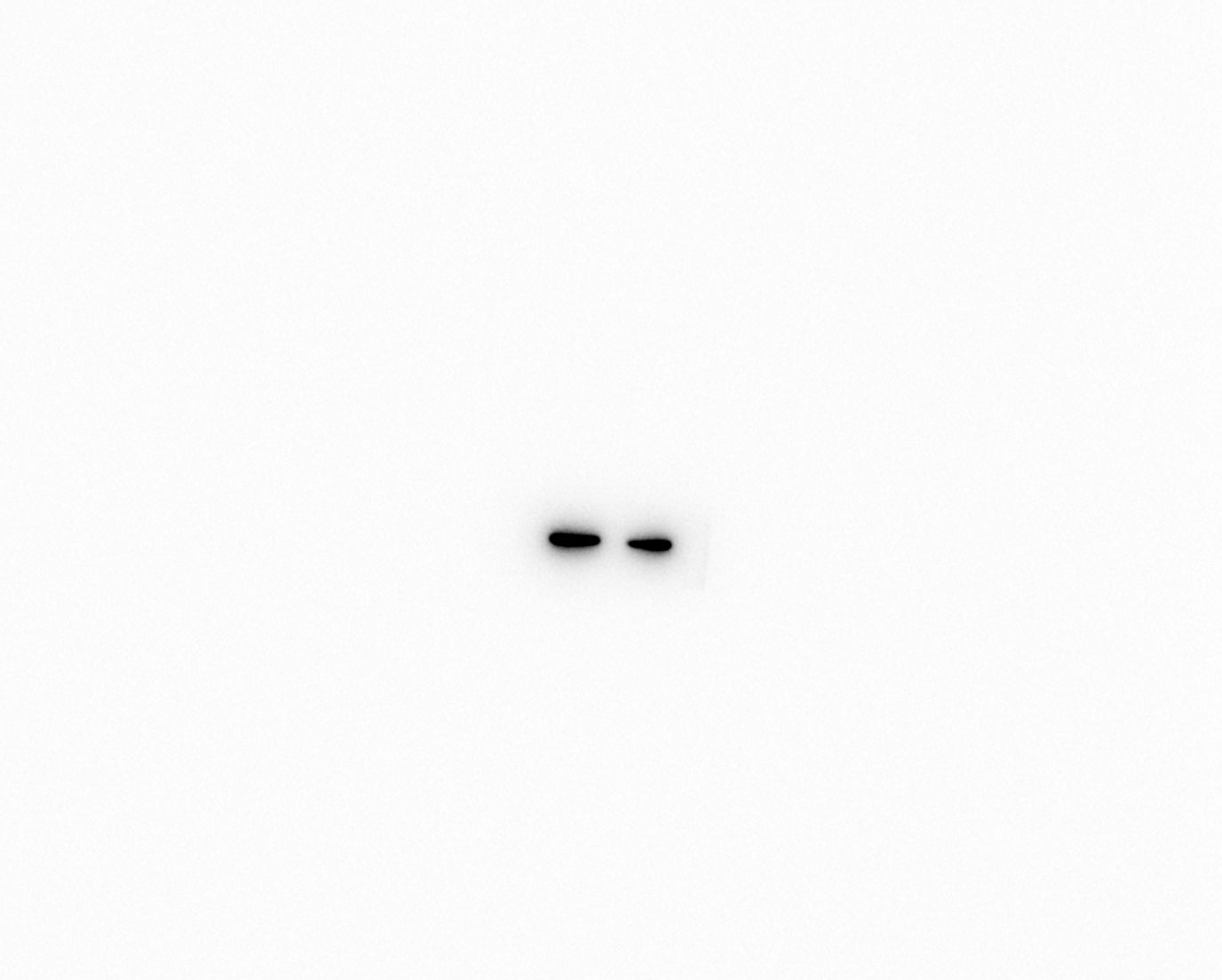

Supplement: DATA SHEET S1 — A full scan of the entire original gel(s). [file Data_Sheet_1.zip › original image files/Figure S1/J82/RAC1.Tif]

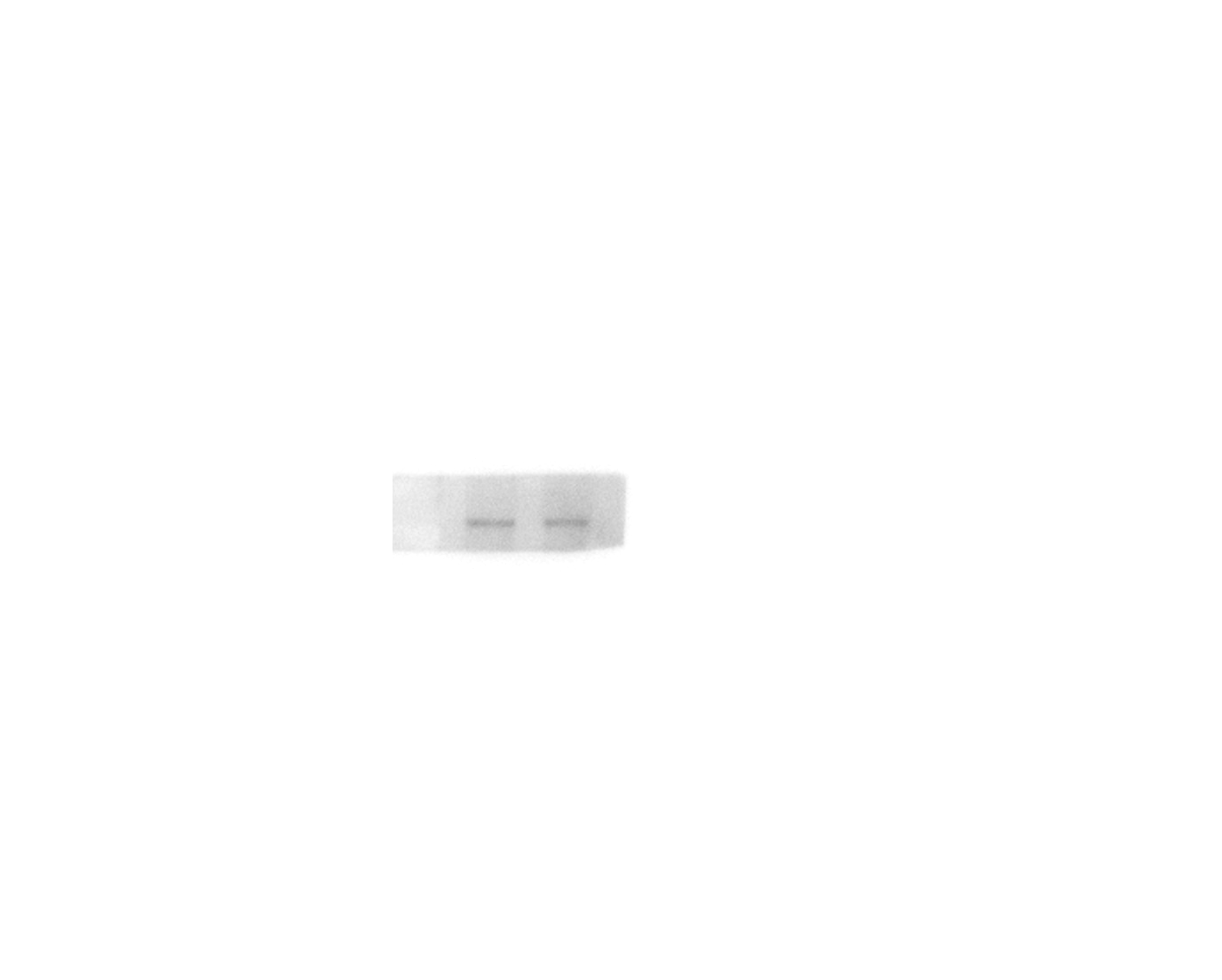

Supplement: DATA SHEET S1 — A full scan of the entire original gel(s). [file Data_Sheet_1.zip › original image files/Figure S1/J82/RAC2.tif]

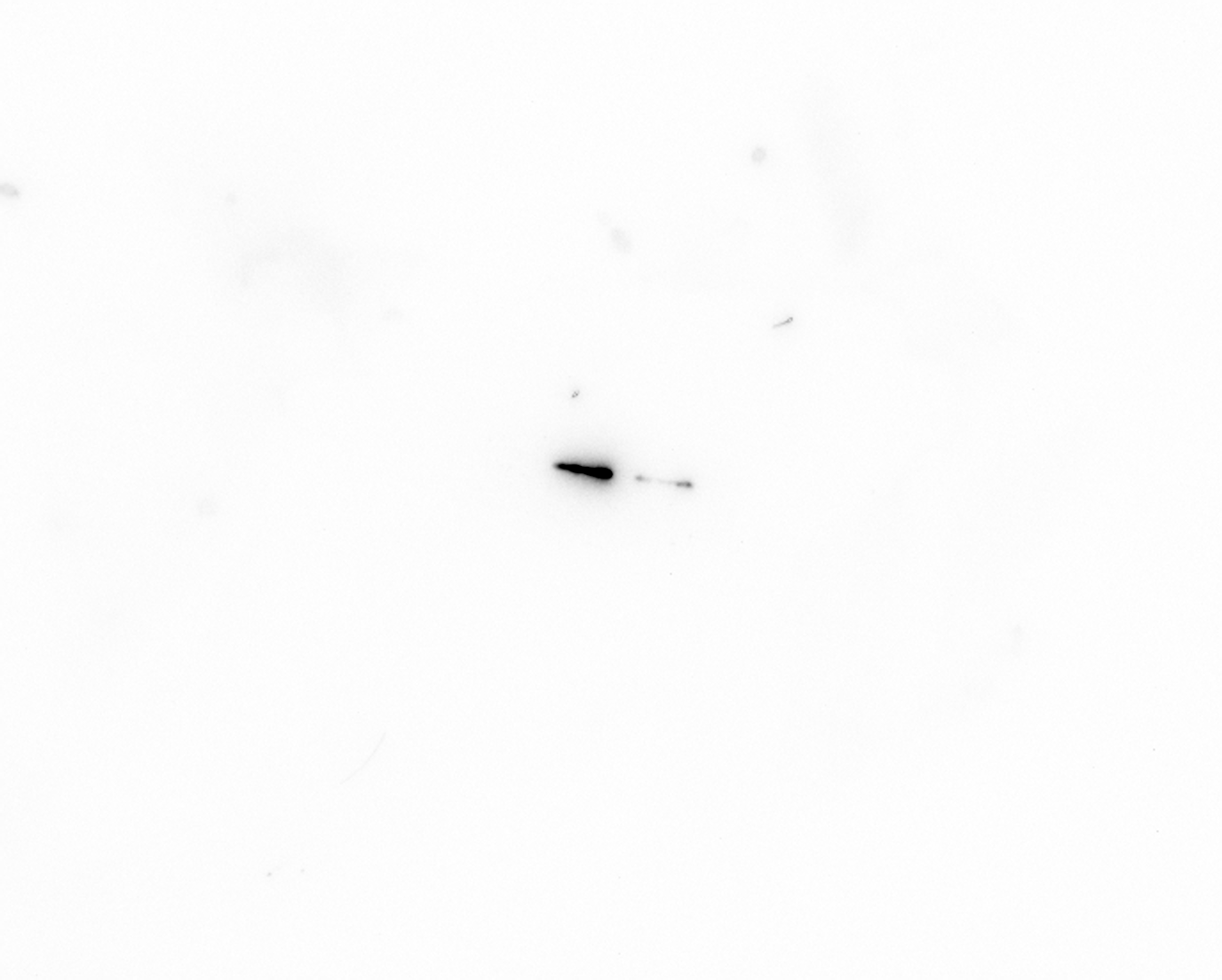

Supplement: DATA SHEET S1 — A full scan of the entire original gel(s). [file Data_Sheet_1.zip › original image files/Figure S1/J82/RAC3.Tif]

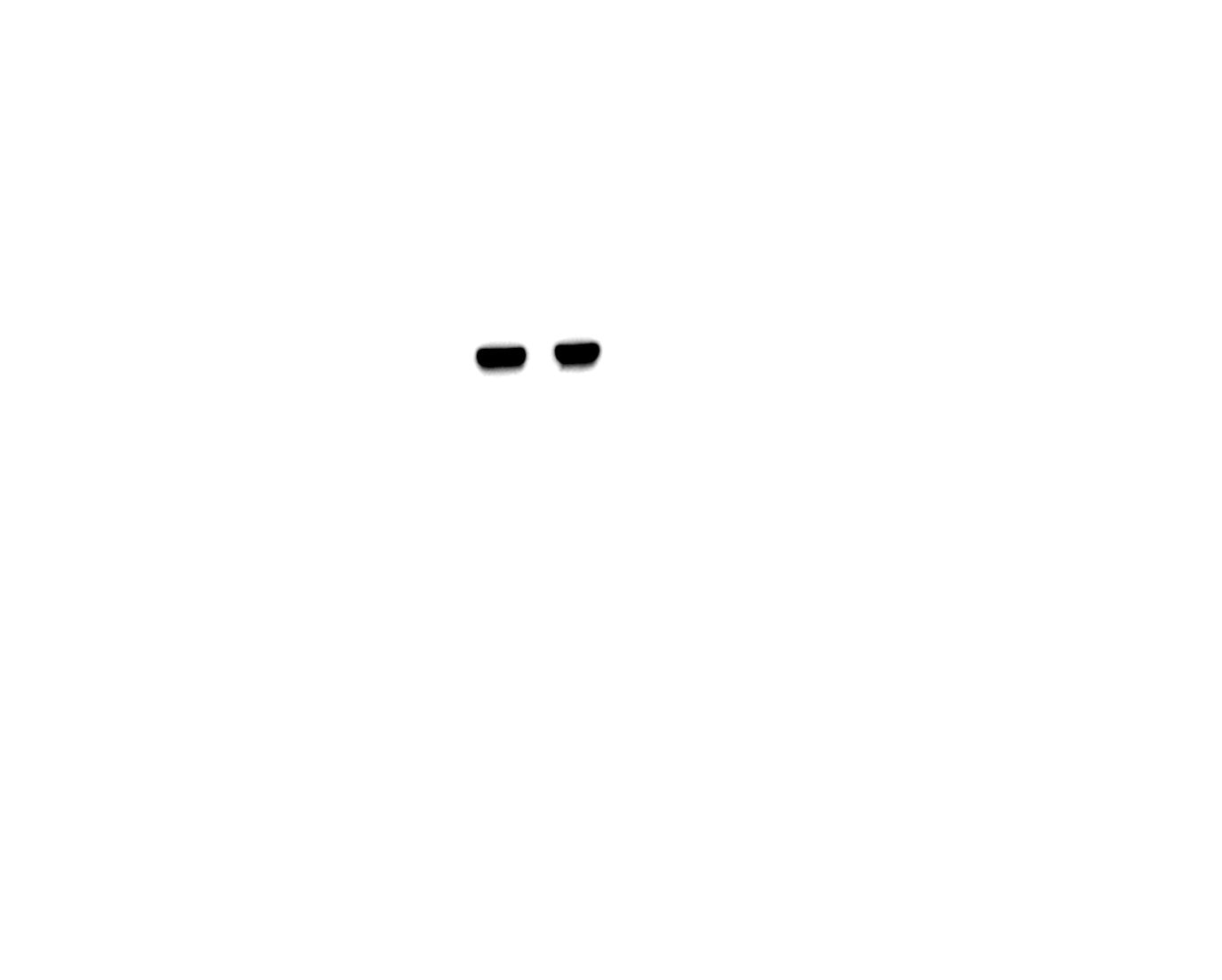

Supplement: DATA SHEET S1 — A full scan of the entire original gel(s). [file Data_Sheet_1.zip › original image files/Figure S1/T24/GAPDH.tif]

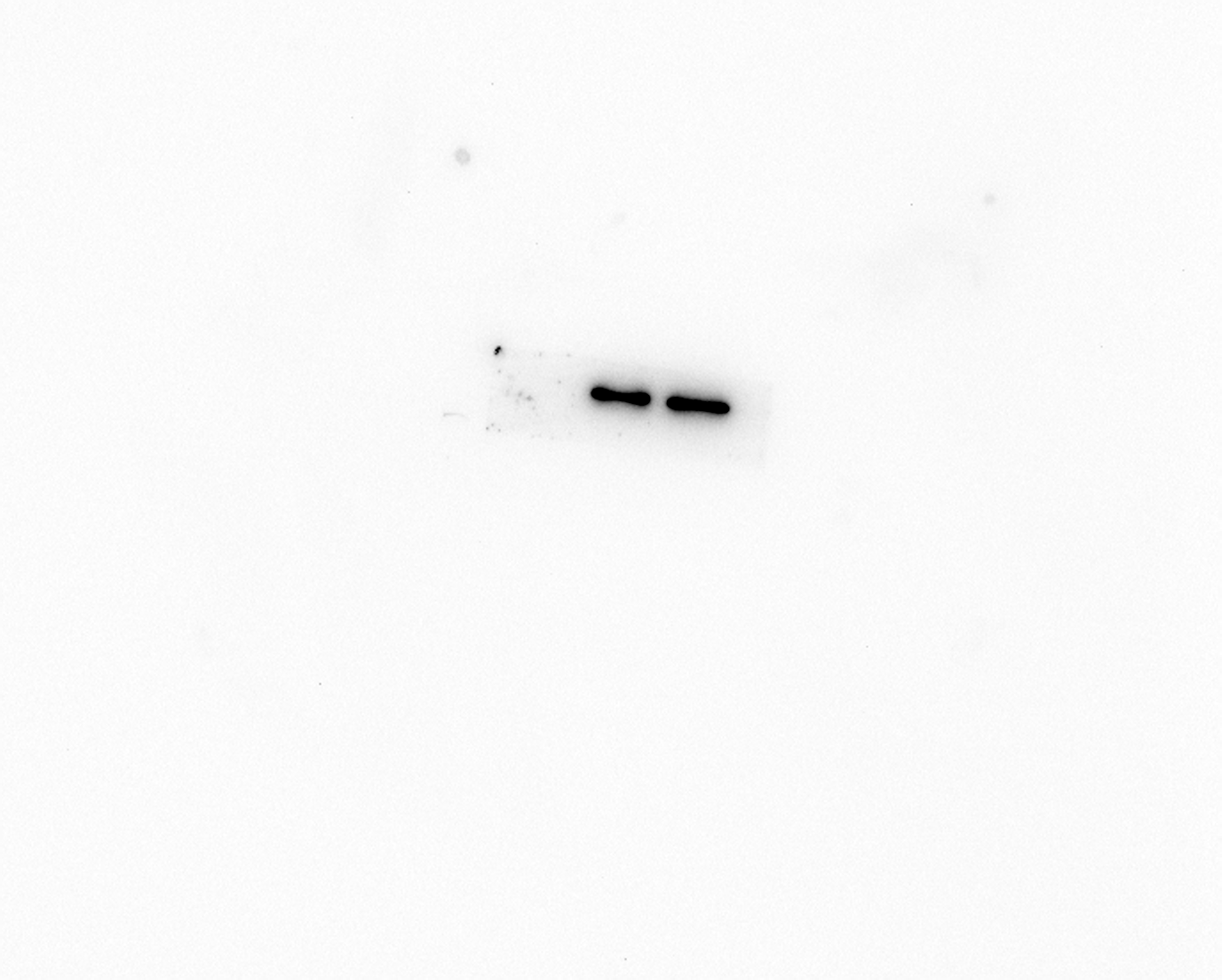

Supplement: DATA SHEET S1 — A full scan of the entire original gel(s). [file Data_Sheet_1.zip › original image files/Figure S1/T24/RAC1.Tif]

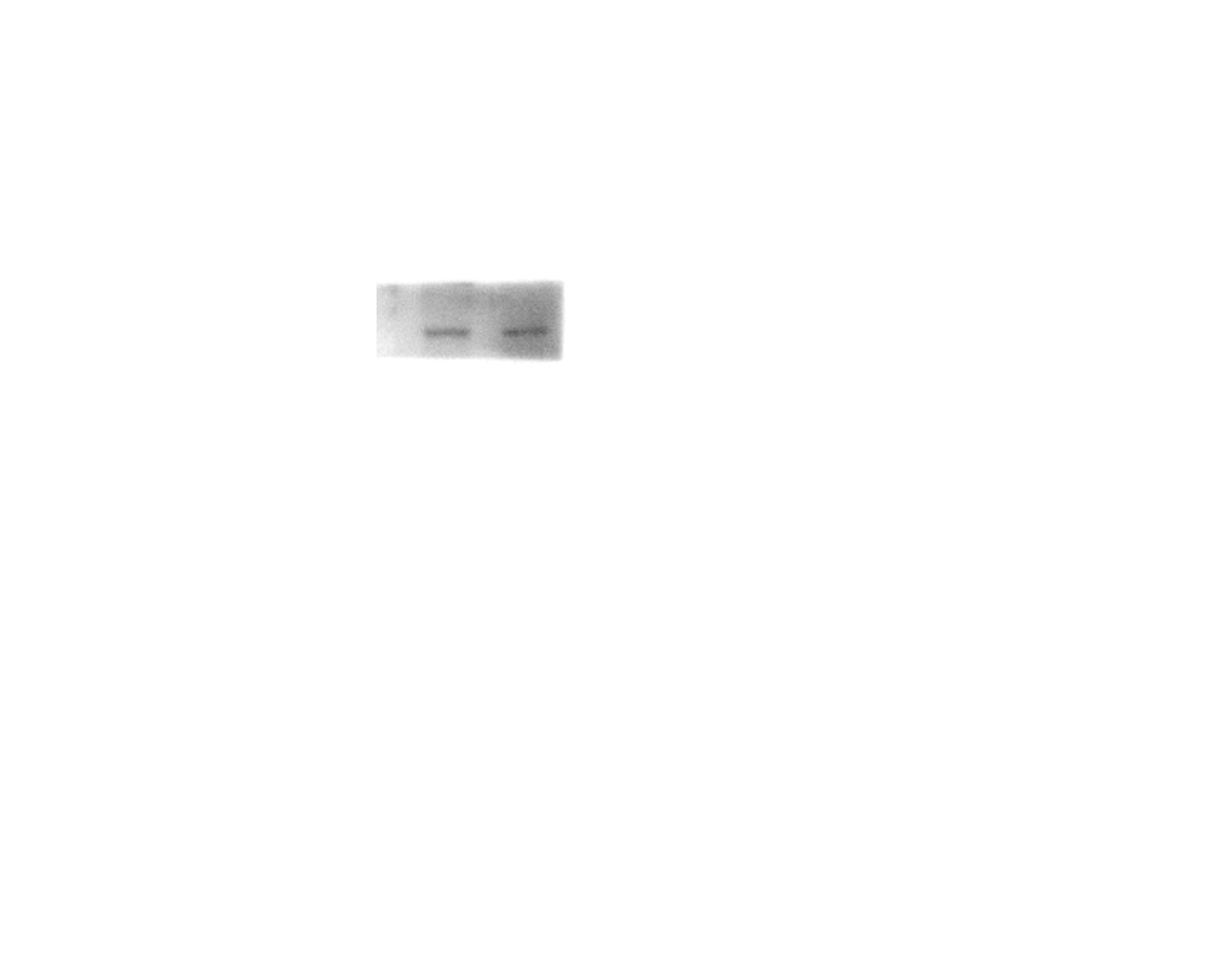

Supplement: DATA SHEET S1 — A full scan of the entire original gel(s). [file Data_Sheet_1.zip › original image files/Figure S1/T24/RAC2.tif]

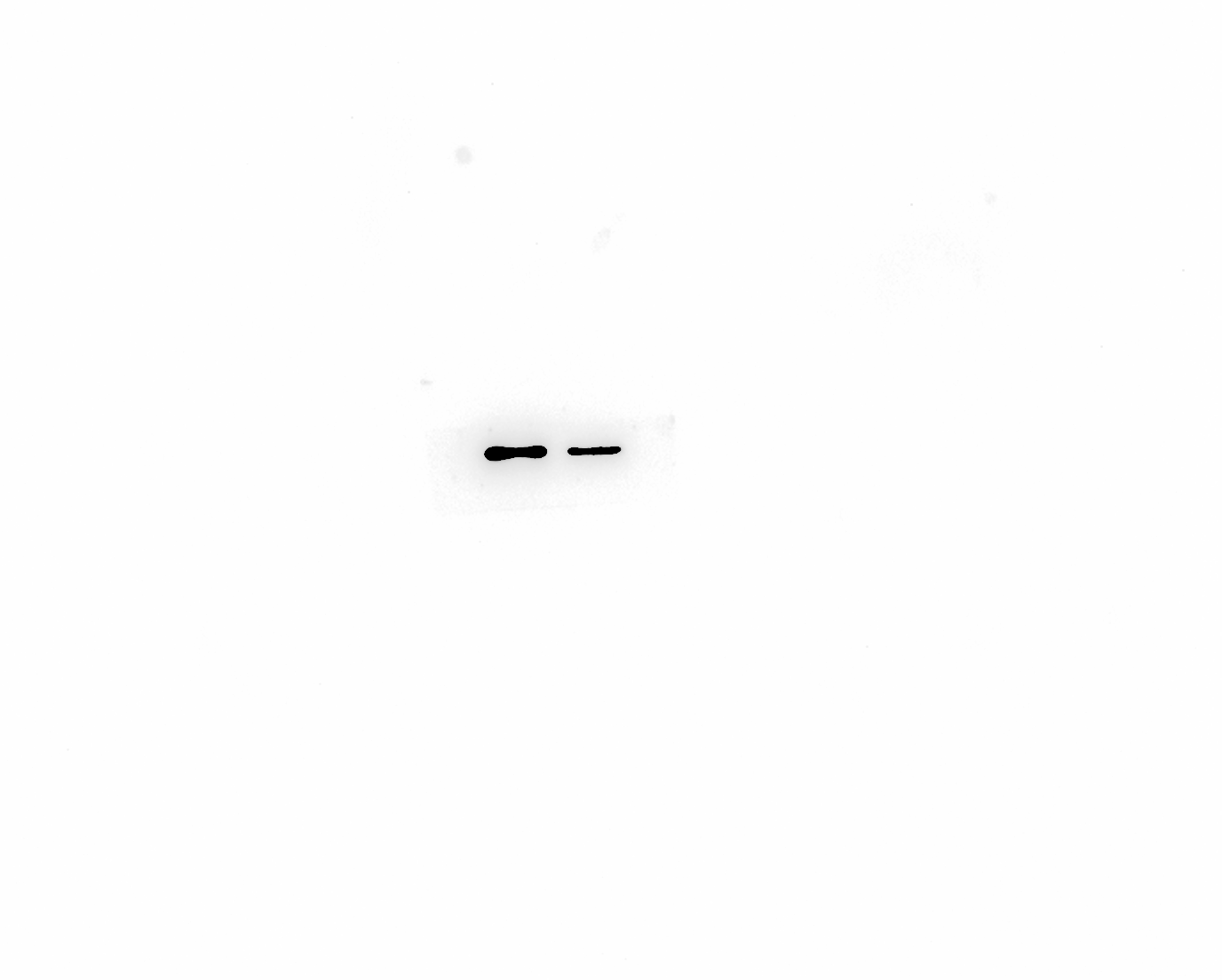

Supplement: DATA SHEET S1 — A full scan of the entire original gel(s). [file Data_Sheet_1.zip › original image files/Figure S1/T24/RAC3.Tif]

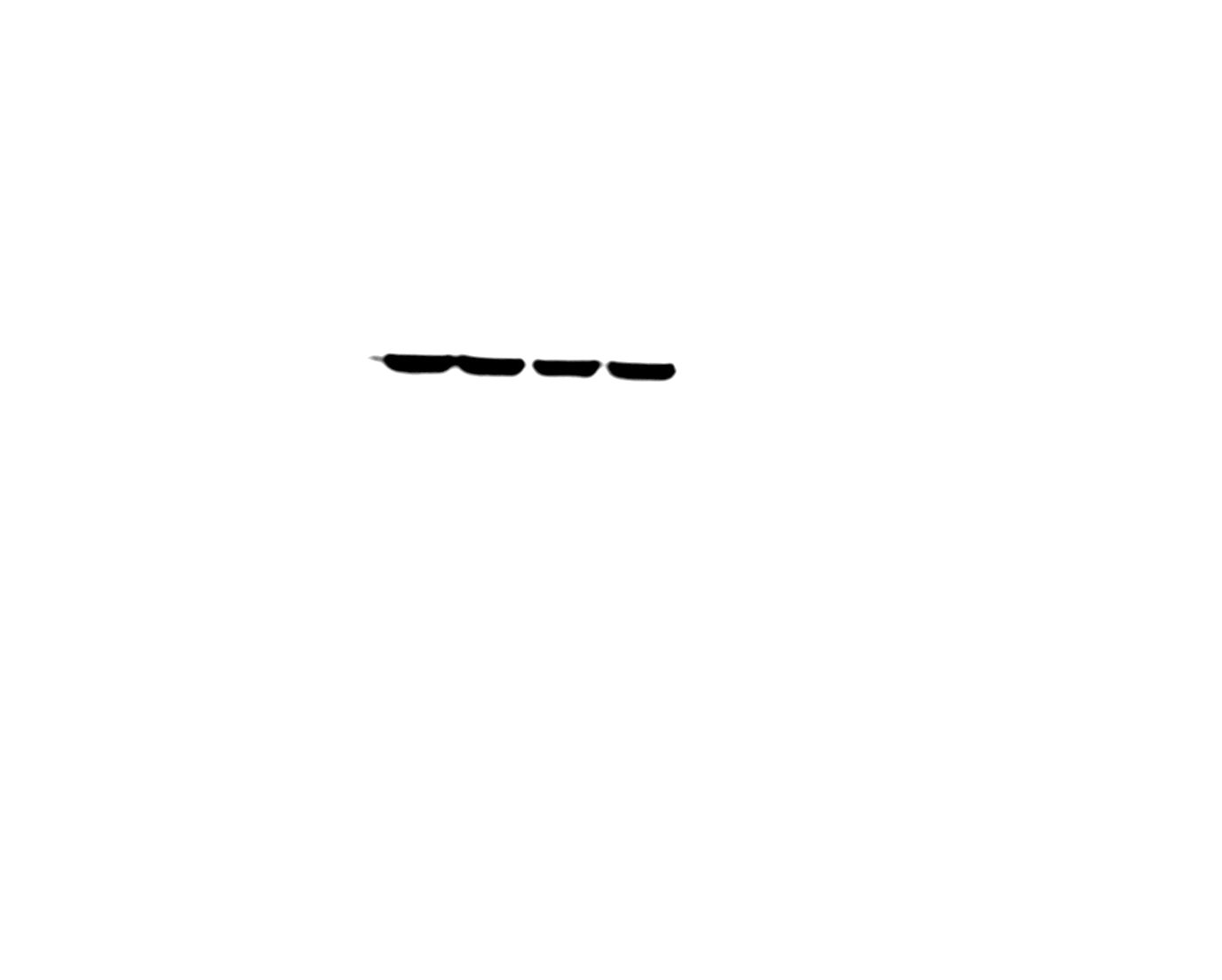

Supplement: DATA SHEET S1 — A full scan of the entire original gel(s). [file Data_Sheet_1.zip › original image files/Figure S2/J82/GAPDH.tif]

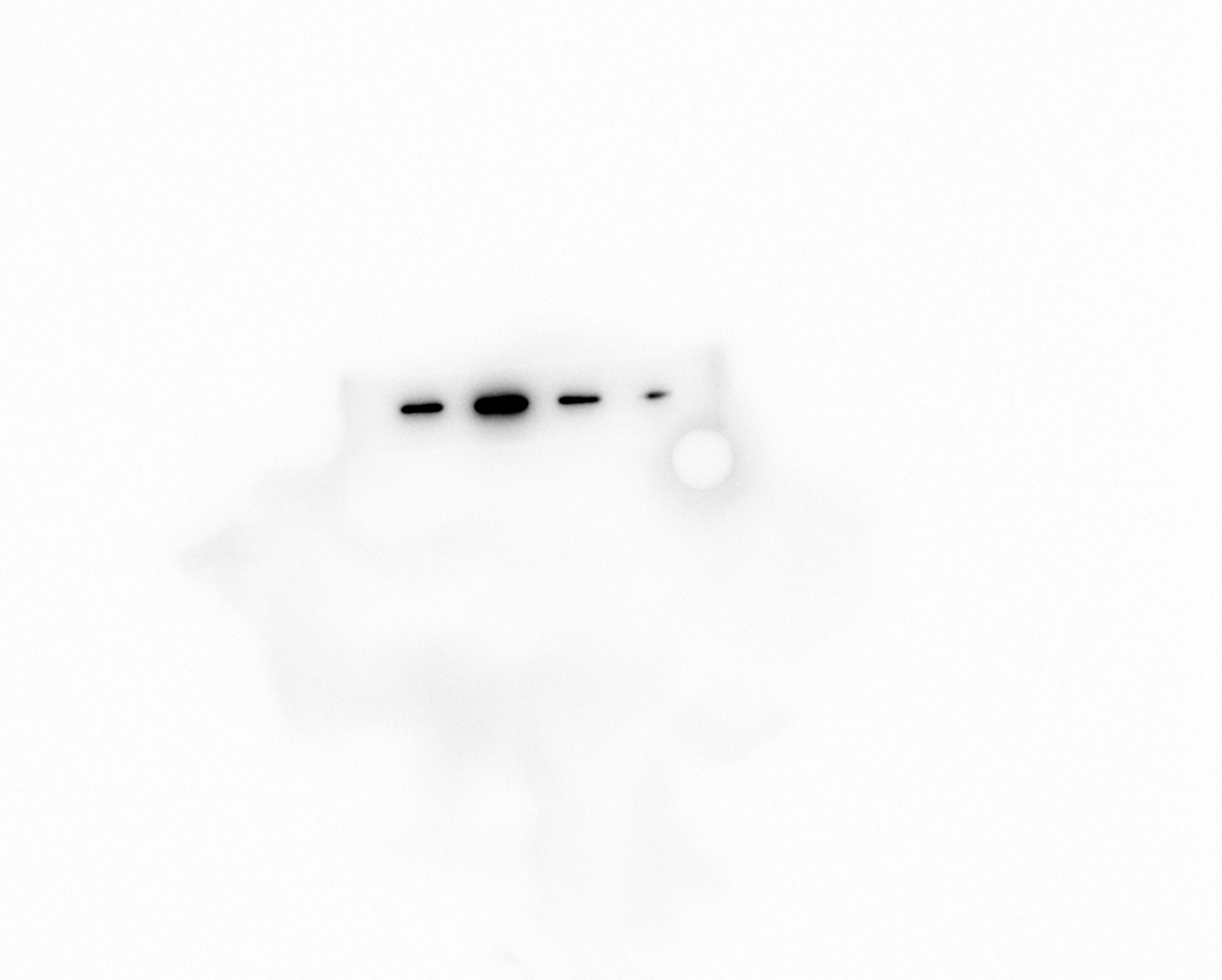

Supplement: DATA SHEET S1 — A full scan of the entire original gel(s). [file Data_Sheet_1.zip › original image files/Figure S2/J82/JAK2.Tif]

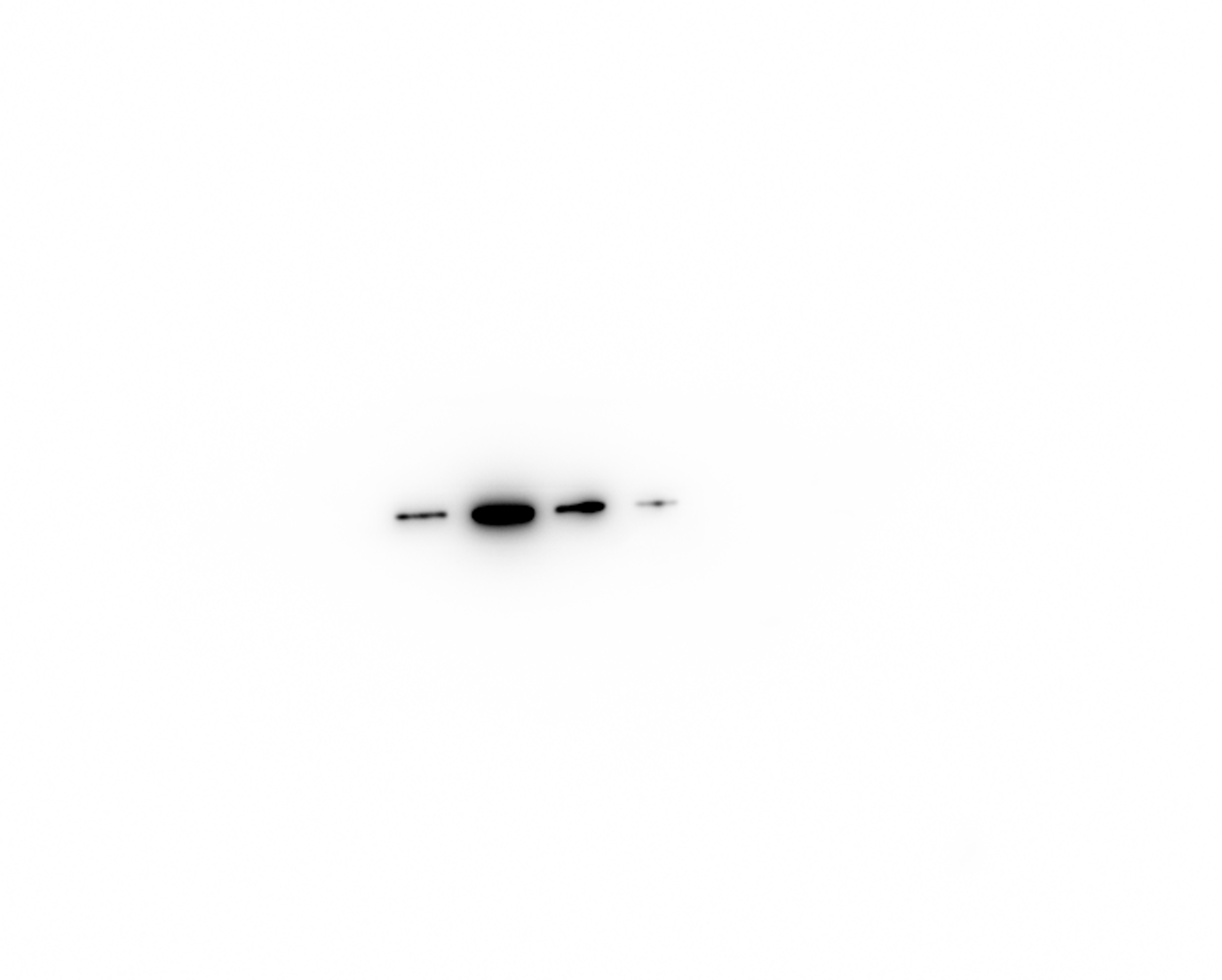

Supplement: DATA SHEET S1 — A full scan of the entire original gel(s). [file Data_Sheet_1.zip › original image files/Figure S2/J82/PYCR1.Tif]

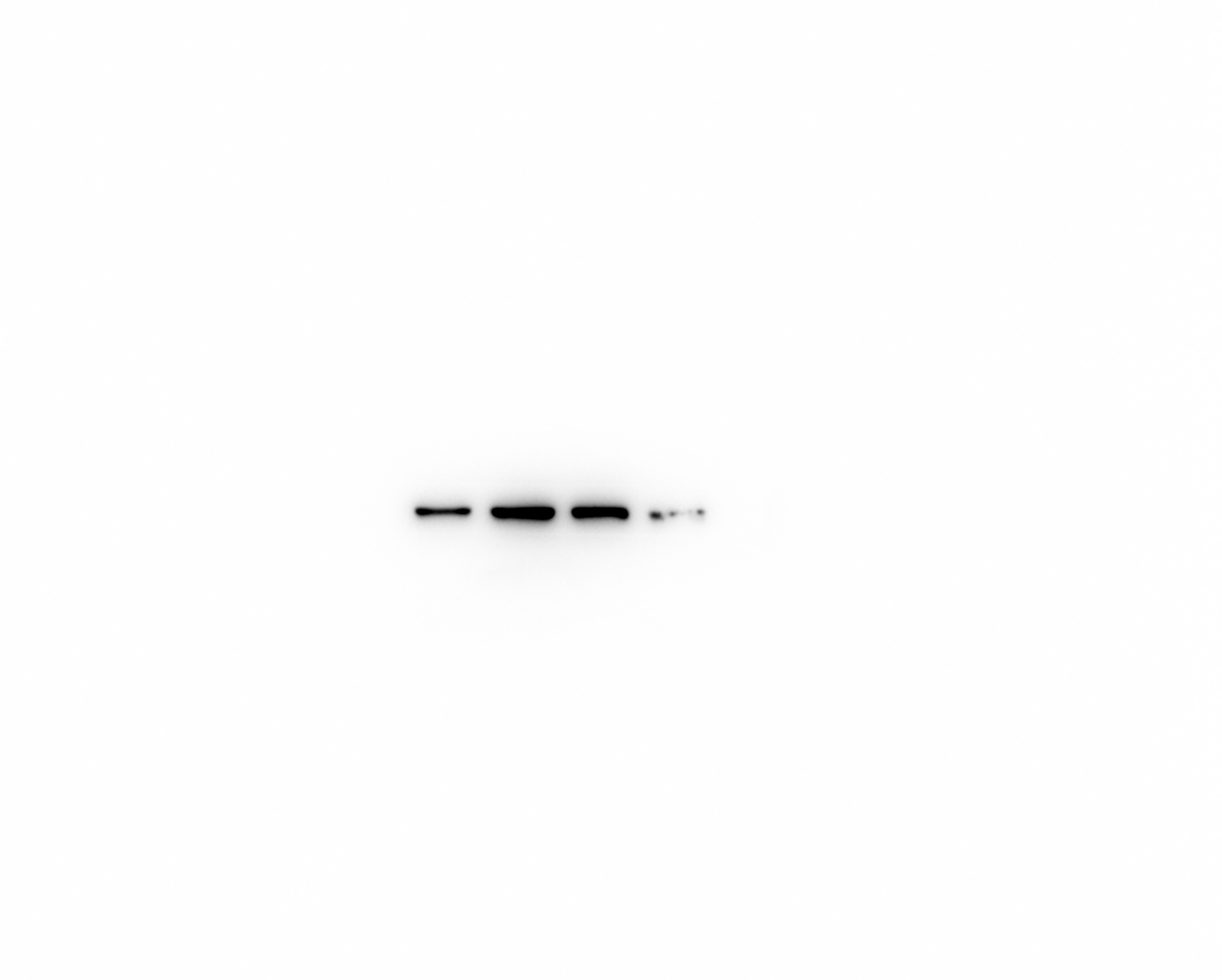

Supplement: DATA SHEET S1 — A full scan of the entire original gel(s). [file Data_Sheet_1.zip › original image files/Figure S2/J82/STAT3.Tif]

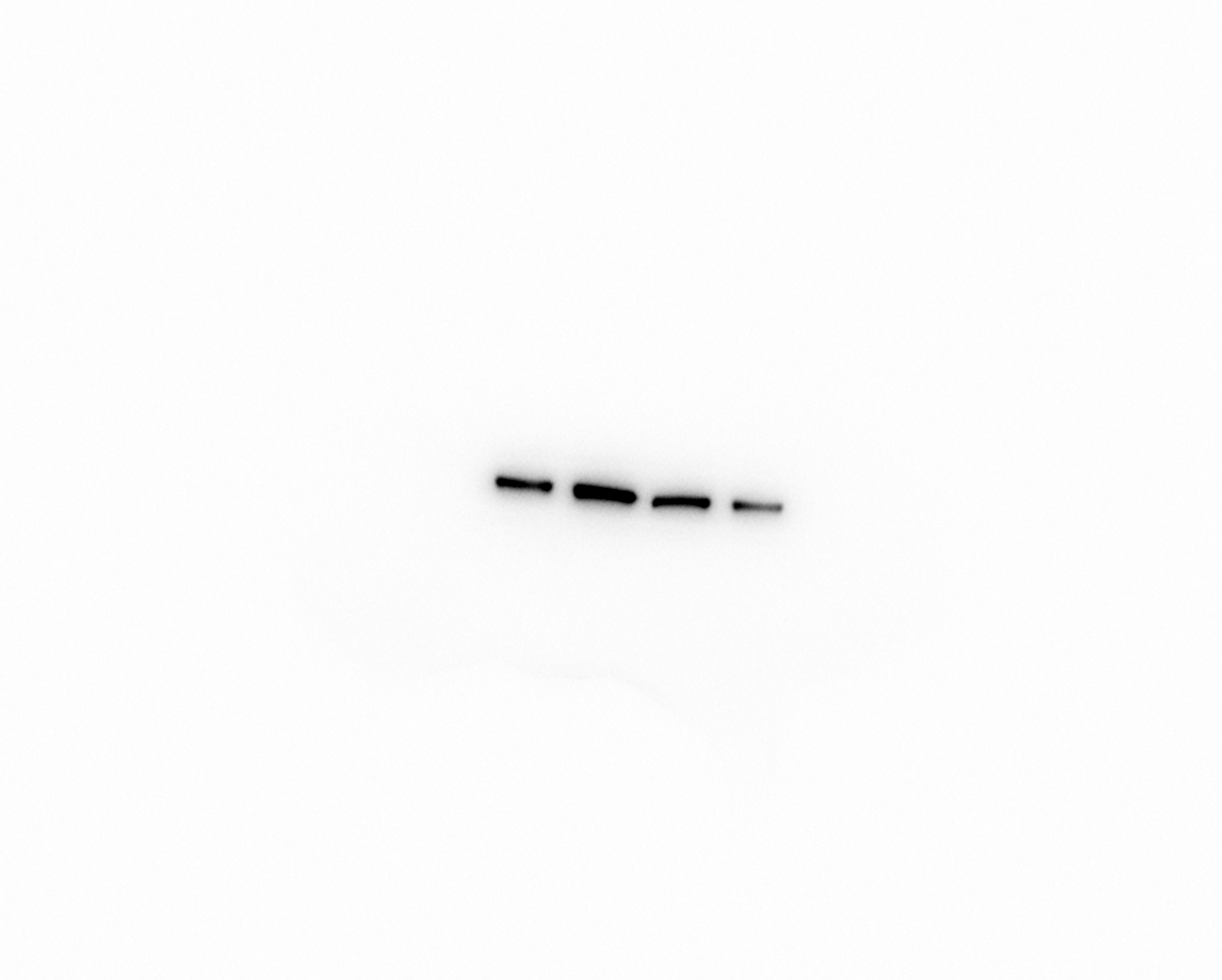

Supplement: DATA SHEET S1 — A full scan of the entire original gel(s). [file Data_Sheet_1.zip › original image files/Figure S2/J82/c-Myc.Tif]

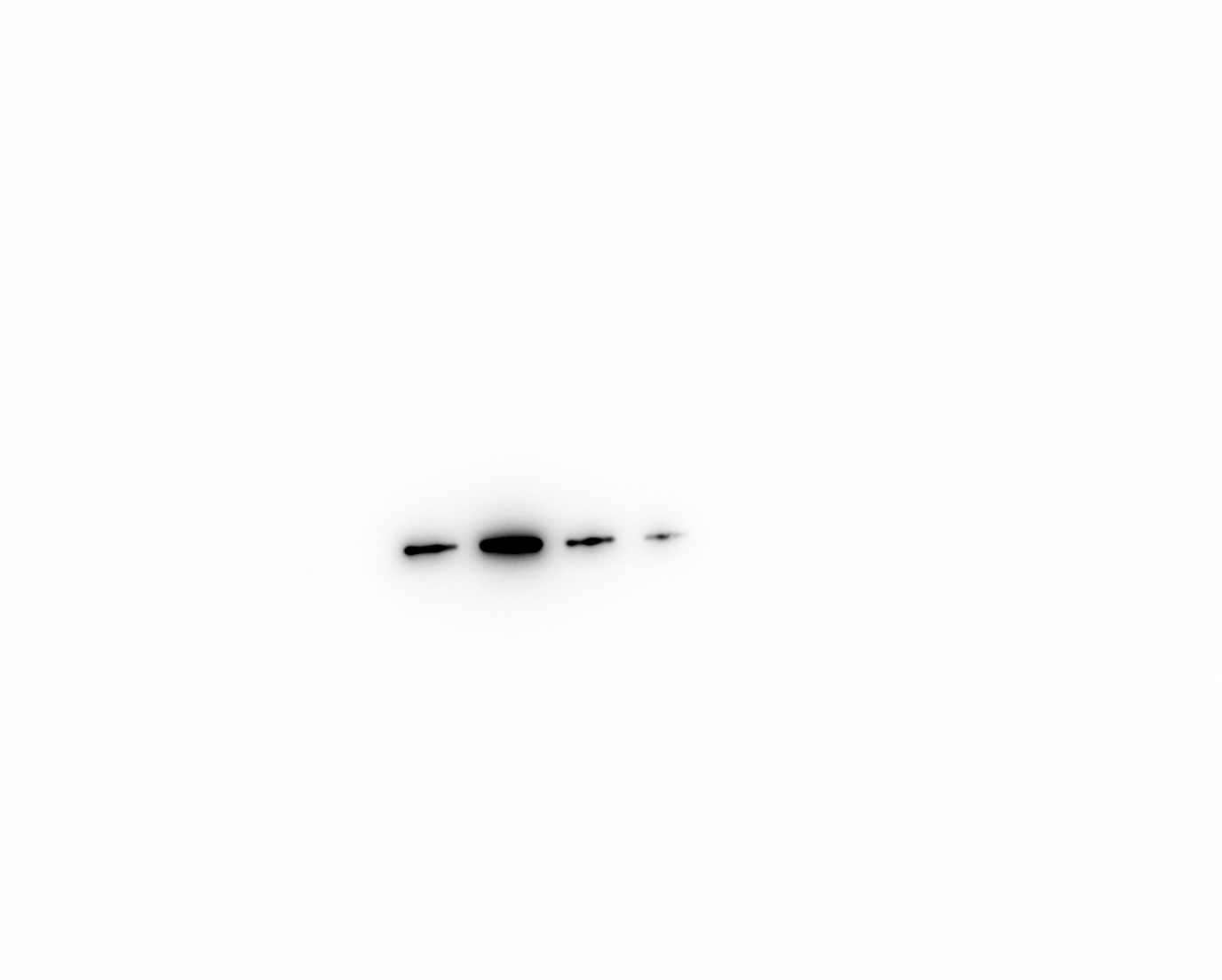

Supplement: DATA SHEET S1 — A full scan of the entire original gel(s). [file Data_Sheet_1.zip › original image files/Figure S2/J82/p-JAK2.Tif]

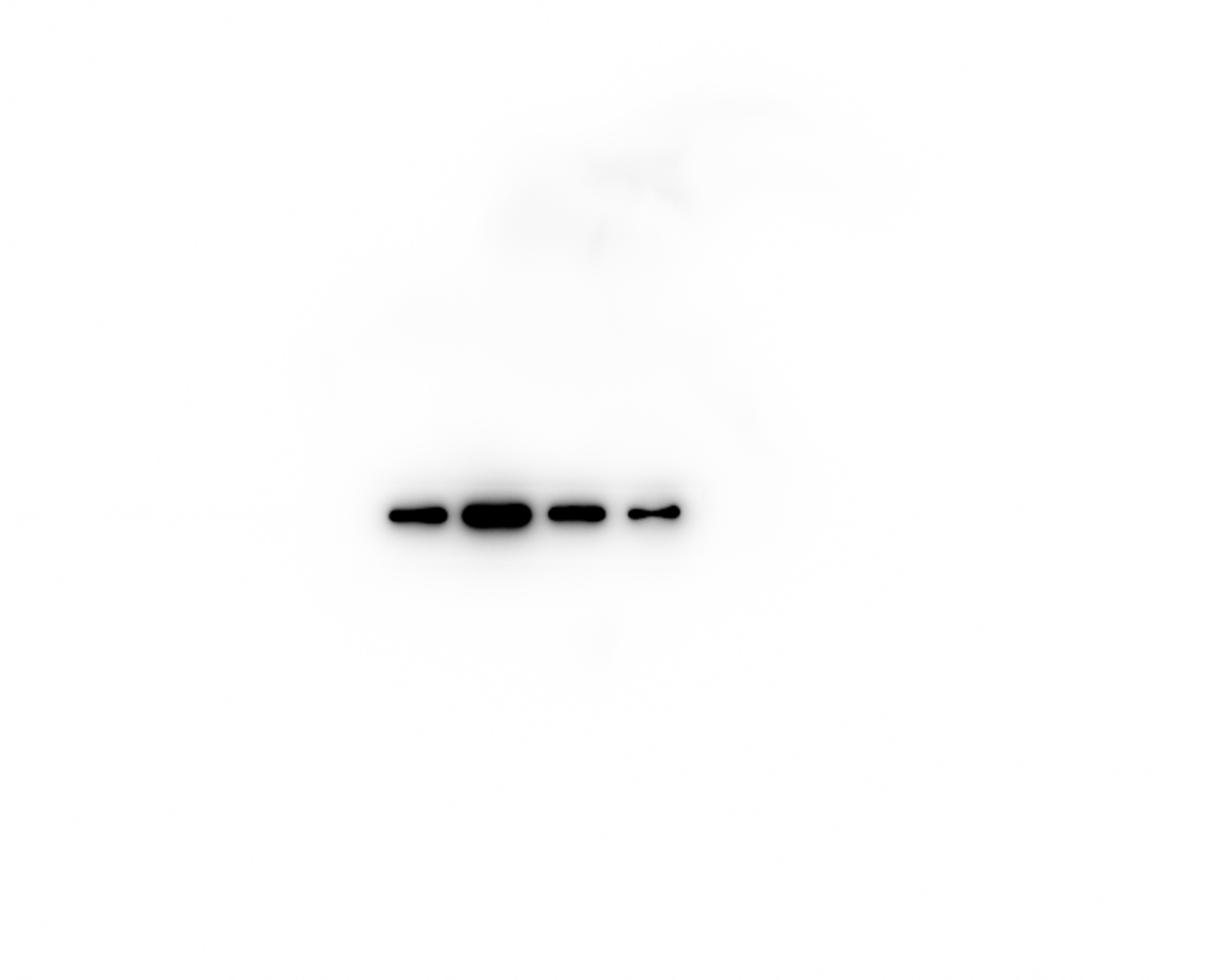

Supplement: DATA SHEET S1 — A full scan of the entire original gel(s). [file Data_Sheet_1.zip › original image files/Figure S2/J82/p-STAT3.Tif]

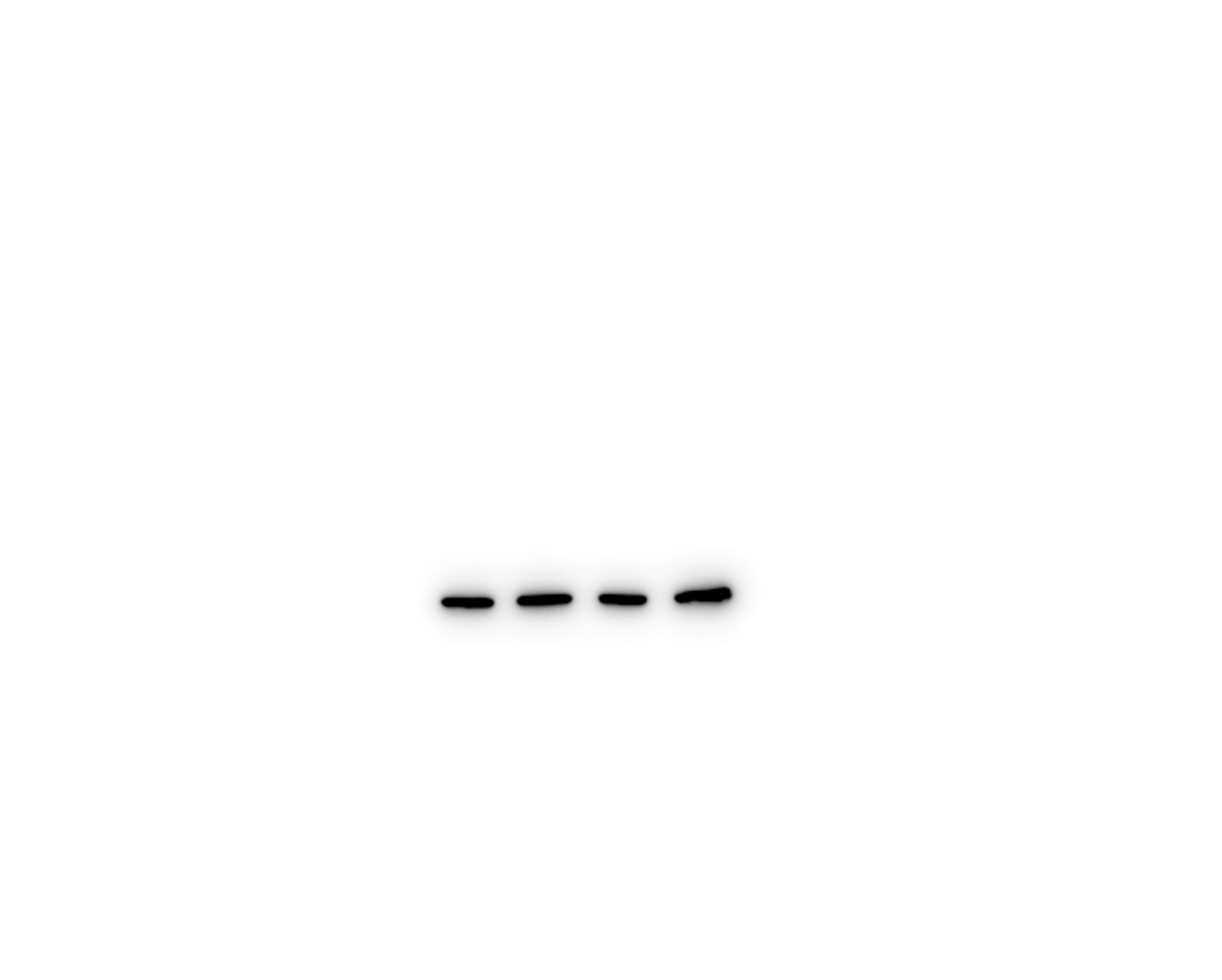

Supplement: DATA SHEET S1 — A full scan of the entire original gel(s). [file Data_Sheet_1.zip › original image files/Figure S2/T24/GAPDH.Tif]

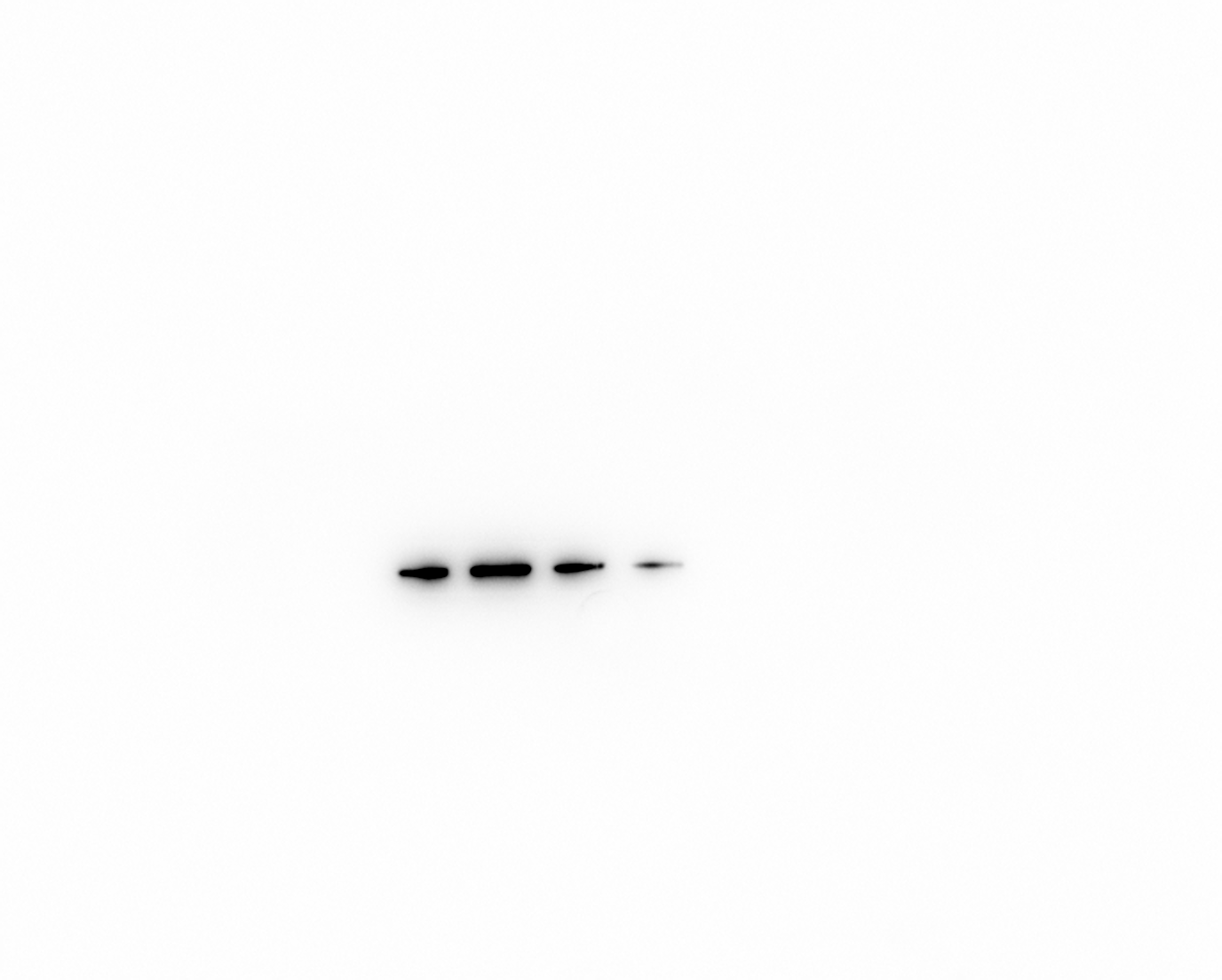

Supplement: DATA SHEET S1 — A full scan of the entire original gel(s). [file Data_Sheet_1.zip › original image files/Figure S2/T24/JAK2.Tif]

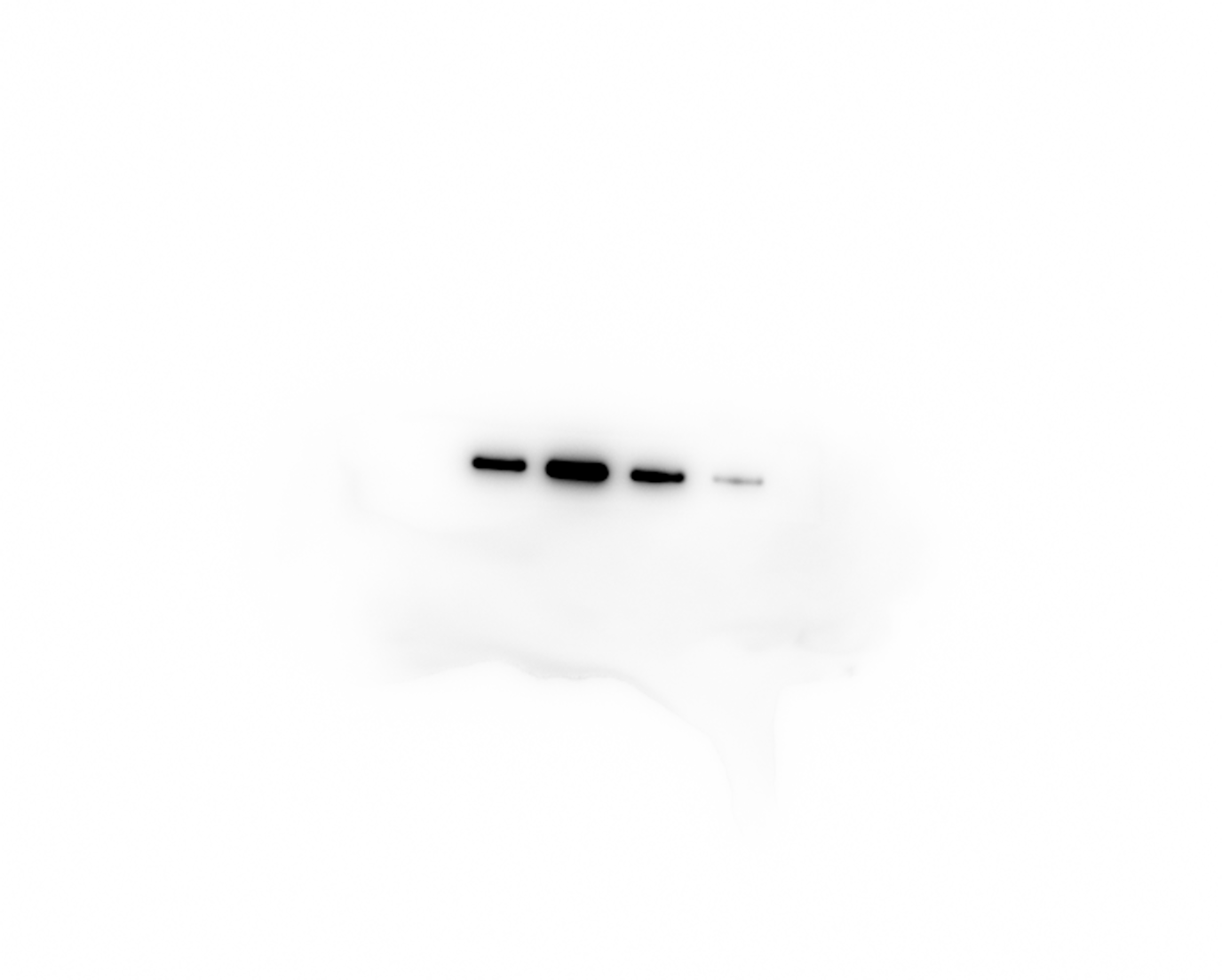

Supplement: DATA SHEET S1 — A full scan of the entire original gel(s). [file Data_Sheet_1.zip › original image files/Figure S2/T24/PYCR1.Tif]

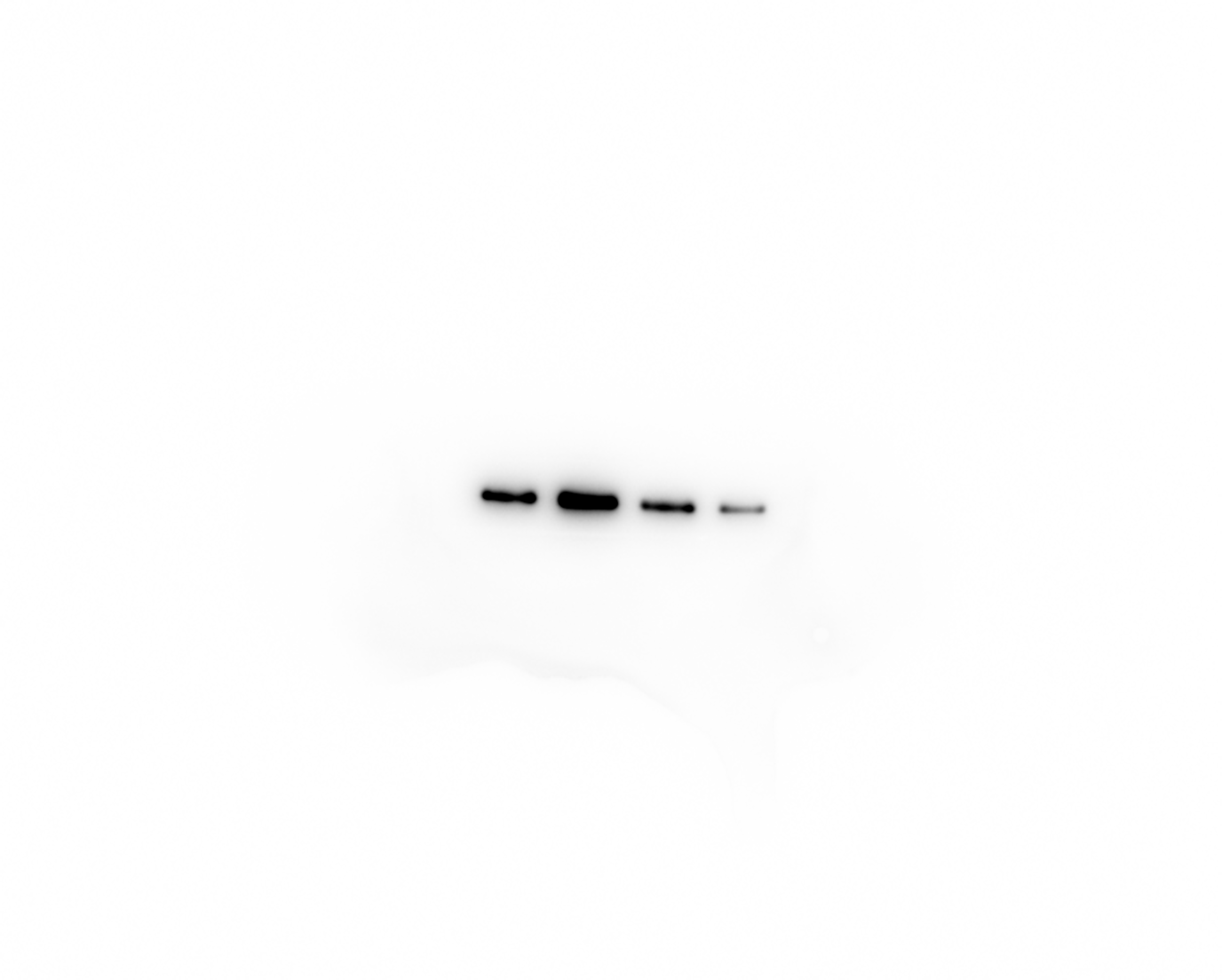

Supplement: DATA SHEET S1 — A full scan of the entire original gel(s). [file Data_Sheet_1.zip › original image files/Figure S2/T24/STAT3.Tif]

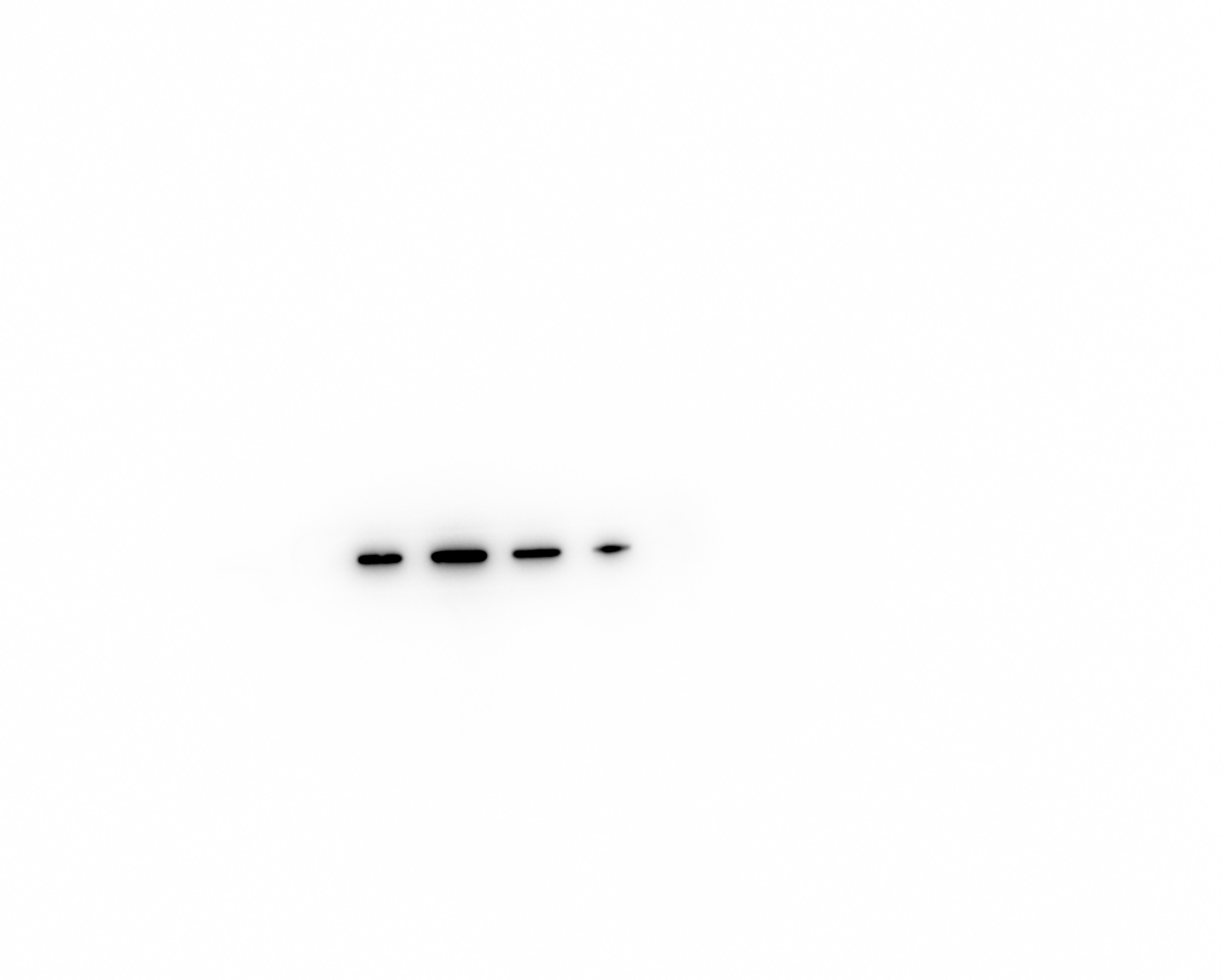

Supplement: DATA SHEET S1 — A full scan of the entire original gel(s). [file Data_Sheet_1.zip › original image files/Figure S2/T24/c-Myc.Tif]

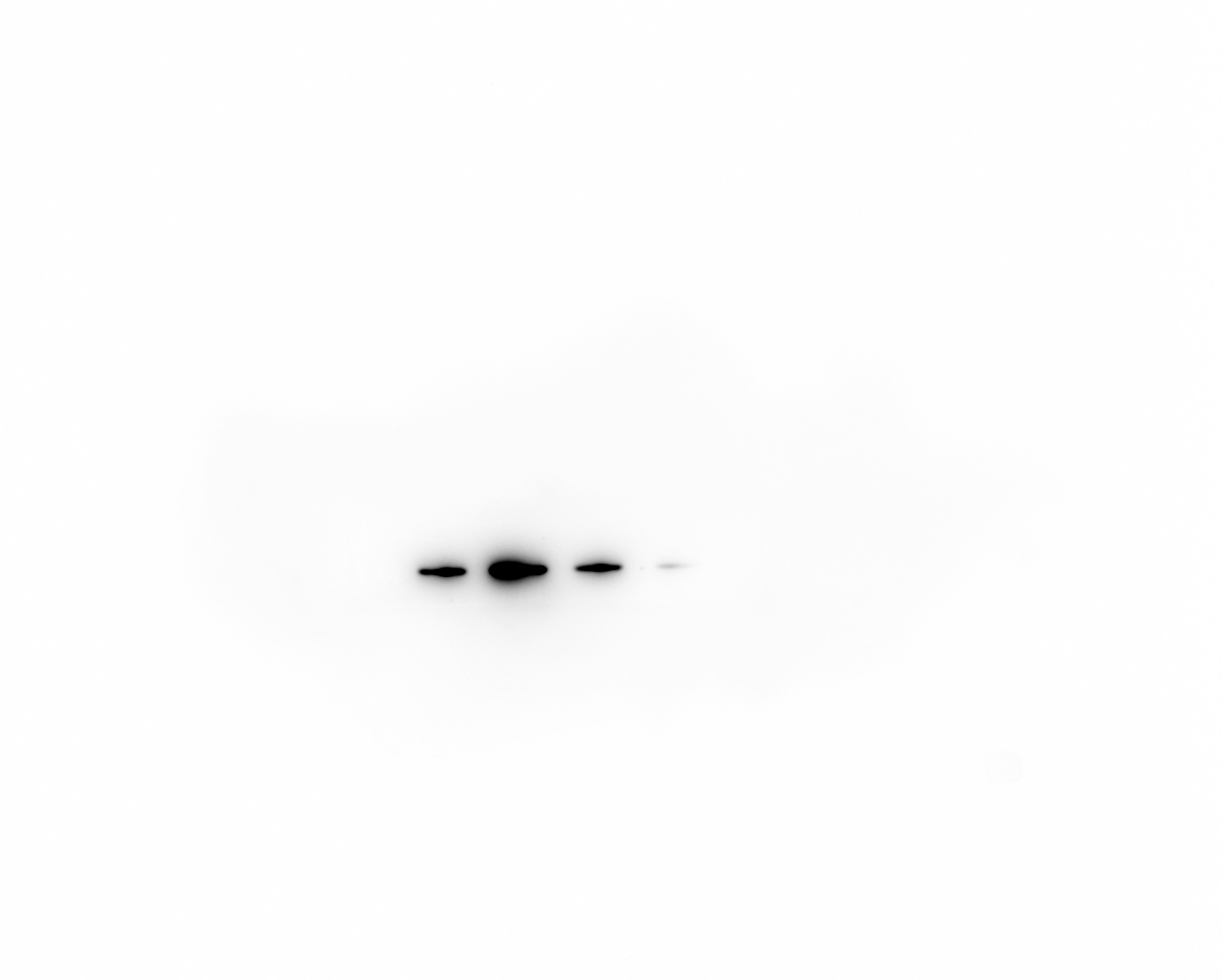

Supplement: DATA SHEET S1 — A full scan of the entire original gel(s). [file Data_Sheet_1.zip › original image files/Figure S2/T24/p-JAK2.Tif]

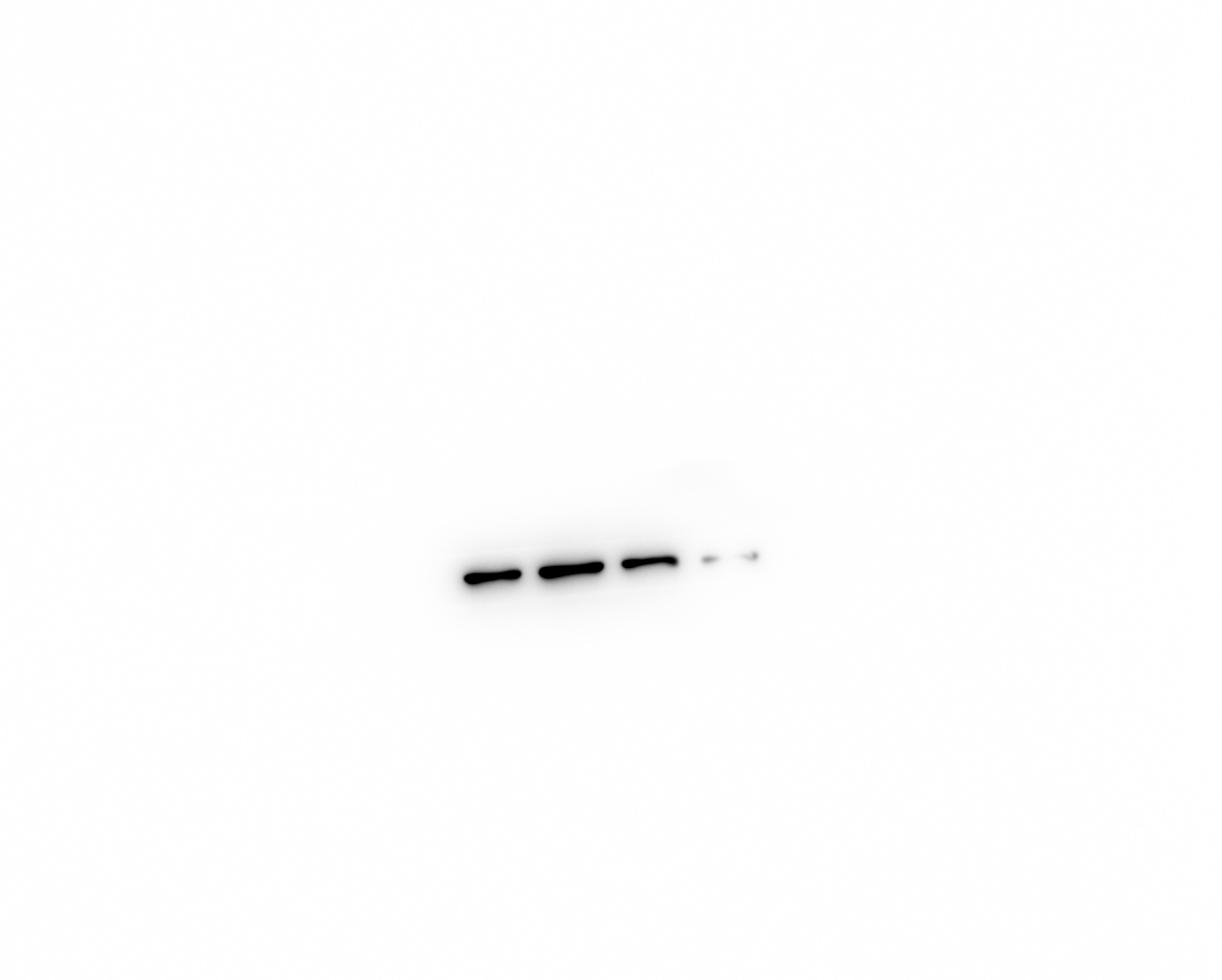

Supplement: DATA SHEET S1 — A full scan of the entire original gel(s). [file Data_Sheet_1.zip › original image files/Figure S2/T24/p-STAT3.Tif]
